# Long-read sequencing of the coffee bean transcriptome reveals the diversity of full-length transcripts

**DOI:** 10.1093/gigascience/gix086

**Published:** 2017-08-30

**Authors:** Bing Cheng, Agnelo Furtado, Robert J Henry

**Affiliations:** Queensland Alliance for Agriculture and Food Innovation, The University of Queensland, St Lucia, QLD 4072, Australia

**Keywords:** coffee, transcriptome, full-length cDNA, long sequences, isoform, polyploid, UTR

## Abstract

Polyploidization contributes to the complexity of gene expression, resulting in numerous related but different transcripts. This study explored the transcriptome diversity and complexity of the tetraploid Arabica coffee (*Coffea arabica*) bean. Long-read sequencing (LRS) by Pacbio Isoform sequencing (Iso-seq) was used to obtain full-length transcripts without the difficulty and uncertainty of assembly required for reads from short-read technologies. The tetraploid transcriptome was annotated and compared with data from the sub-genome progenitors. Caffeine and sucrose genes were targeted for case analysis. An isoform-level tetraploid coffee bean reference transcriptome with 95 995 distinct transcripts (average 3236 bp) was obtained. A total of 88 715 sequences (92.42%) were annotated with BLASTx against NCBI non-redundant plant proteins, including 34 719 high-quality annotations. Further BLASTn analysis against NCBI non-redundant nucleotide sequences, *Coffea canephora* coding sequences with UTR, *C. arabica* ESTs, and Rfam resulted in 1213 sequences without hits, were potential novel genes in coffee. Longer UTRs were captured, especially in the 5΄UTRs, facilitating the identification of upstream open reading frames. The LRS also revealed more and longer transcript variants in key caffeine and sucrose metabolism genes from this polyploid genome. Long sequences (>10 kilo base) were poorly annotated. LRS technology shows the limitation of previous studies. It provides an important tool to produce a reference transcriptome including more of the diversity of full-length transcripts to help understand the biology and support the genetic improvement of polyploid species such as coffee.

## Background

Polyploidy creates a complicated transcriptome with diverse transcript isoforms. As an important evolutionary process in plants, polyploidization generates new species and increases biodiversity [1]. A balance of genetic and biochemical features is required for the polyploid to survive while carrying multiple genomes in the same nucleus [2]. Genetic changes associated with the formation of polyploids include gene function, which may remain unchanged, or diversification among the multiple homeologs, leading to neofunctionalization, subfunctionalization, or pseudogenization [3]. Alternative splicing and polyadenylation also contribute further to the diversity of transcripts [4, 5]. Additionally, different 5΄UTRs account for transcript variation; however, limited information is available on this for most genes. This diversity may include different functional motifs, like upstream open reading frames, or introns harboured in this area, influencing post-transcription expression [6, 7]. All these influences contribute to the diversity and complexity of a polyploid transcriptome.

The transcriptome represents all the genes expressed in the cell or tissue. RNA sequencing (RNA-Seq) makes it possible to capture the identity of these genes. Generating a reference transcriptome is essential for studying variation in expression of genes and the influence of genotype or environment on their expression [8, 9]. Most studies generate a reference transcriptome by short-read sequencing and reconstruct the transcriptome by the assembly and/or mapping of reads to other available reference genomes [10–12]. However, this is difficult for long transcripts, repetitive sequences, and transposable elements. It is particularly challenging for complex polyploid genomes [13]. Long-read sequencing (LRS) technology (e.g., PacBio) has recently become available, and this technology overcomes these difficulties by generating sequence information for the full length as a single sequence read, including very long transcripts (e.g., those exceeding 10 kb) without the need for further assembly. This technique has been applied in a few plant studies and provides further information on transcript diversity, including alternative splicing and alternative polyadenylation [4, 5].

Arabica coffee is a recent allotetraploid (2n = 4x = 44; ∼50 000 years old) derived from *Coffea canephora* and *Coffea eugenioides*. A high-quality reference genome and annotation are not yet available for Arabica coffee. However, a draft genome is available for one of the sub-genomes, *C. canephora* [14]. Arabica coffee is highly regarded by coffee consumers, is of great economic value, and accounts for almost 70% of world coffee traded [15]. However, it is produced in limited high-altitude tropical environments and is threatened by climate change. Understanding the genetic and environmental control of coffee quality will be facilitated by the availability of detailed knowledge of the transcriptome of the coffee bean. This study used LRS by Pacbio Iso-seq to characterize the Arabica coffee bean transcriptome including beans from immature, intermediate, and mature stages in order to explore the complex polyploid system and establish a reference transcriptome for future studies of gene expression.

## Data Description

### RNA sample preparation

**Figure 1:**
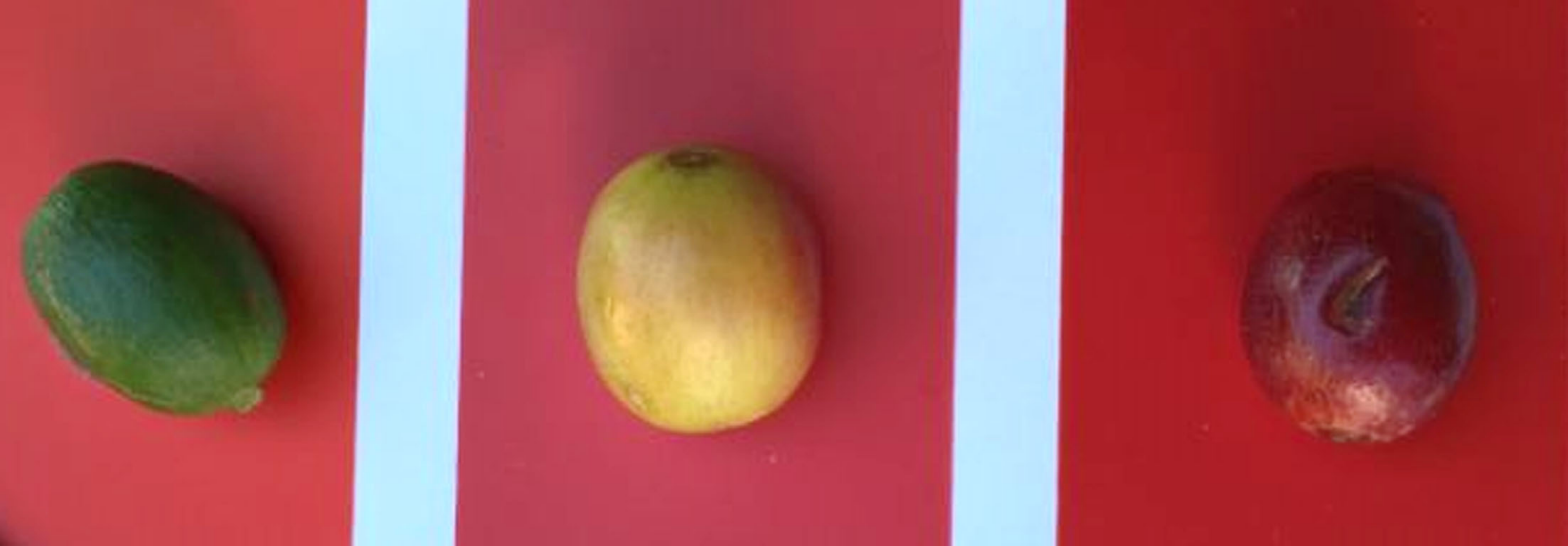
Coffee fruits of immature, intermediate, and mature stages.

Fruits at different development stages (immature, intermediate, and mature fruits) of *Coffea arabica* var. K7 (Fig. 1) were harvested from Green Cauldron Coffee, Federal, Australia. Five coffee trees were selected randomly, and ten coffee fruits (five fruits from the upper and lower canopies of each tree) were collected separately for each tree and each stage of development. Samples were collected in triplicate. In total, 450 coffee fruits (900 beans) from 15 trees were collected. Once each fruit was harvested, the pericarp was removed immediately with a scalpel in 20 seconds or less. The coffee beans were immediately frozen in liquid nitrogen, transported on dry ice and stored at –80°C until further use. Total RNA was extracted from coffee beans as described by Furtado [16]. Equal amounts of RNA from each of the 90 (three replicates of five trees at two levels in the canopy and three stages of development) extractions were combined to provide a representative sample for sequencing to generate a reference transcriptome. Afterwards, combined RNA was assessed for integrity using an Agilent RNA 6000 nano kit and chips on a Bioanalyzer 2100 (Agilent Technologies, Santa Clara, CA, USA) and processed further for cDNA preparation.

### cDNA preparation

The Pacbio Iso-seq protocol was used for cDNA preparation. cDNA was synthesized using a Clontech SMARTer PCR cDNA Synthesis kit (ClonTech, Takara Bio Inc., Shiga, Japan) and amplified using a KAPA HIFI PCR kit (Kapa Biosystems, Boston, MA, USA). The double-stranded cDNA was split into two sub-samples. One was used directly for sequencing. The other set was normalized to equalize transcript abundance and obtain rare sequences.

The cDNA was purified for normalization using a QIAquick PCR Purification Kit (Qiagen, Hilden, Germany). The purified cDNA was precipitated and normalized with a Trimmer-2 cDNA normalization kit (Evrogen, Moscow, Russia). The resulting cDNA was evaluated and quantified using an Agilent DNA 12 000 Kit and Chips on a Bioanalyzer 2100 (Agilent Technologies, Santa Clara, CA, USA). The same amount of non-normalized and normalized cDNA was used as input for Pacbio Iso-seq.

Samples were subjected to a Pacbio Iso-Seq protocol through purification, size selection (Blue Pippin system, Sage Science, Beverly, MA, USA), re-amplification, SMRTbell template preparation, and Iso-seq on a Pacbio RS II platform. A size selection protocol was applied as smaller cDNAs are more abundant and would otherwise be preferentially sequenced. Four Bluepippin bins were selected for non-normalized cDNA sequencing, with size ranges of 0.5–2.5, 2–3.5, 3–6.5, and 5–10 kb, respectively, since Pacbio sequencing preferentially sequences short DNA fragments. Two bins were selected for normalized cDNA sequencing, 2–3.5 and 3–6.5 kb, as the normalization biases against longer sequences.

### Raw read processing and error correction

Sequence data were processed through the RS IsoSeq (version 2.3) pipeline [17]. The first step was to remove adapters and artefacts to generate reads of insert (ROIs) consensus sequences. Short sequences of less than 300 bp were removed as the Bluepippin cDNA size selection starts from 500 bp, where some sequences of less than 500 bp have a chance to be sequenced. Non-Chimeric ROIs sequences were filtered into two groups of sequences comprised of full-length ROIs sequences and non-full-length ROIs sequences. Full-length (FL) ROIs sequences were identified based on the presence of the 5΄-adaptor sequence, the 3΄ adapter sequences (both used in the library preparation) and poly (A) tail. Further, FL ROIs sequences were passed through the isoform-level clustering (ICE). ROI sequences were used to correct errors (polish) in the isoform sequences using the Quiver software module. The polishing process of Quiver generated two isoform sequence files, one with high-quality (HQ) isoform sequences and the other with low-quality (LQ) isoform sequences corresponding to an expected accuracy of ≥99% or below, respectively. LQ output (or non-FL coverage sequences) is useful in some cases as it may result from rare transcripts or lower-coverage sequences. And these low-coverage sequences can be further used to correct errors in HQ output. The Primer IIA sequence motifs (used in the library preparation) that escaped removal at the ROIs stage corresponded to 11 sequences that were trimmed using CLC genomic workbench 9.0 (CLC, QIAGEN, CLC Bio, Denmark). After combining the HQ and LQ transcripts, further clustering was processed with CD-HIT-EST (c = 0.99) [18].

In the following step, the contaminant sequences were removed by CLC stepwise as follows [19]. (1) Chloroplast transcript sequences were identified by BLASTn to the *C. arabica* complete chloroplast genome (GenBank: EF044213.1). (2) Mitochondrial transcripts were characterized by BLASTn to *N. tabacum* and *V. vinifera* complete mitochondrial genomes (BA000042.1 and FM179380.1). (3) Ribosomal sequences were detected by BLASTn to the reported *C. arabica, C. canephora*, and *C. eugenioides* ribosomal genes (AJ224846, EU650386, DQ153609, AF416459, EU650384, EU650385, AF542981, AF542990, JX459583, JX459584, JX459585, JX459586, JX459587, DQ153593, AF542982, DQ423064, DQ153588, DQ153621, AF542986). (4) Virus, viroid, and prokaryote contaminants were identified with BLASTn to their reference genomes from the NCBI database (4 April 2017). Prokaryotic contaminants were screened with available reference genomes from NCBI (9 February 2017). (5) Fungal sequences were investigated by BLASTn to fungal proteins (4 April 2017). All the above analyses were processed one after another with a maximum E-value threshold of 1e-10.

From the BLASTn results, significant matches were filtered with a bit score (A) ≥300 as well as identity ≥80%. In each step, the filtered significant sequences were processed further with cloud BLASTn to the NCBI non-redundant database (bit score: B) to further confirm the matches. This validation step was confirmed by comparison of the bit score (comparison of values A and B). If the higher bit score was associated with a contaminant sequence in the BLASTn (A>B), then the sequence was discarded. In total, 526 sequences corresponding to chloroplastic (200), mitochondrial (264), ribosomal (37), viral (0), viroid (0), prokaryotic (0), and fungal (25) contaminant sequences, respectively, were removed in this process. Sequence quality was then accessed with the Fasta Statistics through Galaxy/GVL 4.0 [20]. This set of Iso-seq processed isoforms was used for further analysis and is hereafter called the “coffee long-read sequencing (coffee-LRS) isoforms” [21]. The term “isoforms” or “isoform sequence” or “transcript” used in this study represents individual sequences from the coffee-LRS isoforms, while “transcript variants” indicate different transcripts of a gene, including alternative spliced variants, homeologs, etc.

### Transcriptome annotation

A number of databases were used for annotation of the coffee-LRS isoforms described as follows [22]. (1) The plant Geninfo identifier (GI) list was downloaded from NCBI Protein Entrez (2 May 2017, 8 431 379 items). The plant proteins were retrieved from the NR database using this GI list, yielding 5 099 147 sequences (NR-plant). Then, the full set of the coffee-LRS isoforms was submitted to stand-alone BLASTx against the NR database below 1e-10. (2) Sequences without hits from step 1) were submitted further to NCBI non-redundant nucleotide sequences (NT, 5 May 2017) BLASTn at 1e-10. (3) Sequences without a hit from step 2) were processed further with BLASTn (1e-20) to *C. canephora* coding sequences (CDS) with UTR and *C. arabica* EST database [23, 24]. (4) The output of BLASTx was filtered with query coverage (Qcovs), cumulative identity (ID), and sequence length into three categories, high-, medium-, and low-quality annotation. Query coverage indicates the input coffee-LRS isoforms covered by the matched sequences. Cumulative identity represents the identity length to the aligned length (AL). ID can be expressed as the ratio of the sum of identity length to the sum of the aligned length of all the high-scoring segment pairs of a subject. The four databases above, NR plant, NT, *C. canephora* CDS with UTR, and the *C. arabica* EST database, are named as the FOUR databases in this manuscript. Finally, all the BLASTx and BLASTn results were processed by function annotation with BLAST2GO.

The Blast2GO Pro 4.0 (North America, US: USA2 Version: b2g_Sep 16) pipeline was based on default settings [25]. InterProScan/IPS was used to search sequence protein domains from EBI databases to improve annotation (North America, US: USA2, Version: b2g_Sep 16). In the follow-up phase, Blast2GO Mapping, Annotation, and Annex functions were applied to retrieve gene ontology (GO) terms, select reliable annotations, and increase the number of annotated isoforms, respectively. The GO-slim tool was used against the plant database to provide plant generic GOs. Finally, GO enzyme mapping and Kyoto Encyclopedia of Genes and Genomes pathway maps (KEGG pathways) were loaded.

### Case studies

Two case studies were performed with genes encoding caffeine and sucrose biosynthesis pathways (caffeine and sucrose genes) to investigate specifically the quality, advantage, and additional potential of the coffee-LRS isoforms. Reported coffee caffeine and sucrose candidate genes were downloaded from the European Nucleotide Archive (EMBL-EBI) (Tables 3 and 4).

For potential caffeine candidate genes, coffee-LRS isoforms were processed with BLASTn (1e-20) against the reported caffeine genes. Sequences with hits to the reported caffeine genes were submitted to BLASTx (1e-20) with the NR database to confirm whether they were caffeine genes (higher bit score). Confirmed transcripts (potential caffeine isoforms) and sucrose isoforms annotated by Blast2GO (potential sucrose transcripts) were further evaluated with Geneious 10.0.4 by aligning back to the reported candidate genes at the allele level [26]. Motif analysis was conducted with default parameters except for the “10 motifs” selected with MEME 4.11.2 [27]. UTRscan was used for UTR functional motifs annotation [28].

### Comparison to other available coffee databases

To compare with available coffee sequences, the full coffee-LRS isoforms were processed with BLASTn (1e-20) to *C. canephora* CDS with UTR and the *C. arabica* EST database, respectively, and the other way around [23, 24]. The *C. eugenioides* transcriptome (young leaves and mature fruits) from Illumina was also used in the comparison [29].

### Novel genes

Coffee-LRS isoforms without hits to the FOUR databases were submitted to the Rfam database by the Blast2GO Pro package for non-coding RNA analysis [30]. Sequences without hits to Rfam were probably novel genes in coffee.

### Analysis of long sequences

In order to explore the advantage of using the LRS PacBio platform to obtain long sequences, the BLASTx and BLAST2GO functional annotation results for the coffee-LRS isoforms longer than 10 kb were extracted from the total dataset.

## Analyses

### Overview of full-Length RNA molecules from long-read sequencing

A total of 2 618 905 raw reads were generated from LRS platform, which yielded 443 877 reads of insert. After 8842 short sequences (less than 300 bp) were removed, 233 464 full-length (FL) and 201 571 non-full-length (NFL) reads were generated. The individual isoforms were sequenced on average five times. In total, 95 995 coffee-LRS isoforms were recovered after sequences representing chloroplast, mitochondrial, and ribosomal transcripts were removed (Table 1). The length of the sequences in this dataset ranged from 301 bp to 23 335 bp, with an average length of 3236 bp. The GC content was 41.4%, and the N50 was 4865 bp.

**Table 1:** Arabica long-read sequencing transcriptome annotation with different databases

Databases	Number of sequences annotated	% of sequences annotated
Long-read sequencing transcriptome	95 995	-
BLAST	94 709	98.66
Mapped	78 571	81.85
InterProScan	70 774	73.73
InterProScan GOs	33 605	35.01
GO slim	58 050	60.47
KEGG	11 489	11.97

The BLASTx output (against NR plant) was divided into three groups, high, medium, and low quality based on Qcovs, ID, and sequence length (see the Data Description section). There were 34 719, 13 655, and 40 314 sequences were grouped into the high-, medium-, and low-quality groups, respectively (Supplementary Information 1: Table S1). Thereafter, 7280 sequences without hits were processed with BLASTn to NT database, resulting in 1981 sequences with hits. A total of 5299 sequences without a hit were further accessed with *C. canephora* CDS and UTR and *C. arabica* contigs. Finally, there were 1217 sequences with no hits to any of the above databases (FOUR databases).

**Table 2:** Arabica long-read sequencing isoforms compared to *Coffea canephora* coding sequences and *Coffea arabica* EST sequences

Different datasets	GC, %	N50, bp	Average length, bp	Min length, bp	Max length, bp	Number of sequences
*Coffea arabica* EST database^a^ [23]	44.7	734	662	32	3584	35 153
*Coffea canphora* coding sequences with UTR^b^	42.6	2046	1616	45	17 206	25 570
*Coffea arabica* long-read sequencing isoforms	41.4	4865	3236	301	23 335	95 995

^a^ http://bioinfo03.ibi.unicamp.br/coffea/data/CA.fasta.

^b^ http://coffee-genome.org/sites/coffee-genome.org/files/download/coffea_cds.fna.gz.

### Functional annotation

Functional annotation of the coffee-LRS isoforms was investigated using different databases. The data in Table 2 show that 88 715 sequences (92.42%) had hits to NR plant proteins. A total of 70 774 sequences (73.73%) matched to IPS protein domains with 33 605 IPS GOs (35.01%). A number of 78 571 sequences (81.85%) had identified GOs. After the GOs were merged, GOs of 58 050 sequences (60.47%) matched with GO-slim (plant).

Of all the hits to the NR plant proteins from BLASTx, the coffee-LRS isoforms (maximum 50 hits to each sequence) had the highest number of hits to the *Nicotiana tabacum* (tobacco, 1 746 308 hits), followed by *C. canephora* (142 656 hits), *Vitis vinifera* (grape, 134 025 hits), and *Theobroma cacao* (cacao, 132 336 hits) proteins (Supplementary Information 1: Fig. S1). Most hits found in tobacco were probably because the tobacco database is more extensive and well annotated than those of other related species, like *C. canephora*. For top-hit species, there is no doubt that the majority of the sequences has top-hit with the progenitor, *C. canephora* (73 587 sequences), followed by *Sesamum idicum* (1321 sequences), *Nicotiana tabacum* (767 sequences), etc. (Supplementary Information 1: Fig. S2). The NR-plant database consists of few proteins sequences from *Coffea arabica*, as reflected by just 485 protein sequence hits, and is ranked seventh in the top-species hit list. This indicates the limit information on *Coffea arabica*. Of the 33 512 sequences (34.91%) with IPS GOs, cytochrome P450 (IPR001128, 353 matches) had the most sequence matches among the IPS families (Supplementary Information 1: Fig. S3).

Biological process (BP, 56 230 sequences) was more abundant than cellular component (CC, 44 528 sequences) and molecular function (MF, 45 604 sequences) (Supplementary Information 1: Fig. S4). Within these functional groups, the highest number of sequences were annotated with the biosynthetic process (11 627 sequences, 20.68%), membrane component (21 175 sequences, 47.55%), and transferase activity (11 921 sequences, 26.14%). A total of 156 pathways with 921 enzymes were annotated by KEGG, associated with 11.97% of the whole dataset (11 489 sequences). Among these, starch and sucrose metabolism ranked as the fifth most abundant pathways, with 36 encoding enzymes and 766 isoforms annotated (Supplementary Information 1: Fig. S5). The average number of coffee-LRS isoforms encoding the 921 enzymes was 18, while the highest number was found in phosphatase (EC: 3.6.1.15, 2969 sequences) encoding the purine metabolism and thiamine metabolism pathway. In comparison, only 802 sequences were associated with 142 pathways and 374 enzymes in the *C. eugenioides* transcriptome, and the starch and sucrose pathway relating to 450 contigs was the most encoded pathway [29].

The candidate genes for the major caffeine candidate genes were not identified by KEGG pathway. To evaluate the annotated isoforms and their diversity, further analysis was performed with the caffeine pathway. The sucrose pathway was also analysed as a case study as sucrose candidate genes were relatively long and highly diverse. Both of these pathways are important for the understanding of coffee quality [31].

### Case study I: isoform diversity in the caffeine biosynthesis pathway

**Figure 2:**
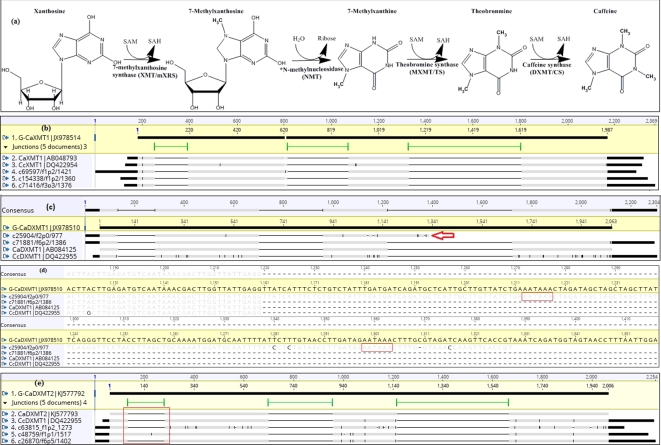
Putative transcript variants from long-read sequencing aligned to reference caffeine genes. (**a**) Main caffeine biosynthesis pathway in coffee, adaptive from Cheng, Furtado [31]. (**b**) Alignment of three Arabica putative XMT1 variants from long-read sequencing (c69597/f1p2/1412, c154338/f1p2/1360 and c71416/f3p3/1376), *Coffea arabica* and *Coffea canephora* XMT1 (CaXMT1 and CcXMT1) to Arabica XMT1 genomic DNA sequence (G-CaXMT1). (**c**) Possible alternative polyadenylation of putative XMT1 Iso-seq variant (c25904/f2p0977) from long-read sequencing; G-CaDXMT1, Arabica DXMT1 genomic DNA sequence; CaDXMT1, DXMT1 coding sequence. (**d**) Two polyadenylation signals were identified in 3΄ends of c25904/f2p0/977. (**e**) Possible alternative splicing (intron retention) in one of the putative DXMT2 variants (c48759/f1p1/1517) from long-read sequencing transcripts; G-CaDXMT2, Arabica DXMT2 genomic DNA sequence; CaDXMT2, Arabica DXMT2 coding sequence. (Note: black colour in the alignment means different nucleotides to reference sequence, Arabica genomic XMT1, while grey colour means the same nucleotides as the reference.).

The caffeine pathway has been widely studied previously (Fig. 2a). Candidate genes and complete coding sequences of both transcripts and genomic DNA are available in public databases and can be used as well-established references for caffeine candidate gene analysis (Table 3). From the BLASTn output, 25 long-read transcripts were annotated and related to candidate caffeine genes [21]. Further alignment suggests that ten high-quality isoforms were likely to be putative caffeine genes, including three transcript variants of *XMT1*, one of *MXMT1*, one of *MXMT2* together with two of *DXMT1*, and three of *DXMT2*. All genes encoding caffeine as the primary pathway, except the *XMT2* gene, were present in this bean transcriptome (Fig. 2 and Table 3). The length distribution of these isoforms ranged between 977 and 1517 bp.

**Table 3:** Details of caffeine candidate genes, putative transcript variants annotated, and 5΄UTR extension information

Candidate genes	Accession number	Species	Source	Abbreviation	Length, bp	Completeness	Putative transcript variants from LRS isoform sequences	5΄UTR extension
Xanthosine	AB048793	*C. arabica*	mRNA	CaXMT1	1316	YES	c69597/f1p2/1421	YES
methyltransferase 1	JX978514	*C. arabica*	Genomic DNA	G-CaXMT1	1987	YES	c154338/f1p2/1360	
	DQ422954	*C. canephora*	mRNA	CcXMT1	1316	YES	c71416/f3p3/1376	
	JX978509	*C. canephora*	Genomic DNA	G-CcXMT1	1994	YES		
Xanthosine methyltransferase2	JX978515	*C. arabica*	Genomic DNA	G-CaXMT2	2038	YES	Not identified	-
7-methylxanthine	AB048794	*C. arabica*	mRNA	CaMXMT1	1298	YES	c20397/f5p1/1361	YES
N-methyltransferase 1	JX978511	*C. arabica*	Genomic DNA	G-CaMXMT1	1838	YES		
	HQ616707	*C. canephora*	mRNA	CcMXMT1	1222	YES		
	JX978507	*C. canephora*	Genomic DNA	G-CcMXMT1	1829	YES		
7-methylxanthine	AB084126	*C. arabica*	mRNA	CaMXMT2	1155	YES	c10402/f2p3/1277	YES
N-methyltransferase 2	JX978512	*C. arabica*	Genomic DNA	G-CaMXMT2	2010	YES		
3,7-dimethylxanthine	AB084125	*C. arabica*	mRNA	CaDXMT1	1155	YES	c25904/f2p0/977	YES
N-methyltransferase 1	JX978510	*C. arabica*	Genomic DNA	G-CaDXMT1	2063	YES	c71881/f6p2/1386	
3,7-dimethylxanthine	KJ577793	*C. arabica*	mRNA	CaDXMT2	1155	YES	c63815/f1p2/1273	YES
N-methyltransferase 2	KJ577792	*C. arabica*	Genomic DNA	G-CaDXMT2	2006	YES	c48759/f1p1/1517	
	DQ422955	*C. canephora*	mRNA	CcDXMT1	1364	YES	c26870/f6p5/1402	

Importantly, all ten isoforms were extended at the 5΄ UTR region compared to the corresponding sequences reported in Arabica and Robusta coffee (Table 3), while eight isoforms were longer at the 3΄ end (Fig. 2b, c, e; Supplementary Information 2: Fig. S6). The most extended isoform (c695597/f1p2/1421) was 136 bp longer than the previously reported candidate genes (*CaXMT1*, Fig. 2b). Nine isoforms were found to be longer than the reported genomic DNA sequences. The other isoform was likely to have resulted from an alternative polyadenylation event (c25904/f2p0/977) (Fig. 2c) as two potential polyadenylation signals (AAUAAA) were identified in the 3΄ UTR (Fig. 2d). Alternative splicing was also presented in caffeine isoforms; e.g., intron retention was detected in one of the putative *DXMT2* isoforms (Fig. 2e).

Coffee-LRS isoforms encoding XMT1 (Fig. 2b), MXMT1 (Supplementary Information: Fig. S6), and DXMT2 (Fig. 2c, e) were better aligned to the corresponding *C. canephora* isoforms individually (higher identity) (Fig. 2; Supplementary Information: Fig. S6). This indicates that these transcript variants were potentially *C. canephora* sub-genome copies. In contrast, isoforms encoding XMT2 (Fig. 2c), MXMT2 (Supplementary Information: Fig. S6), and DXMT1 (Fig. 2c, e) were poorly aligned with *C. canephora* isoforms (more variants) and were probably *C. eugenioides* sub-genome copies.

### Case study II: long sucrose isoforms provide insight into the complexity of the polyploid system

**Figure 3:**
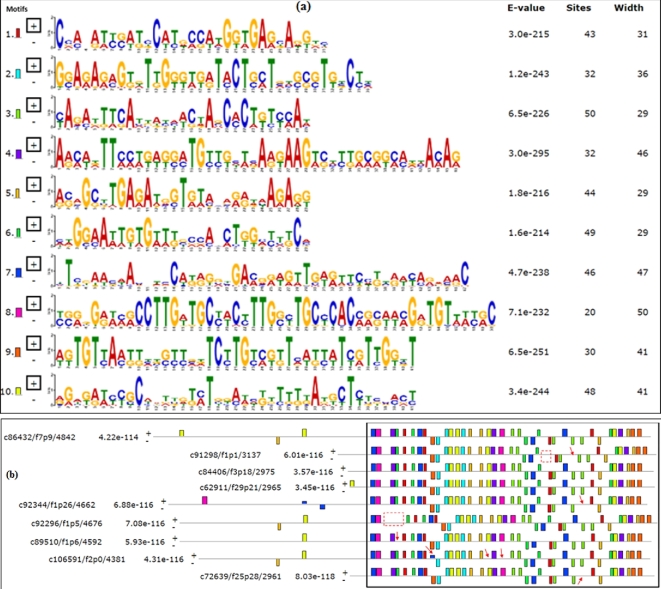
Motif search results of putative sucrose synthase gene 1 from long read sequencing. (**a**) Ten motifs were annotated in 9 putative sucrose synthase 1 variants from long-read sequencing, analysed by MEME 4.11.2. (**b**) Motif location of 9 putative sucrose synthase 1 variants. Different motifs were highlighted with red arrows and intron retention was shown with dashed boxes.

**Figure 4:**
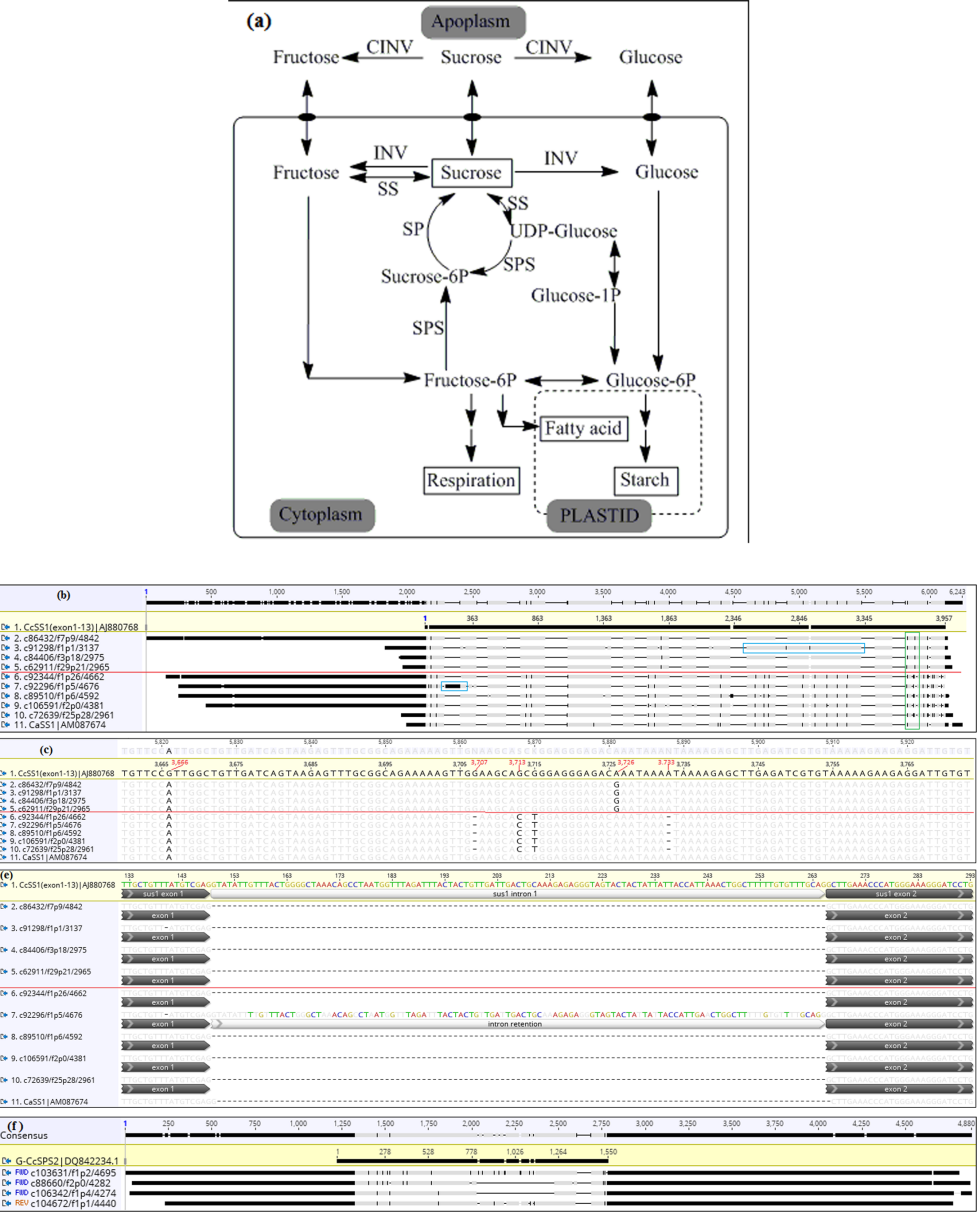
Putative variants from long-read sequencing aligned to the reference sucrose genes. (**a**) Possible sucrose metabolism in coffee; SS, sucrose synthase; SPS, sucrose phosphate synthase; SP, sucrose phosphatase; INV, invertase; CINV, cell wall invertase (modified from Cheng B. et al. (2016)). (**b**) Alignment of nine Putative Sucrose synthase variants from long-read sequencing and *C. arabica* sucrose synthase gene 1 (CaSS1) to *Coffea canephora* genomic sucrose synthase 1 (exons 1–13) (G-CcSS1 (1–13)); Green boxes highlights variants result from different sub-genome copies, while intron retention events were marked with the blue boxes. (**c**) Polyploid expression when zooming green area in 100%. (**d**) Possible alternative splicing (intron retention) from a *C. canephora* sub-genome copy when zooming blue box in 100%. (**e**) Possible intron retention from a *C. eugenioides* sub-genome copy when zooming blue area in 100%. red line classifies two groups of variants as different sub-genome copies. Different nucleotides compared to the consensus were highlighted in black in the alignment. (**f**) Putative variants from long read sequencing aligned with *C. canephora* genomic sucrose phosphate synthase. 2 sequence (G-CcSPS2); FWD, forward sequence; REV, reverse sequence. Different nucleotides compared to the consensus were highlighted in black in the alignment.

Sucrose genes were used to investigate the transcriptome sequence diversity of the polyploidy system. For the sucrose synthase 1 gene (*SS1*), one of the important genes in the sucrose metabolism, nine transcript variants were identified (Table 4 and Figs 3 and 4a). Compared to c86432/f7p9/4842, the other eight transcript variants varied in motif replacement (motif 7 replaced motif 9 in c106591/f2p0/4381), deletion (e.g., c92344/f1p26/4662), and relocation (intron retention, c92296/f1p5/4676 and c91298/f1p1/3137), etc. (Fig. 4b). The length of these nine putative *SS1* transcript variants ranged from 2961 to 4842 bp.

**Table 4:** Details of sucrose candidate genes, putative transcript variants annotated and 5΄UTR extension information

Candidate genes	Accession number	Species	Source	Abbreviation	Length, bp	Completeness	Putative transcript variants from LRS isoform sequences	5΄UTR extension
Sucrose synthase 1	AM087674.1	*C. arabica*	mRNA	CaSS1	2979	YES	c86432/f7p9/4842	YES
							c91298/f1p1/3137	
							c84406/f3p18/2975	
	DQ834312.1	*C. canephora*	mRNA	CcSS2	2989	YES	c62911/f29p21/2965	
							c92344/f1p26/4662	
							c92296/f1p5/4676	
	AJ880768.2	*C. canephora*	Genomic DNA	G-CcSS1	3957	exon 1–13	c89510/f1p6/4592	
							c106591/f2p0/4381	
							c72639/f25p28/2961	
Sucrose synthase 2	AM087675.1	*C. arabica*	mRNA	CaSS2	2889	YES	c73322/f3p2/3080	YES
	AM087676.1	*C. canephora*	Genomic DNA	G-CcSS2	5672	exon 1–15	c75363/f3p2/2906	
Sucrose phosphate	DQ834321.1	*C. canephora*	mRNA	CcSPS1	3150	YES	c51110/f2p0/3136	YES
synthase 1	DQ842233.1	*C. canephora*	Genomic DNA	G-CcSPS1	8215	YES		
Sucrose phosphate	DQ842234.1	*C. canephora*	Genomic DNA	G-CcSPS2	1550	NO	c103631/f1p2/4695	YES
synthase 2							c88660/f2p0/4282	
							c106342/f1p4/4274	
							c104672/f1p1/4440	
							(reverse)	

Importantly, all the sucrose transcript variants studied in this research were extended in the 5΄UTR region relative to previous reports, except for *SPS1*, c51110/f2p0/3136 (Table 4). Some transcript variants, such as the longest putative *SS1* sequence identified, c86432/f2p7/4842 (4842 bp), extended 2131 bp upstream of the *C. canephora* coding sequence (*G-CcSS1*) and 1994 bp upstream of the Arabica sucrose synthase 1 mRNA coding sequence (*CaSS1*). The length of the 5΄leading region of the *SS1* transcript variants ranged between 218 and 2131 bp (Table 5). To understand the diversity in this region, the 5΄ leading sequences of the nine putative *SS1* transcript variants were scanned using the UTRdb online server. A maximum of 12 upstream open reading frames (uORFs) were identified, and the number was positively correlated with the length of the sequences. No uORFs were identified in the two transcript variants with short 5΄UTR, c62911/f29p21/2965 (218-bp leader sequence), and c72639/f25p28/2961 (232-bp leader sequence).

**Table 5:** Results of 5΄ UTRs from long-read sequencing scanned with UTRdb

No.	Sequence name	5΄ UTR length, bp	uORF
1	c86432/f2p7/4842	2131	12
2	c91298/f1p1/3137	347	2
3	c84406/f3p18/2975	242	2
4	c62911/f29p21/2965	218	0
5	c92344/f1p26/4662	1981	10
6	c92296/f1p5/4676	1884	12
7	c89510/f1p6/4592	1871	11
8	c106591/f2p0/4381	1683	11
9	c72639/f25p28/2961	224	0

The nine *SS1* transcript variants revealed transcript diversity that resulted largely from different copies from the progenitors. When aligned to *G-CcSS1* (*C. canephora SS1* genomic sequence), the top four putative *SS1* transcript variants showed high identity and consistent nucleotide variants (like the guanine highlighted at 3726 bp in the consensus sequence) (Fig. 4c), suggesting that these were copies from the *C. canephora* sub-genome. For example, compared to the consensus sequence, the same indels were present in 3707 bp and 3733 bp, a cytosine at 3713 bp, and guanine at 3715 bp, etc. Consistently, the sequence of intron retention in one of the top four sequences, c91298/f1/p1/3137 (Fig. 4d) shows high homology to the intron sequence of *C. canephora*. However, the bottom five transcript variants had a higher number of variations compared to *G-CcSS1* that are likely to be *C. eugenioides* sub-genome-derived copies. The lower five transcripts had lots of variations compared to *C. canephora* intron 10, further indicating that this group was from a different copy, probably *C. eugenioides* (Fig. 4e). Additionally, some alleles of *G-CcSS1* were common in nine putative Arabica *SS1* transcript variants and Arabica sucrose synthase 1(*CaSS1*), such as the variant at 3666 bp (Fig. 4e). This type of allele probably results from different genotypes. Polyploid expression patterns were also observed in *SP1* transcript variants, the top two alignments were similar to *C. canephora*, and the other two were slightly different but related. All of the four transcript variants were longer in the upstream sequences while three extended further downstream than had previously been reported.

Another essential potential of LRS is to explore sequences not yet complete or published. For instance, four transcript variants were identified from this research while *SPS2* has only been identified in *C. canephora* rather than *C. arabica* (Fig. 4f).

### Comparison to other available coffee databases

**Figure 5:**
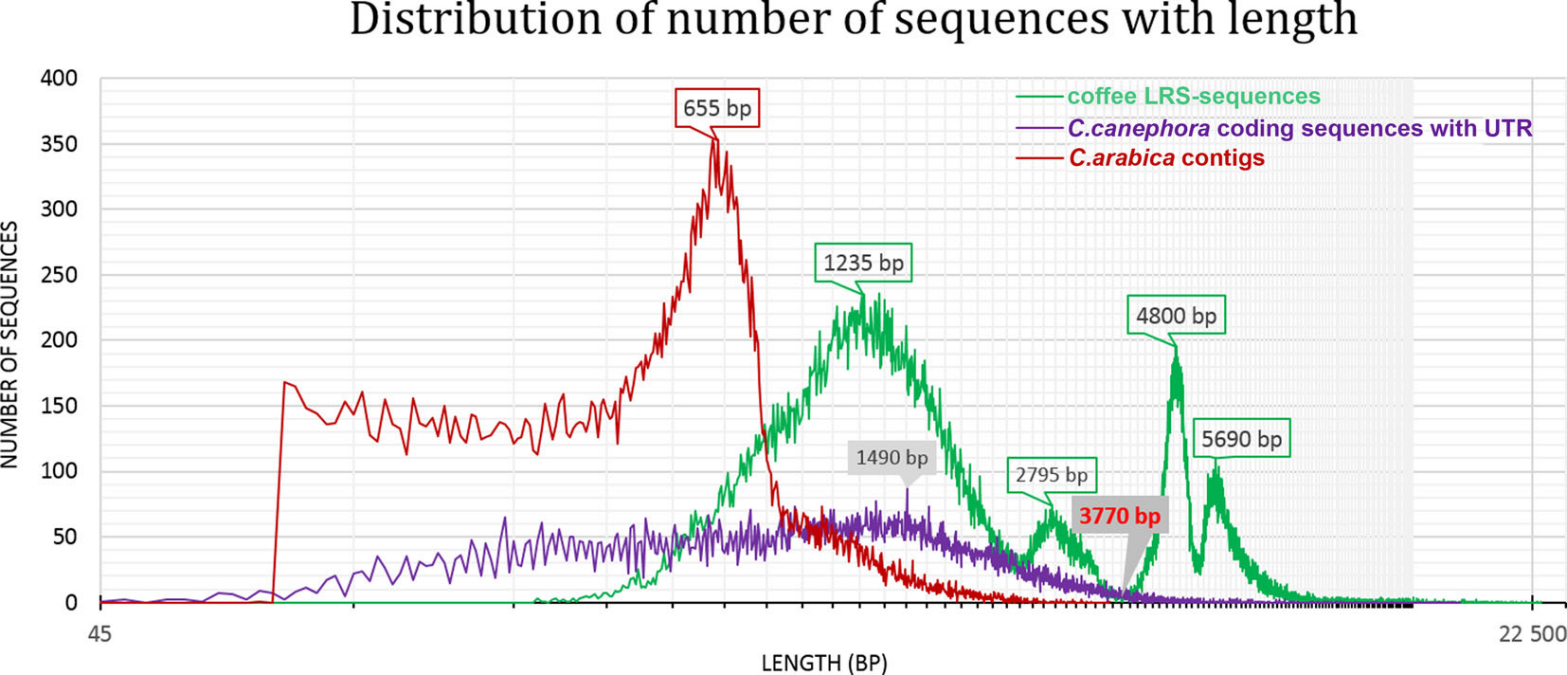
The distribution of the number of coffee long read sequencing sequences (coffee LRS-sequences), *C. canephora* coding sequences with UTR, *C. arabica* contigs with length. The horizontal axis is formatted in logarithmic scale.

To understand the advantage and the diversity of this polyploid coffee transcriptome, a comparison was made with the available coffee database. More than twice the number of isoforms was identified in the tetraploid Arabica LRS transcriptome (immature, intermediate, and mature fruits) compared with the *C. eugenioides* contigs (36 935 *de novo* assembled contigs, average length: 701 bp, from immature leaves and mature fruits), *C. canephora* CDS with UTR (25 570 sequences, from a variety of tissues, including fruits), and the *C. arabica* EST database (35 153 contigs, including fruits) (Table 2) [14, 23, 29]. The coffee-LRS isoforms show greater transcript length and diversity and a lower GC content. The N50 of the Pacbio dataset (4865 bp) was more than 3 times longer, and the average length was more than twice that of the other databases. The sequence distribution of *C. arabica* contigs peaks at 655 bp while *C. canephora* CDS with UTR reaches the largest number of sequences at 1490 bp (Fig. 5). Most of the sequences from the *C. canephora* CDS with UTR and the *C. arabica* EST database were less than 3770 bp. By comparison, 39 917 coffee-LRS isoforms (41.6%) were longer than 3770 bp.

Results of the BLASTn analysis indicated that of the 95 995 coffee-LRS isoforms, 9308 (9.7%) had no matches to the *C. canephora* CDS with UTR while 3682 (3.8%) isoforms had no hits to the *C. arabica* contigs. This indicates that coffee-LRS isoforms are very diverse compared to these two databases. Conversely, 9167 (26.1%) of *C. canephora* CDS with UTR and 4830 (18.9%) of *C. arabica* contigs had no hits to the coffee-LRS isoforms. These two sets of sequences without hits are probably sequences from leaf or other tissues not expressed in the tissues investigated in this study.

### Novel genes

The 1217 sequences without hits to the FOUR databases (NR plant proteins, NT database, *C. canephora* CDS with UTR, and *C. arabica* EST database) were submitted to the Rfam server to predict non-coding RNAs (ncRNA). The four isoforms that matched were in three biotypes; two transcripts were identified as CD-box snoRNA, one as HACA-box snoRNA and the other as an miRNA (Supplementary Information: Table S2). Other than these, the other 1213 sequences had no hit to the FOUR databases, and Rfam are likely to be novel genes that have not been discovered in coffee or contaminants from other organisms with no sequence information to date [21]. The length distribution of this new dataset ranged from 325 to 19 189 bp.

### Long transcripts

In order to assess the value of LRS in discovering long sequences, 577 transcripts longer than 10 kb were further analysed. Functional annotation of this extremely long dataset shows that the majority of the sequences (564 sequences, 97.8%) matched to the FOUR databases [21]. The HSP/Hit coverage distribution was relatively evenly distributed from 0% to 100% compared to the HSP/Seq coverage. In parallel, the majority of sequences distributed less than 50% HSP/Seq coverage and peaked at 6%, representing limited information of long sequences in the NR database. IPS matches were found for 352 sequences (61.0%) while 61 of them had IPS GOs. A total of 446 sequences (77.3%) were retrieved with GO terms, while 201 isoforms (34.8%) from these were also annotated with GO-Slim.

In total, 144 sequences were classified into the biological process, with 92 sequences into cellular component and 79 into molecular function (Supplementary Information: Table Fig. S7). Among them, biosynthetic process (31 sequences), member (43 sequences), and hydrolase activity (25 sequences) were the top groups, separately, from the three functional process. Among the annotated isoforms, 18 sequences encoding 12 enzymes from 13 pathways were annotated with a KEGG pathway. The starch and sucrose metabolism ranked the third most encoded pathway, with two isoforms encoding two enzymes.

## Discussion

Full-length transcripts generated by LRS in this study provided an isoform-level polyploid coffee bean reference transcriptome. Compared to its sub-genome progenitors, the Arabica coffee bean transcriptome was more diverse and complicated, with more isoforms, enzymes, and pathways. Case studies in caffeine and sucrose identified that this diversity and complexity were a result of alternative splicing, polyadenylation, 5΄UTR extension, and sub-genome copies. Discovery of novel genes and long transcripts was also an advantage of using the LRS technology.

### Polyploid expression

Different transcript variants may vary in function within the cell and be differentially expressed in tissues or environmental conditions. The abundance of variants in the Arabica transcriptome and case studies of caffeine and sucrose genes compared to the sub-genome progenitors clearly shows the complexity of the polyploid expression.

Generally, polyploidy results in 3 main expression patterns of non-additive expression: dominant expression in which total gene expression in the hybrid is similar to one of the parents, transgressive expression compared to the progenitors, or unequal homeolog expression [1]. Previously, it was proposed in coffee that the lower-caffeine in Arabica coffee was due to the *C. eugenioides* sub-genome attributes. Based on phylogenetic analysis, CaXMT1, CaMXMT1, and CaDXMT2 were believed to be from the *C. canephora* sub-genome while CaXMT2, CaMXMT2, and CaDXMT1 were from the *C. eugenioides* sub-genome [32]. *C. eugenioides* has a very low caffeine biosynthesis, together with a rapid catabolism [33]. The expression of sub-genome copies from *C. eugenioides* suggested lower caffeine in Arabica coffee compared to Robusta coffee. This study supports this hypothesis of transcript variants from sub-genome copies controlling the trait.

Using the LRS isoforms, further studies are now possible at the isoform level (this study was at the transcript variant level) to understand sub-genome gene expression in the polyploid *C. arabica*. First, it will be possible to determine directly whether the expression of Arabica caffeine genes follows a non-additive expression pattern. Second, it would be interesting to determine the reason for more transcript variant–identified changes in differential expression in tissues and at development stages. Third, it will be possible to determine whether this expression pattern is influenced by environment, influencing coffee quality. Fourth, it will be possible to determine whether these different gene expression patterns result in different phenotypes. Similar analysis could be applied to many other genes or pathways of interest. Isoforms and transcript variants found in the LRS tetraploid Arabica coffee bean transcriptome in this study were assigned to a number of functional groups, pathways, and specific enzyme functions. Arabica is believed to be more adaptive to temperature change than its diploid parents [34]. This study may also help elucidate the genetic basis of the higher sucrose in Arabica coffee. More generally, the complete polyploid transcriptome from this study will improve our understanding of the evolutionary adaptation and plasticity of polyploid species. However, further improvement is still needed in LRS technologies to improve the sequencing depth. Candidate genes in the caffeine pathway are reported to be expressed at low levels in fruits compared to leaves, especially XMT2 (detected by quantitative real-time polymerase chain reaction) [32]. Transcripts were not detected in this study, probably due to low expression of XMT2 and the PacBio iso-seq technology not being sensitive enough to capture these transcripts. This is likely to happen in the case of other isoforms expressed at low levels that may not be captured by the Iso-Seq technology even after application of the cDNA library normalization step, as was applied in this study.

### 5΄UTR extension

Full-length transcripts captured in this study show the advantage of LRS. All the caffeine and sucrose isoforms annotated in this study, except for *SPS1*, were extended in the 5΄UTR compared to those available from public databases. Previously, it was difficult to sequence the 5΄ end as cDNA library preparation starts from the 3΄ end and normally fails to reach the 5΄ end. Further, it was not easy to assemble the non-coding parts of transcripts as limited cDNA sequence was available to guide the assembly and confirm the contigs obtained. Therefore, less information is available on the 5΄UTRs, especially for plants. Generally, the length of the 5΄UTR ranges from 100 up to a few thousand bp [35]. This length difference is proposed because of the complex gene regulation maintained in eukaryotes [36]. Few post-transcriptional mechanisms have been studied in 5΄UTRs, including the regulation by the pre-initiation complex and uORF re-initiation.

uORFs are common in 5΄UTRs that have critical regulation. They contain their own set of start and stop codons that can be scanned by ribosomes and translated. This regulation can inhibit translation of the main ORF transcript and reduce the amount of protein translated. Regulation of re-initiation of uORF translation was found to be associated with the length of sequence between the uORF and the main ORF, suggesting that interactions with translation factors are required before initiation of translation [37]. This was also shown to be influenced by stress conditions [37]. However, not all uORF may have a role in translation control. In the leucine zipper transcription factor (*bZIP*) 11 gene, e.g., harbouring 4 uORFs, only uORF2 was required for this regulation, and this uORF is relatively conserved [38]. Other types of 5΄UTR regulation may also be found such as that due to introns in the 5΄UTRs. This happens to be approximately 35% of human genes [6].

Understanding the mechanism of 5΄UTR regulation will be greatly facilitated by the use of the full-length transcripts. In this study, multiple uORFs were characterized in the *SS1* 5΄ UTR, and these may contribute to diverse functions and regulation that may be influenced by stress conditions. Climate change is a threat to Arabica coffee, which grows at high altitude. It may be possible to influence 5΄UTR regulation in Arabica coffee and have the potential to influence coffee quality. To confirm this, further phenotype, proteome, and metabolome studies are required.

### Long transcripts

LRS also has potential in discovering long transcripts, such as the sucrose synthase genes annotated here. Even though numerous studies have defined the sucrose pathways, not all the candidate genes have been identified. Many sucrose metabolism genes are too long to be captured by short-read sequencing without significant *de novo* assembly. For example, the *C. arabica SS2* coding sequence is 2889 bp, and the genomic DNA sequence (exon 1 to 15) is 5672 bp (Table 4). Sucrose synthase genes (6–7 different isoforms) were previously identified in cotton, rice, and Arabidopsis, However, in coffee, only two had been reported [39–41]. For genes that were only previously available for *C. canephora* (e.g., *SP1*), this study also identified isoforms in Arabica. For genes that previously only had partial sequences available (e.g., *SPS2*), the transcripts identified in this study will guide further studies and improve current databases. Furthermore, the low coverage annotation of long sequences (>10 kb) by BLASTx and BLASTn against the FOUR databases indicated the limited information on long sequences, requiring further study.

### Transcriptome analysis of polyploids using long-read sequencing

LRS technologies show advantages in understanding complex transcriptomes, especially from polyploid species [4, 42, 43]. First, this eliminates transcriptome reconstruction, and that reduces the computation time. This is an essential goal for bioinformatics data analysis and software development [44]. To avoid obsolescence, transcriptome analysis calls for rapid genomics and bioinformatics to reduce the time from experiment to publication. Secondly, as there is no assembly of reads with LRS, there are no erroneous results due to misassemblies caused by complex polyploid transcriptomes with a large number of repeats or homeolog genes. For example, almost 80% of the wheat genome is repetitive [43]. Last but not least, it shows the potential to capture rare or long sequences to provide an overview of the transcriptome and fully characterize RNA diversity, like 5΄UTR extension in this study, alternative splicing, polyadenylation, etc. [4, 45].

However, LRS technologies have been normally biased with high error rates; e.g., previously released PacBio single molecule real-time sequencing (SMRT) reads had a very high error rate, 11–14%, therefore, numerous methods have been proposed to correct the sequences [46]. One common approach was to map back to a reference genome and/or use hybrid sequencing, e.g., using short reads with high throughput to correct LRS isoform sequences [5, 47]. However, caution is necessary when using this strategy. The reference genome is often far from 100% accurate: (1) most draft genomes have numerous fragmented contigs or scaffolds with huge imbedded gaps. Even genomes previously considered well assembled have had many gaps [48]. (2) Problems also exist in poorly assembled gene loci. Few recently released genomes have been re-visited to generate improved assemblies [13]. (3) LRS isoform sequences normally come from different sources (e.g., genotype) to the reference genomes that they can be compared with. Hybrid sequencing correction may have system bias and result in loss of isoforms/transcript variants or generate a “compromised” consensus. Previously, it has been estimated that there is no approach that has achieved more than 60% accuracy for transcript reconstruction, even for the most studied human genome [49]. For instance, short-read platforms deliver data that are less representative of rare or long isoforms, and there is a high chance of losing these reads from the long-read dataset when correcting.

Improved accuracy may be generated from the platform itself; e.g., Pacbio Iso-seq generates improved accuracy from CCS reads. This allows multiple passes of each transcript. Each pass can be used to correct the others with their random errors (mainly indels). The isoform clustering and polishing in this protocol are expected to deliver 99% accuracy. Prior to size selection, normalization was further applied in parallel to the dataset in this study to decrease the frequency of abundant reads and produce a more even representation of the transcriptome and to capture rare sequences. A highly diverse transcriptome has resulted. The abundance of genes that had not been previously sequenced (1213), transcript variants, and longer isoforms indicate the limits of previous studies and potential of LRS technologies. However, the limitation shows in detecting short sequences of less than 300 bp (raw data cut-off). The chances of large errors due to indels from Pacbio sequencing may produce reads shorter than the actual reads. Additionally, the Blue pippin size selection system starts from 500 bp in the cDNA library preparation, with few sequences from the boundary (400–500 bp). Therefore, improvement is needed to capture a broader transcriptome.

In conclusion, this study will improve the understanding of the biology and genetic improvement of polyploid species such as coffee. It provides a useful technique to generate a full-length reference transcriptome and improve understanding of UTR regions.

## Additional information

New sequence data used in this manuscript has been submitted to European Nucleotide Archive at EMBL database with accession number PRJEB19262. Supporting data are also available via the *GigaScience* repository, *Giga*DB [21]. Additional information on specific selected sequence IDs, such as high-quality annotated sequences, novel genes in coffee, etc., are shown in Supplementary Information 2:. The python script to calculate cumulative identity and alignment length has been submitted to Github (https://github.com/chengbing0404/BLAST5_result_handle).

## Additional files

supporting information 1-for 3rd submission.pdf

supporting information 2-for 3rd submission.xlsx

## Abbreviations

AL: alignment length; BP: biological process; bp: base pairs; Ca: coffea arabica; CC: cellular component; Cc: coffea canephora; CDS: coding sequences; Ce: coffea eugenioides; EST: expressed sequence tag; FL: Full-length; FOUR databases: NR plant, NT, Coffea canephora CDS with UTR and Coffea arabica EST database; GI: geninfo identifier; GO: gene ontology; HQ: high qulaity; Hsps: high-scoring segment pairs; ICE: isoform-level clustering; ID: cumulative identity; Iso-seq: Isoform sequencing; kb: kilo base; LQ: low quality; LRS: Long read sequencing; MF: molecular function; NFL: non Full-length; NR-plant: NCBI non redundant plant proteins; NT: NCBI non-redundant nucleotide sequences; Qcovs: query coverage; ROIs: reads of insert; RNA-Seq: RNA sequencing; uORFs: upstream open reading frames.

## Completing interests

All authors have no conflicts of interest related to this manuscript.

## Funding

This study was funded by Australian Research Council (PROJECT ID: LP130100376) and Chinese Scholarship Council (2014–2018).

## Author contributions

B.C., A.F., and R.H. designed the research and discussed the results. B.C. performed the experiment and analysis. B.C. drafted the manuscript. R.H. and A.G. refined it.

## Supplementary Material

GIGA-D-17-00024_Original-Submission.pdf

GIGA-D-17-00024_Revision-1.pdf

GIGA-D-17-00024_Revision-2.pdf

GIGA-D-17-00024_Revision-3.pdf

GIGA-D-17-00024_Revision-4.pdf

Response-to-Reviewer-Comments_Original-Submission.pdf

Response-to-Reviewer-Comments_Revision-1.pdf

Response-to-Reviewer-Comments_Revision-2.pdf

Response-to-Reviewer-Comments_Revision-3.pdf

Reviewer-1-Report-(Original-Submission).pdf

Reviewer-1-Report-(Revision-1).pdf

Reviewer-2-Report-(Original-Submission).pdf

Reviewer-2-Report-(Revision-1).pdf

Reviewer-2_Original-Submission-(Attachment).pdf

supporting information 1-for 3rd submission.pdf

supporting information 2-for 3rd submission.xlsx
