# Supplementary material for: Long-read sequencing of the coffee bean transcriptome reveals the diversity of full-length transcripts
Source: Gigascience. 2017 Aug 30;6(11):1–13. doi: 10.1093/gigascience/gix086 (PMC5737654; doi:10.1093/gigascience/gix086)

Manuscript Click here to download Manuscript Final (gigascience).docx

# Long-read sequencing of the coffee bean transcriptome reveals the diversity of full length transcripts

Bing Cheng, Agnelo Furtado, Robert J. Henry[1]

Queensland Alliance for Agriculture and Food Innovation, The University of Queensland, St Lucia, QLD 4072, Australia

[1] Corresponding author, Email: robert.henry@uq.edu.au

## Abstract

**Background**: Polyploidization contributes to the complexity of gene expression resulting in numerous related but different transcripts. This study explored the transcriptome complexity in tetraploid Arabica coffee (*Coffea arabica*) bean. Long-read sequencing (LRS) by Pacbio Isoform sequencing (Iso-seq) was used to obtain full length transcripts without the difficulty and uncertain of assembly required in short read technologies. The tetraploid transcriptome was annotated and compared with data from the diploid progenitors. Caffeine and sucrose genes were targeted for detailed analysis.

**Results**: A coffee bean transcriptome with 96,415 distinct transcripts (average 3,241 bp) was obtained. Of these, 1,271 were novel genes. The open reading frams (ORFs) predicted from 581 long transcripts (>10kb) were poorly annotated due to their novelty. With use of the LRS technique, longer UTRs were captured, especially in the 5'UTRs, facilitating the identification of upstream ORFs (uORFs). The LRS also revealed more and longer transcript variants in key caffeine and sucrose metabolism genes from this polyploid genome.

**Conclusion**: LRS technology shows the limitation of previous studies. It provides an important toolto produce a reference transcriptome including more of the diversity of full length transcripts to help understand the biology and support genetic improvement of polyploid species such as coffee.

**Key words**: coffee, transcriptome, full length, long sequences, isoform, polyploid, UTR

## Background

Polyploidy creates a complicated transcriptome with diverse transcript isoforms. As an important evolutionary process in plants, polyploidization generates new species and increases biodiversity. A balance of genetic and biochemical features is required for the polyploid to survive while carrying multiple genomes in the same nucleus [1]. Genetic changes associated with the formation of polyploids include gene function, which may remain unchanged, or diversify between the multiple homologues. Diversification or specialisation may alter the nature of the gene product (e.g. encoded protein sequence) or the pattern of expression (e.g. tissue specificity of expression) of genes from each subgenome. Moreover, the copy number of genes in each sub-genome may be altered or the gene may even be deleted completely from some sub-genomes [2]. Alternative splicing and polyadenylation also contribute further to the diversity of transcripts [3, 4]. Additionally, different 5'UTR accounts for different isoform variation, however, limited information is available on this for most genes. .This diversity may include different functional motifs, like upstream open reading frames, or introns harboured in this area, influencing post-transcription expression [5, 6]. All these influences contribute to a very complex transcriptome in polyploids.

The transcriptome represents all the genes expressed in the cell or tissue. RNA sequencing (RNA-Seq) makes it possible to capture the identity of these genes. Generating a reference transcriptome is essential for studying variation in expression of genes and the influence of genotype or environment on their expression [7, 8]. Most studies generate a reference transcriptome by short-read sequencing and reconstruct the transcriptome by assembly and/or mapping reads to other available reference genomes [9-11]. However, this is difficult for long transcripts, repetitive sequences and transposble elements. It is particularly challenging for complex polyploid genomes [12]. Long read sequencing (LRS) technology (eg PacBio) has

recently become available and this technology overcomes these difficulties by generating sequence information for the full length as a single sequence-read, including very long transcripts (eg. those exceeding 10kb) without the need for further assembly. This technique has been applied in a few plant studies and provides further information on transcript diversity, including alternative splicing and alternative polyadenylation [3, 4].

Arabica coffee is a recent allotetraploid (2n=4x=44; ~50,000 years old) derived from *C. canephora* and *C. eugenioides*. A high quality reference genome and annotation are not yet available for Arabica coffee.  However, a sequence is available for one of the sub-genomes, *C. canephora* [13]. Arabica coffee is highly regarded by coffee consumers, is of great economic value and accounts for almost 70% of world coffee traded (Fridell, 2014). However, it is produced in limited high altitude tropical environments and is threatened by climate change. Understanding the genetic and environmental control of coffee quality will be facilitated by the availability of a detailed knowledge of the transcriptome of the coffee bean. This study used LRS by Pacbio Iso-seq to characterise the Arabica coffee bean transcriptome including beans from immature, intermediate and mature stages in order to explore the complex polyploid system and establish a reference transcriptome for future studies of gene expression.

## Data Description

### *RNA sample preparation*

Fruits at different development stages (immature, intermediate and mature fruits) of *Coffea arabica* var. K7 (see Supporting Information 1 Fig. S1) were harvested from Green Cauldron Coffee, Federal, Australia. Ten coffee trees were selected randomly and 5 coffee fruits were collected separately for each tree and each stage of development. Samples were collected in triplicate. In total, 450 coffee fruits (900 beans) from 45 trees were collected. Once each fruit

was harvested, pericarp was removed immediately with a scalpel in 20 s or less. The coffee beans were immediately frozen in liquid nitrogen, transported on dry ice and stored at -80 $^{o}$C until further use. Total RNA was extracted from coffee fruits as described by Furtado [14] . Isolated RNA was assessed for integrity using a Agilent RNA 6000 nano kit and chips on a Bioanalyzer 2100 (Agilent Technologies, California, USA) and processed further for cDNA preparation.

*cDNA preparation*

The Pacbio Iso-seq protocol was used for cDNA preparation. cDNA was synthesised using Clontech SMARTer PCR cDNA Synthesis kit (ClonTech, Takara Bio Inc., Shiga, Japan) and amplified using a KAPA HIFI PCR kit (Kapa Biosystems, Boston, USA). The double stranded cDNA was split into two sub-samples. One was used directly for sequencing. The other set was normalised to equalise transcript abundance and obtain rare sequences.

The cDNA was purified for normalisation using a QIAquick PCR Purification Kit (Qiagen). The purified cDNA was precipitated and normalised with a Trimmer-2 cDNA normalisation kit (Evrogen, Moscow, Russia). The resulting cDNA was evaluated and quantified using a Agilent DNA 12000 Kit and Chips on a Bioanalyzer 2100 (Agilent Technologies, California, USA). The same amount of non-normalized and normalised cDNA was used as input for Pacbio Iso-seq.

Samples were subjected to Pacbio Iso-Seq protocol through purification, size selection (Blue Pippin system), re-amplification, SMRTbell template preparation and Iso-seq on a Pacbio RS II platform. A size selection protocol was applied as smaller cDNAs are more abundant and would otherwise be preferentially sequenced. Four bins were selected for non-normalized cDNA sequencing, with size ranges of 0.5-2.5kb, 2-3.5kb, 3-6.5kb and 5-10kb, respectively. Two bins were selected for normalised cDNA sequencing, 2-3.5kb and 3-6.5kb, as the normalisation biases against longer sequences.

*Raw read processing and error correction*

Sequence data was processed through the RS IsoSeq (version 2.3) pipeline. The first step was to remove adapters and artefacts to generate reads of insert (ROIs) consensus sequences. Non-Chimeric ROIs sequences were filtered into two groups of sequences comprised of full length ROIs sequences and non-full length ROIs sequences. Full length ROIs sequences were identified based on the presence of the 5'-adaptor sequence, the 3' adapter sequences (both used in the library preparation) and poly (A) tail. Further, FL ROIs sequences were passed through the isoform-level clustering (ICE) software module (https://github.com/PacificBiosciences/cDNA_primer/wiki/RS_IsoSeq-%28v2.3%29-Tutorial-%232.-Isoform-level-clustering-%28ICE-and-Quiver%29) to identify isoform sequences. Non full length ROI sequences were used to correct errors (polish) in the isoform sequences using the Quiver software module. The polishing process of Quiver generated two isoform sequence files, one with high quality (HQ) isoform sequences and the other with low quality (LQ) isoform sequences corresponding to an expected accuracy of ⩾ 99% or below respectively. LQ output (or non-FL coverage sequences) is useful in some cases, as it may result from rare transcripts or lower coverage sequences. And these low coverage sequences can be further used to correct errors in HQ output. The presence of Primer IIA sequence motifs (used in the library preparation) which escaped removal at the ROIs stage corresponded to 11 sequences were trimmed using CLC genomic workbench 9.0 (CLC). After combining the HQ and LQ transcripts, further clustering was processed with CD-HIT-EST (c=0.99) [15]. Chloroplast and mitochondrial transcript sequences in the clustered Iso-seq isoform sequences were identified for removal by mapping to the Arabica complete chloroplast genome (GenBank: EF044213.1) as well as *N. tabacum* and *V. vinifera* complete mitochondrial genomes (BA000042.1 and FM179380.1). This analysis was processed using CLC Genomic workbench 9.0 (QIAGEN, CLC Bio, Denmark). Additionally, filtered

sequences were subjected to BLAST (standalone version 2.3.0) and further filtered against *N.tabacum* ribosomal genes (5.8S-AJ012367, 18S-AJ236016 and 25S-S52185) under 1e-10. Sequence quality was then accessed with the Fasta Statistics through Galaxy/GVL 4.0 [16]. This set of Iso-seq processed isoforms was used for further analysis and named the "Coffee long read sequencing (coffee-LRS) isoforms" hereafter.

*Transcriptome annotation*

Different databases were applied for transcriptome annotation. The full set of the coffee-LRS isoform sequences were submitted to stand-alone BLAST against the NCBI non-redundant database (Aug 8$^{th}$, 2016, NCBI-nr) below 1e-5. After BLAST, data was processed further with the Blast2GO Pro 4.0 (North America, US: USA2 Version: b2g_Sep 16) pipeline based on default settings [17]. InterProScan (IPS) was used to search sequence protein domains from EBI databases to improve annotation. In the follow-up phase, Mapping, Annotation and Annex were applied to retrieve GO (gene ontology) terms, select reliable annotations and annotation augmentation respectively. The GO-slim tool was used against the plant database to provide plant generic GOs. Finally, GO enzyme mapping and KEGG (Kyoto encyclopaedia of genes and genomes) pathway maps were loaded. Caffeine and sucrose candidate genes were downloaded from the European Nucleotide Archive (EMBL-EBI) (Table 3 and Table 4) for specific analysis. For potential caffeine candidate genes, coffee-LRS isoform sequences were BLAST against reported caffeine genes. Transcripts with hits (potential caffeine isoforms) and those annotated by Blast2GO (potential sucrose transcripts) were further evaluated with Geneious 10.0.4 by aligning back to the candidate genes. Motif analysis was conductd with default parameters except 10 motifs with MEME 4.11.2 (http://meme-suite.org/tools/meme). UTRscan was used for UTR functional motifs annotation (http://itbtools.ba.itb.cnr.it/utrscan).

*Non-coding RNA*

The coffee-LRS isoform sequences without BLAST hits to NCBI-nr were scanned further with the *C. canephora* coding sequences (cds) (http://coffee-genome.org/sites/coffee-genome.org/files/download/coffea_cds.fna.gz) and Arabica coffee EST database [18] below 1e-10. Outputs without a hit were submitted to the Rfam database for non-coding RNA annotation from Blast2GO Pro pakage (North America, US: USA2 Version: b2g_Sep 16) for non-coding RNA analysis [19]. Sequences without a Rfam hit were processed to predict open reading frames (ORFs) more than 100 nucleotides long using getorf from EMBOSS package [16]. Long non-coding RNA (lncRNA) was predicted at a minimum of 350 nucleotides long.

*Analysis of long sequences*

In order to explore the advantage of using LRS platform to obtain long sequences, coffee-LRS isoform sequences longer than 10kb were selected and analysed. Selected isoforms were scanned with BLASTn against NCBI-nr (1e-5), followed by functional annotation using Blast2GO Pro (North America, US: USA2 Version: b2g_Sep 16) as above. Open reading frames (ORF) with a minimum 150 nucleotide length were predicted by EMBOSS on Galaxy/GVL 4.0 [16]. ORFs predicted were scanned with NCBI-nr databases.

## Analyses

*Overview of full Length RNA molecules from long-read sequencing*

A number of 2,618,905 raw reads were generated from LRS platform, which yielded 443,877 reads of insert. After 8,842 short sequences (less than 300 bp) were removed, 233,464 full length (FL) and 201,571 non full length (NFL) reads were generated. The average number of sequencing passes for these reads was 5. In total, 96,415 coffee-LRS isoform sequences were recovered after sequences representing chloroplast, mitochondrial and ribosomal transcripts

were removed. The length of the sequences in this dataset ranged from 301 bp to 23,335 bp, with an average length of 3,241 bp. The GC content was 41.4% and the N50 was 4,867 bp.

*Functional Annotation*

Functional annotation of the coffee-LRS isoform sequences was investigated using different databases. The data in Table 1 shows that 90,726 sequences (94.1%) corresponded to proteins from the NCBI non-redundant database (NCBI-nr). A total of 70,627 sequences (73.2%) matched to IPS protein domains. A number of 76,493 sequences (79.3%) had identified GOs. After the GOs were merged, 56,954 sequences (59.1%) were found to have reliable annotation and the same number matched with GO-slim (plant-slim).

When BLAST to NCBI-nr, the coffee-LRS isoform sequences had the highest number of hits to the *Nicotiana tabacum* (tobacco, 126,666 hits), followed by *C. canephora* (122,009 hits), *Vitis Vinifera* (grape, 79,386 hits) and *Sesamum indicum* (sesame, 68,388 hits) proteins. Of the 33,848 sequences (35.1%) with IPS GOs, cytochrome P450 (IPR001128, 357 matches) has the most sequence matches among the IPS families.

Biological process (BP) was more abundant than cellular component (CC) and molecular function (MF). Within these functional groups, the highest number of sequences were annotated with biosynthetic process (10,934 sequences, 11.3%), membrane component (18,244 sequences, 18.9%) and transferase activity (10,407 sequences, 10.8%), respectively. A number of 143 pathways were annotated by KEGG, associated with 7.0% of the whole dataset (6,775 sequences). Among these, starch and sucrose metabolism ranked as the fifth most abundant pathway, with 33 encoding enzymes and 601 isoforms annotated. Caffeine candidate genes were not identified by KEGG pathway analysis. To evaluate the annotated isoforms and their diversity, further analysis was performed with caffeine and sucrose pathways.

*Comparison to other available coffee databases*

To understand the diversity added to the transcriptome by polyploidy and this analysis, comparison was made between the Arabica EST database and the *C. canephora* cds. More than twice the number of isoforms were identified in the tetraploid Arabica LRS transcriptome compared with *C. eugenioides* contigs (36,935, from immature leaves and mature fruits) and *C. canephora* cds (25,574, from different tissues, including fruits) [13, 20]. Most sequences matched tobacco probably because the tobacco database is more extensive and well annotated than those of other related species, like *C. canephora*.

Results of BLAST analysis indicated that of the 96,415 coffee-LRS isoform sequences, 4,325 (4.48%) had no hits to the *C. canephora* coding sequences (cds) while 4,225 (4.38%) isoforms had no hits to the Arabica EST database. Conversely, 7,745 (30.28%) *C. canephora* coding sequences and 8,486 Arabica ESTs (24.14%) had hits to the coffee-LRS isoform sequences.

The coffee-LRS isoform sequences shows greater transcript length, diversity and a lower GC content (see Table 2). The N50 of the Pacbio dataset (4,867bp) was more than three times longer than that of the *C. canephora* cds and Arabica ESTs. The average length was more than twice that in the other databases with a maximum length up to 23,335 bp. Coffee-LRS isoform sequences are more diverse compare to either Arabica EST database or *C. canephora* cds.

*Isoform diversity in caffeine biosynthesis pathway*

The caffeine pathway has been widely studied previously (see Fig. 2a). Candidate genes and complete coding sequences of both transcripts and genomic DNA are available in public database and can be used as well-established references for caffeine candidate gene analysis (see Table 3). From the BLAST output, 26 long-read transcripts were annotated as different

candidate caffeine genes. Further alignment suggests 10 isoforms were likely to be putative caffeine genes, including 3 transcript variants of *XMT1*, 1 of *MXMT1*, 1 of *MXMT2* together with 2 of *DXMT1* and 3 of *DXMT2* genes (Fig. 2 and Table 3). The length distribution of these isoforms ranged between 977 and 1,517bp. All the 10 isoforms were extend at the 5' UTR region compared to the reported Arabica genes, while 8 isoforms were longer at the 3' end (Fig. 2b, 3c, 3f and Table 3). The most extended isoform (c695597/f1p2/1421) was 136 bp longer than the previously reported candidate genes (*CaXMT1*, Fig. 2b). Nine isoforms were found to be longer than the genomic DNA sequences in this study. The other isoform was likely to have resulted from an alternative polyadenylation event (c25904/f2p0/977, Fig. 2c) as two potential polyadenylation signals (AAUAAA) were identified in the 3' UTR (Fig 2d). Alternative splicing was also presents in caffeine isoforms, for example, intron retention was detected in one of the putative *DXMT2* isoforms (Fig. 2e).

*Long sucrose transcripts provides insight into complexity of polyploid system*

Sucrose genes were used to investigate the transcriptome sequence diversity of the polyploidy system. For the sucrose synthase 1 gene (*SS1)*, 9 transcript variants were identified (Fig. 3 and Table 4). Compared to c86432/f7p9/4842, the other 8 isoforms varied in motif replacement (motif 7 replace of motif 9 in c106591/f2p0/4381), deletion (for example c92344/f1p26/4662), relocation (intron retention, c92296/f1p5/4676 and c91298/f1p1/3137), etc. (Fig. 3b) The length of these putative *SS1* genes ranged from 2,961 to 4,842 bp. This is one of the important genes in sucrose gene family (Fig. 4a).

Importantly, all the sucrose candidate genes studied in this research were longer in the 5'UTR region than previously reported (Fig. 4b). For example, the longest putative *SS1* sequences identified, c86432/f2p7/4842 (4,842 bp), extended 2,131 bp upstream of the *C.canephora* coding sequence (*G-CcSS1*) and 1,994 bp of the Arabica sucrose synthase 1 mRNA sequence

(*CaSS1*). The length of the 5'leading region ranged between 218 and 2,131 bp (Table 5). To understand the diversity in this region, this set of sequence from the 9 putative *SS1* isoforms were scanned using the UTRdb online server. As many as 12 upstream open reading frames (uORFs) were identified and the number was positively correlated with length of the sequences. No uORFs were identified in two isoform, c62911/f29p21/2965 (218 bp leader sequence) and c72639/f25p28/2961 (232 bp leader sequence).

The 9 *SS1* isoforms revealed transcript diversity that resulted largely from different copies from the progenitors. When aligned to *G-CcSS1* (*C. canephora SS1* genomic sequence), the top 4 putative *SS1* isoforms showed high identity and consistent nucleotide variants (like the guanine highlighted at 3,726 bp in the consensus sequence, Fig. 4c), suggesting that these were copies from the *C. canephora* sub-genome. For example, compared to the consensus sequence, the same indels were present in 3,707bp and 3,733bp, cytosine at 3,713bp and guanine at 3,715bp, etc. One of these 5 isoforms, c92296/f1p5/4676, also had a higher number of variations where compared to *G-CcSS1*. Therefore, these 5 transcripts were likely to be *C. eugenioides* sub-genome derived copies. Consistently, intron retention present in one of the top 4 sequences, c91298/f1/p1/3137 (Fig. 4d) shows high homology to *C. canephora* intron 10 sequence. However, another one from the lower 5 transcripts had lots of compare to *C.canephora* intron 10, indicating this group is from a different copy, probably *C. eugenioides* (Fig. 4e). Additionally, some variants exist when compare to *G-CcSS1* but are common in 9 putative Arabica *SS1* isoforms and Arabica sucrose synthase 1(*CaSS1*), such as evidence shows in 3,666bp. This type of variants is probably result from different genotypes. Polyploid expression pattern was also observed in *SP1* transcript variants, the top 2 alignments were similar to *C.canephora* and the other 2 were slightly different but related. All of the 4 isoforms were longer in the upstream sequences while three extended further downstream than had previously been reported.

Another essential potential of LRS is to explore sequences not yet complete or published. For instance, 4 isoforms were identified from this research while SPS2 has only been identified in *C. canephora* ranther than *C. arabica* (Fig. 5).

*Novel genes*

After filtering LRS isoform sequences with NCBI-nr, 5,667 sequences without a hit were further filtered with *C.canephora* cds and Arabica EST databases resulting in 1,280 novel sequences. These sequences were submitted to the Rfam server to identify non-coding RNAs (ncRNA). Nine isoforms matched with known Rfam sequences, mainly in 5 biotypes, 3 transcripts were identified as CD-box snoRNA, 3 as HACA-box snoRNA, 2 as miRNA and the other one as tRNA.

Other than these, the other 1,271 sequences had no hit to the four databases and are likely to be novel genes that have not been discovered to date. Length distribution of this set of data was from 323 to 23,335bp. In total 5,431 ORFs were predicted, ranging between 102 and 1,179 bp. Of these, 145 ORFs were potentially long non-coding RNAs (LncRNAs).

*Long transcripts*

In order to discover the value of LRS in discovering long sequences, transcripts longer than 10 kb were further analysed. This identified 581 sequences, including 519 sequences (89.3 %) matching to the NCBI-nr, and 62 sequences without a hit that had identifiable ORFs. IPS matches were found for 293 sequences, 448 sequences were retrieved with GO terms, while 167 isoforms from these were also annotated with GO-Slim. Among the annotated isoforms, 22 sequences encoding 12 enzymes from 13 pathways were annotated with a KEGG pathway. Purine metabolism had the most sequences and enzymes annotated, followed by starch and sucrose metabolism. As most of the blast hits to NCBI-nr had less than 50% coverage, 8,934 ORFs (ranged from 150-5,229bp) were predicted to investigate

whether these long isoforms were real coding transcripts. However, only 757 ORFs (8.5 %) were annotated by NCBI-nr.

## Discussion

### *Polyploid expression*

Different isoforms may vary in function within the cell and be differentially expressed in tissues or environmental conditions. This study clearly shows the expression of sub-genome copies accounting for much of the polyploid diversity.

Generally, polyploidy results in three main expression patterns of non-additive expression [21], dominant expression in which total gene expression in the hybrid is similar to one of the parents, transgressive expression compared to the progenitors or unequal homeolog expression. Previously, it was proposed in coffee that lower caffeine in Arabica coffee was due to *C. eugenioides* sub-genome attributes [22]. *C. eugenioides* has very low caffeine biosynthesis together with a rapid catabolism [23]. Further studies are now possible based on the LRS isoforms identified in this study. First, it will be possible to determine whether the isoforms identified from the subgenome transcripts expressed in coffee follow a non-additive expression pattern. Moreover, it will be possible to determine whether this expression pattern is influenced by environment. Arabica is believed to be more adaptive to temperature change than its diploid parents [24]. It may also be possible to elucidate the genetic basis of the higher sucrose in Arabica coffee. More generally, the complete polyploid transcriptome from this study will improve understanding of the evolutionary adaptation and plasticity of polyploid species.

### *5'UTR extension*

All the caffeine and sucrose isoforms annotated in this study were extended in the 5'UTR compared to those available from public databases. Previously, it is difficult to sequence the

5’ end as cDNA library preparation starting from 3’ end normally fails to reach the 5’end. Further, it is not easy to assemble the non-coding parts of transcripts as limited protein sequence is available to guide assembly and confirm sequence. Therefore, less information is available in the 5’UTR, especially for plants. Generally, the length of the 5’UTR ranges from 100 up to few thousand bp [25]. This length difference is proposed because of the complex gene regulation maintained in eukaryotes [26]. Few post-transcriptional mechanisms have been studied in 5’UTRs, including the regulation by the pre-initiation complex and uORF re-initiation.

uORFs are common in 5’UTRs that have critical regulation. They contain their own set of start and stop codons that can be scanned by ribosomes and translated.This regulation can inhibit translation of the main ORF translation and reduce the amount of protein translated. Re-initiation of uORF regulation translation was found to be associated with the length of sequence between the uORF and the main ORF, suggesting interactions with translation factors required before initiation of translation [27]. This was also shown to be influenced by stress conditions [27]. However, not all uORF may have a role in translation control. In the leucine zipper transcription factor (*bZIP*) 11 gene, for example, harbouring four uORFs, only uORF2 was required for this regulation and this uORF is relatively conserved [28]. Other types of 5’UTR regulation may also found such as that due to introns in 5’UTRs. This happens to approximately 35% of human genes [5].

Understanding the mechanism of 5’UTR regulation will be greatly facilitated by the use of the full length transcripts. In this study, multiple uORFs were characterised in the *SS1* 5’ UTR and these may contribute to diverse functions and regulation that may be influenced by stress conditions. However, to confirm this, further phenotype, proteome and metabolome studies are required.

*Long transcripts*

LRS also has potential in discovering long gene isoforms, such as the sucrose synthases annotated here. Even though numerous studies have defined the sucrose pathways, not all the candidate genes had been identified. Many sucrose metabolism genes are too long to be captured by short read sequencing without significant *de novo* assembly. For example, the *C. arabica SS2* coding sequence is 2,889 bp and the genomic DNA sequence (exon 1 to 15) is 5,672 bp (Table 4). Sucrose synthase gens (6-7 different isoforms) were previously identified in cotton, rice, and Arabidopsis, However, in coffee, only 2 had been reported [29-31]. For genes that were only previously available for *C.canephora*, (eg *SP1*), this study also identified isoforms in Arabica. For genes that previously only had partial sequences available, (eg *SPS2*), the transcripts identified in this study will guide further studies and improve current databases.  Furthermore, the low level of annotation of long sequences (>10kb) ORFs by BLASTn against NCBI-nr confirms the limitations of current databases.

*Transcriptome analysis of polyploids using long-read sequencing*

LRS technologies shows advantages in understanding complex transcriptomes, especially from polyploid species [3, 32, 33]. First, this eliminates transcriptome reconstruction and that reduces the computation time. This is an essential goal for bioinformatics data analysis and software development [34]. To avoid obsolescence, transcriptome analysis calls for rapid genomics and bioinformatics to reduce the time from experiment to publication. Secondly, as there is no assembly of reads with LRS, there are no erroneous results due to mis-assemblies caused by complex polyploid transcriptomes with a large number of repeats or homologous genes. For example, almost 80% of the wheat genome is repetitive [33]. Last but not least, it shows the potential to capture rare or long sequences to provide an overview of the transcriptome and fully characterise RNA diversity, like 5'UTR extension in this study, alternative splicing, polyadenylation, etc. [3, 35].

Previous released PacBio single molecule real-time sequencing (SMRT) reads have a very high error rate, 11-14%, therefore, numerous methods have been proposed to correct the sequences [36]. One common approach was to map back to a reference genome and (or) use hybrid sequencing, for example, using short reads with high throughput to correct LRS isoform sequences [4, 37]. However, caution is necessary for using this strategy. The reference genome is often far from 100% accurate: 1) most draft genomes have numerous fragmented contigs or scaffolds with huge imbedded gaps. Even genomes previously considered well assembled have had many have many gaps[38]. 2) Problems also exist in poorly assembled gene loci. Very few recently released genomes have been re-visited to generate improved assemblies [12]. 3) LRS isoform sequences normally come from different sources (eg genotype) to the reference genomes that they can be compared with. Hybrid sequencing correction may have system bias and result in loss of isoforms or generating a "compromised" consensus. Previously, it has been estimated that there was no approach that has achieved more than 60 % accuracy for transcript reconstruction, even for the most studied human genome [39]. For instance, short read platforms deliver data that is less representative of rare or long isoforms and there is a high chance of losing these reads from long-read dataset when correcting.

The LRS isoform sequences strategy generates improved accuracy from CCS reads. This allows multiple passes of each transcript. Each pass can be used to correct the others with their random errors (mainly indels). The isoform clustering and polishing in this protocol is expected to deliver 99% accuracy. Prior to size selection, normalisation was further applied in parallel to the dataset in this study to decrease the frequency of abundant reads and produce a more even representation of the transcriptome and to capture rare sequences. A highly diverse transcriptome has resulted. The abundance of novel genes (1,271) and low annotation level of long sequences (8.5%) indicates the limits of previous studies.

In conclusion, this study will improve the understanding of the biology and genetic improvement of polyploid species such as coffee. It provides a useful technique to generate a full length reference transcriptome and improve understanding of UTR regions.

## Additional information

New sequence data used in this manuscript has been submitted to European Nucleotide Archive at EMBL database with accession number: PRJEB19262.

## Completing interests

All authors have no conflicts of interest to this manuscript.

## Funding

This study was funded by Australian Research Council (PROJECT ID: LP130100376) and Chinese Scholarship Council (2014-2018).

## Author's contributions

B.C., A.F. and R.H. designed the research and discussed the results. B.C performed the experiment and analysis. B.C drafted the manuscript, R.H and AG refined it.

## Acknowledgements

We thank Green Cauldron Coffee (Australia) for providing coffee materials, Prathima Perumal Thirugnanasambandam for assistance in sucrose synthase analysis, Kevin Smith for help in the informatics pipeline and the Research Computing Center of the University of Queensland, Australia for access to high performance computers. We also appreciated the help from Poss Reading, Marta Brozynska, Adam Healey, Tiparat Tikapunya, Ravi Nirmal, Nam Hoang and Hayba Badro in coffee sampling.

# Reference


1. Adams, K.L., et al., *Genes duplicated by polyploidy show unequal contributions to the transcriptome and organ-specific reciprocal silencing.* Proceedings of the National Academy of sciences, 2003. 100(8): p. 4649-4654.
2. Ha, M., E.-D. Kim, and Z.J. Chen, *Duplicate genes increase expression diversity in closely related species and allopolyploids.* Proceedings of the National Academy of Sciences, 2009. 106(7): p. 2295-2300.
3. Wang, B., et al., *Unveiling the complexity of the maize transcriptome by single-molecule long-read sequencing.* Nature Communications, 2016. 7.
4. Abdel-Ghany, S.E., et al., *A survey of the sorghum transcriptome using single-molecule long reads.* Nature Communications, 2016. 7.
5. Bicknell, A.A., et al., *Introns in UTRs: why we should stop ignoring them.* Bioessays, 2012. 34(12): p. 1025-1034.
6. Mignone, F., et al., *Untranslated regions of mRNAs.* Genome biology, 2002. 3(3): p. reviews0004. 1.
7. Van Veen, H., et al., *Transcriptomes of eight Arabidopsis thaliana accessions reveal core conserved, genotype-and organ-specific responses to flooding stress.* Plant physiology, 2016: p. pp. 00472.2016.
8. Garg, R., et al., *Transcriptome analyses reveal genotype-and developmental stage-specific molecular responses to drought and salinity stresses in chickpea.* Scientific reports, 2016. 6.
9. Grabherr, M.G., et al., *Full-length transcriptome assembly from RNA-Seq data without a reference genome.* Nature biotechnology, 2011. 29(7): p. 644-652.
10. Wang, X.-W., et al., *De novo characterization of a whitefly transcriptome and analysis of its gene expression during development.* BMC genomics, 2010. 11(1): p. 400.
11. Li, P., et al., *The developmental dynamics of the maize leaf transcriptome.* Nature genetics, 2010. 42(12): p. 1060-1067.
12. Michael, T.P. and R. VanBuren, *Progress, challenges and the future of crop genomes.* Current opinion in plant biology, 2015. 24: p. 71-81.
13. Denoeud, F., et al., *The coffee genome provides insight into the convergent evolution of caffeine biosynthesis.* science, 2014. 345(6201): p. 1181-1184.
14. Furtado, A., *RNA Extraction from Developing or Mature Wheat Seeds.* Cereal Genomics: Methods and Protocols, 2014: p. 23-28.
15. Fu, L., et al., *CD-HIT: accelerated for clustering the next-generation sequencing data.* Bioinformatics, 2012. 28(23): p. 3150-3152.
16. Afgan, E., et al., *Genomics Virtual Laboratory: a practical bioinformatics workbench for the cloud.* PloS one, 2015. 10(10): p. e0140829.
17. Götz, S., et al., *High-throughput functional annotation and data mining with the Blast2GO suite.* Nucleic acids research, 2008. 36(10): p. 3420-3435.
18. Mondego, J.M., et al., *An EST-based analysis identifies new genes and reveals distinctive gene expression features of Coffea arabica and Coffea canephora.* BMC plant biology, 2011. 11(1): p. 1.
19. Nawrocki, E.P., et al., *Rfam 12.0: updates to the RNA families database.* Nucleic acids research, 2014: p. gku1063.
20. Yuyama, P.M., et al., *Transcriptome analysis in Coffea eugenioides, an Arabica coffee ancestor, reveals differentially expressed genes in leaves and fruits.* Molecular Genetics and Genomics, 2016. 291(1): p. 323-336.
21. Yoo, M.-J., et al., *Nonadditive gene expression in polyploids.* Annual review of genetics, 2014. 48: p. 485-517.
22. Perrois, C., et al., *Differential regulation of caffeine metabolism in Coffea arabica (Arabica) and Coffea canephora (Robusta).* Planta, 2015. 241(1): p. 179-191.

23. Ashihara, H. and A. Crozier, *Biosynthesis and catabolism of caffeine in low-caffeine-containing species of Coffea.* Journal of agricultural and food chemistry, 1999. 47(8): p. 3425-3431.
24. Combes, M.C., et al., *Contribution of subgenomes to the transcriptome and their intertwined regulation in the allopolyploid Coffea arabica grown at contrasted temperatures.* New phytologist, 2013. 200(1): p. 251-260.
25. Lodish, H., *Molecular cell biology*. 2008: Macmillan.
26. Rhind, N., et al., *Comparative functional genomics of the fission yeasts.* Science, 2011. 332(6032): p. 930-936.
27. Somers, J., T. Pöyry, and A.E. Willis, *A perspective on mammalian upstream open reading frame function.* The international journal of biochemistry & cell biology, 2013. 45(8): p. 1690-1700.
28. Hummel, M., et al., *Sucrose-mediated translational control.* Annals of botany, 2009: p. mcp086.
29. Chen, A., et al., *Analyses of the sucrose synthase gene family in cotton: structure, phylogeny and expression patterns.* BMC plant biology, 2012. 12(1): p. 85.
30. Hirose, T., G.N. Scofield, and T. Terao, *An expression analysis profile for the entire sucrose synthase gene family in rice.* Plant Science, 2008. 174(5): p. 534-543.
31. Bieniawska, Z., et al., *Analysis of the sucrose synthase gene family in Arabidopsis.* The Plant Journal, 2007. 49(5): p. 810-828.
32. Minoche, A.E., et al., *Exploiting single-molecule transcript sequencing for eukaryotic gene prediction.* Genome biology, 2015. 16(1): p. 1.
33. Dong, L., et al., *Single-molecule real-time transcript sequencing facilitates common wheat genome annotation and grain transcriptome research.* BMC genomics, 2015. 16(1): p. 1039.
34. Haas, B.J., et al., *De novo transcript sequence reconstruction from RNA-seq using the Trinity platform for reference generation and analysis.* Nature protocols, 2013. 8(8): p. 1494-1512.
35. Gonzalez-Garay, M.L., *Introduction to isoform sequencing using pacific biosciences technology (Iso-Seq)*, in *Transcriptomics and Gene Regulation*. 2016, Springer. p. 141-160.
36. Roberts, R.J., M.O. Carneiro, and M.C. Schatz, *The advantages of SMRT sequencing.* Genome biology, 2013. 14(7): p. 1.
37. Xu, Z., et al., *Full‐length transcriptome sequences and splice variants obtained by a combination of sequencing platforms applied to different root tissues of Salvia miltiorrhiza and tanshinone biosynthesis.* The Plant Journal, 2015. 82(6): p. 951-961.
38. Lamesch, P., et al., *The Arabidopsis Information Resource (TAIR): improved gene annotation and new tools.* Nucleic acids research, 2012. 40(D1): p. D1202-D1210.
39. Korf, I., *Genomics: the state of the art in RNA-seq analysis.* Nature methods, 2013. 10(12): p. 1165-1166.
40. Cheng, B., et al., *Influence of genotype and environment on coffee quality.* Trends in Food Science & Technology, 2016.

**Tables and figure legends**

Table 1 Arabica long-read sequencing transcriptome annotation with different databases

| Databases | Number of sequences annotated | % of sequences annotated |
|---|---|---|
| Long-read sequencing transcriptome | 96, 415 | - |
| NCBI non-redundant | 90,726 | 94.1% |
| Mapping | 76,493 | 79.3% |
| InterProScan | 70,613 | 73.2% |
| InterProScan GOs | 33,848 | 35.1% |
| Annotation | 56,954 | 59.1% |
| GO slim | 56,954 | 59.1% |
| KEGG | 6,775 | 7.0% |

Table 2 Arabica long-read sequencing isoforms compared to *Coffea canphora* coding sequences and *Coffea arabica* EST sequences

| Different datasets | GC content % | N50 (bp) | average length (bp) | min length (bp) | max_length (bp) | Number of sequences |
|---|---|---|---|---|---|---|
| *Coffea arabica* EST database [1] [18] | 44.7 | 734 | 662 | 32 | 3,584 | 35,153 |
| *Coffea canphora* coding sequences [2] | 43.9 | 1,587 | 1,205 | 45 | 16,431 | 25,574 |
| ***Coffea arabica* long-read sequencing isoforms** | **41.4** | **4,867** | **3,241** | **301** | **23,335** | **96,415** |

Note: [1] http://bioinfo03.ibi.unicamp.br/coffea/data/CA.fasta; [2] http://coffee-genome.org/sites/coffee-genome.org/files/download/coffea_cds.fna.gz.

Table 3 Details of caffeine candidate genes, putative transcript variants annotated and 5‘UTR extension information

| Caffeine andidate genes | Acession number | Species | Source | Abbreviation | length (bp) | completeness | Putative transcript variants from LRS isoform sequences | 5'UTR extension |
|---|---|---|---|---|---|---|---|---|
| xanthosine methyltransferase 1 | AB048793 | *C. arabica* | mRNA | CaXMT1 | 1,316 | YES | c69597/f1p2/1421<br>c154338/f1p2/1360<br>c71416/f3p3/1376 | YES |
| | JX978514 | *C. arabica* | Genomic DNA | G-CaXMT1 | 1,987 | YES | | |
| | DQ422954 | *C. canephora* | mRNA | CcXMT1 | 1,316 | YES | | |
| | JX978509 | *C. canephora* | Genomic DNA | G-CcXMT1 | 1,994 | YES | | |
| xanthosine methyltransferase2 | JX978515 | *C. arabica* | Genomic DNA | G-CaXMT2 | 2,038 | YES | Not identified | - |
| 7-methylxanthine N-methyltransferase 1 | AB048794 | *C. arabica* | mRNA | CaMXMT1 | 1,298 | YES | c20397/f5p1/1361 | YES |
| | JX978511 | *C. arabica* | Genomic DNA | G-CaMXMT1 | 1,838 | YES | | |
| | HQ616707 | *C. canephora* | mRNA | CcMXMT1 | 1,222 | YES | | |
| | JX978507 | *C. canephora* | Genomic DNA | G-CcMXMT1 | 1,829 | YES | | |
| 7-methylxanthine N-methyltransferase 2 | AB084126 | *C. arabica* | mRNA | CaMXMT2 | 1,155 | YES | c10402/f2p3/1277 | YES |
| | JX978512 | *C. arabica* | Genomic DNA | G-CaMXMT2 | 2,010 | YES | | |
| 3,7-dimethylxanthine N- | AB084125 | *C. arabica* | mRNA | CaDXMT1 | 1,155 | YES | c25904/f2p0/977<br>c71881/f6p2/1386 | YES |
| | JX978510 | *C. arabica* | Genomic DNA | G-CaDXMT1 | 2,063 | YES | | |
| | DQ422955 | *C. canephora* | mRNA | CcDXMT1 | 1,364 | YES | | |

| | | | | | | | | |
|---|---|---|---|---|---|---|---|---|
| methyltransferase 1 | | | | | | | | |
| 3,7-dimethylxanthine N-methyltransferase 2 | KJ577793 | *C. arabica* | mRNA | CaDXMT2 | 1,155 | YES | c63815/f1p2/1273 c48759/f1p1/1517 c26870/f6p5/1402 | YES |
| | KJ577792 | *C. arabica* | Genomic DNA | G-CaDXMT2 | 2,006 | YES | | |

Table 4 Details of sucrose candidate genes, putative transcript variants annotated and 5‘UTR extension information

| Candidate genes | Acession number | Species | Source | Abbreviation | length (bp) | completeness | Putative transcript variants from LRS isoform sequences | 5'UTR extension |
|---|---|---|---|---|---|---|---|---|
| Sucrose synthase 1 | AM087674.1 | *C. arabica* | mRNA | CaSS1 | 2,979 | YES | c86432/f7p9/4842<br>c91298/f1p1/3137<br>c84406/f3p18/2975<br>c62911/f29p21/2965<br>c92344/f1p26/4662<br>c92296/f1p5/4676<br>c89510/f1p6/4592<br>c106591/f2p0/4381<br>c72639/f25p28/2961 | YES |
| | DQ834312.1 | *C. canephora* | mRNA | CcSS2 | 2,989 | YES | | |
| | AJ880768.2 | *C. canephora* | Genomic DNA | G-CcSS1 | 3,957 | exon 1-13 | | |
| Sucrose synthase 2 | AM087675.1 | *C. arabica* | mRNA | CaSS2 | 2,889 | YES | c73322/f3p2/3080<br>c75363/f3p2/2906 | YES |
| | AM087676.1 | *C. canephora* | Genomic DNA | G-CcSS2 | 5,672 | exon 1-15 | | |
| Sucrose phosphate synthase 1 | DQ834321.1 | *C. canephora* | mRNA | CcSPS1 | 3,150 | YES | c51110/f2p0/3136 | YES |
| | DQ842233.1 | *C. canephora* | Genomic DNA | G-CcSPS1 | 8,215 | YES | | |
| Sucrose phosphate synthase 2 | DQ842234.1 | *C. canephora* | Genomic DNA | G-CcSPS2 | 1,550 | NO | c103631/f1p2/4695<br>c88660/f2p0/4282<br>c106342/f1p4/4274<br>c104672/f1p1/4440 (reverse) | YES |

Table 5 Results of 5’ UTRs from long-read sequencing scanned with UTRdb. uORF, Upstream Open Reading Frame.

| No. | Sequence name | 5' UTR length (bp) | uORF |
|---|---|---|---|
| 1 | c86432/f2p7/4842 | 2,131 | 12 |
| 2 | c91298/f1p1/3137 | 347 | 2 |
| 3 | c84406/f3p18/2975 | 242 | 2 |
| 4 | c62911/f29p21/2965 | 218 | 0 |
| 5 | c92344/f1p26/4662 | 1,981 | 10 |
| 6 | c92296/f1p5/4676 | 1,884 | 12 |
| 7 | c89510/f1p6/4592 | 1,871 | 11 |
| 8 | c106591/f2p0/4381 | 1,683 | 11 |
| 9 | c72639/f25p28/2961 | 224 | 0 |

Figure 1 Coffee fruits of immature, intermediate and mature stages

Figure 2 Putative transcript variants from long-read sequencing aligned to reference caffeine genes. a. Main caffeine biosynthesis pathway in coffee, adaptive from Cheng, Furtado [40]. b. Alignment of three Arabica putative XMT1 variants from long-read sequencing (c69597/f1p2/1412, c154338/f1p2/1360 and c71416/f3p3/1376), Coffea arabica and *Coffea canphora* XMT1 (CaXMT1 and CcXMT1) to Arabica XMT1 genomic DNA sequence (G-CaXMT1). c. Possible alternative polyadenylation of putative XMT1 Iso-seq variant (c25904/f2p0977) from long-read sequencing; G-CaDXMT1, Arabica DXMT1 genomic DNA sequence; CaDXMT1, DXMT1 coding sequence; d. Two polyadenylation signals were identified in 3’ends of c25904/f2p0/977; e. Possible alternative splicing (intron retention) in one of the putative DXMT2 variants (c48759/f1p1/1517) from long-read sequencing transcripts; G-CaDXMT2, Arabica DXMT2 genomic DNA sequence; CaDXMT2, Arabica DXMT2 coding sequence. (Note: black colour in the alignment means different nucleotides to reference sequence, Arabica genomic XMT1, while grey colour means the same nucleotides). Different nucleotides compared to consensus were highlighted in black in the alignment, except consensus sequences.

Figure 3 Motif search results of putative sucrose synthase gene 1 from long read sequencing. a. Ten motifs were annotated in 9 putative sucrose synthase 1 variants from long-read sequencing, analysed by MEME 4.11.2. b. Motif location of 9 putative sucrose synthase 1 variants. Different motifs were highlighted with red arrows and intron retention was shown with dashed boxs.

Figure 4 Putative variants from long-read sequencing aligned to reference sucrose genes. a. Possible sucrose metabolism in coffee; SS, sucrose synthase; SPS, sucrose phosphate synthase; SP, sucrose phosphatase; INV, invertase; CINV, cell wall invertase (modified from Cheng B. et al. (2016)); b. Alignment of 9 Putative Sucrose synthase variants from long-read sequencing and *C.arabica* sucrose synthase gene 1 (CaSS1) to *Coffea canphora* genomic sucrose synthase 1 (exons 1-13) (G-CcSS1 (1-13)); Green box highlights variants result from different copies of subgenomes, while intron retention events were cited with blue box highlight; c. polyploid expression when zooming green area in 100%; d. possible alternative

splicing (intron retention) from a *C.canephora* subgenome copy when zooming blue box in 100%; e. possible intron retention from a *C.eugenioides* subgenome copy when zoom blue area in 100%.red line classifies two groups of variants as different copies of subgenomes. Different nucleotides compared to consensus were highlighted in black in the alignment, except consensus sequences.

Figure 5 Putative variants from long read sequencing aligned with *C.canephora* genomic sucrose phosphate synthase 2 sequence; FWD, forward sequence; REV, reverse sequence. Different nucleotides compared to consensus were highlighted in black in the alignment, except consensus sequences.

Figure 1

Click here to download Figure Fig 1.tif

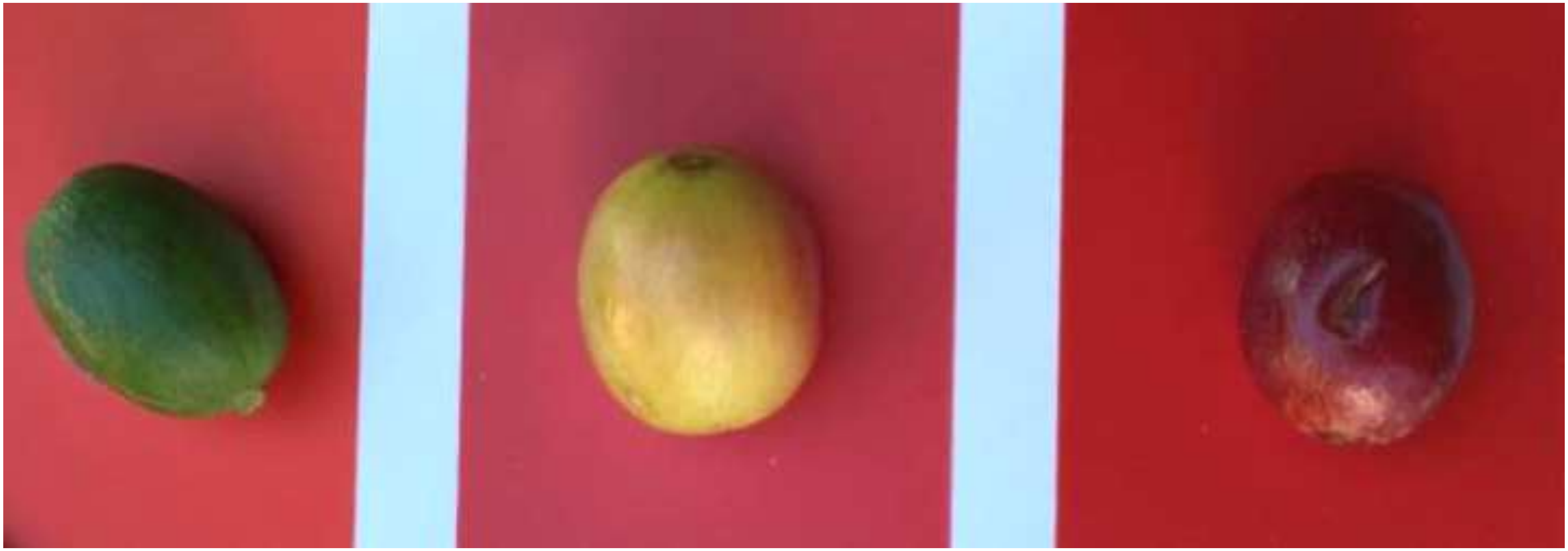

Figure 2

Click here to download Figure Fig 2.tif

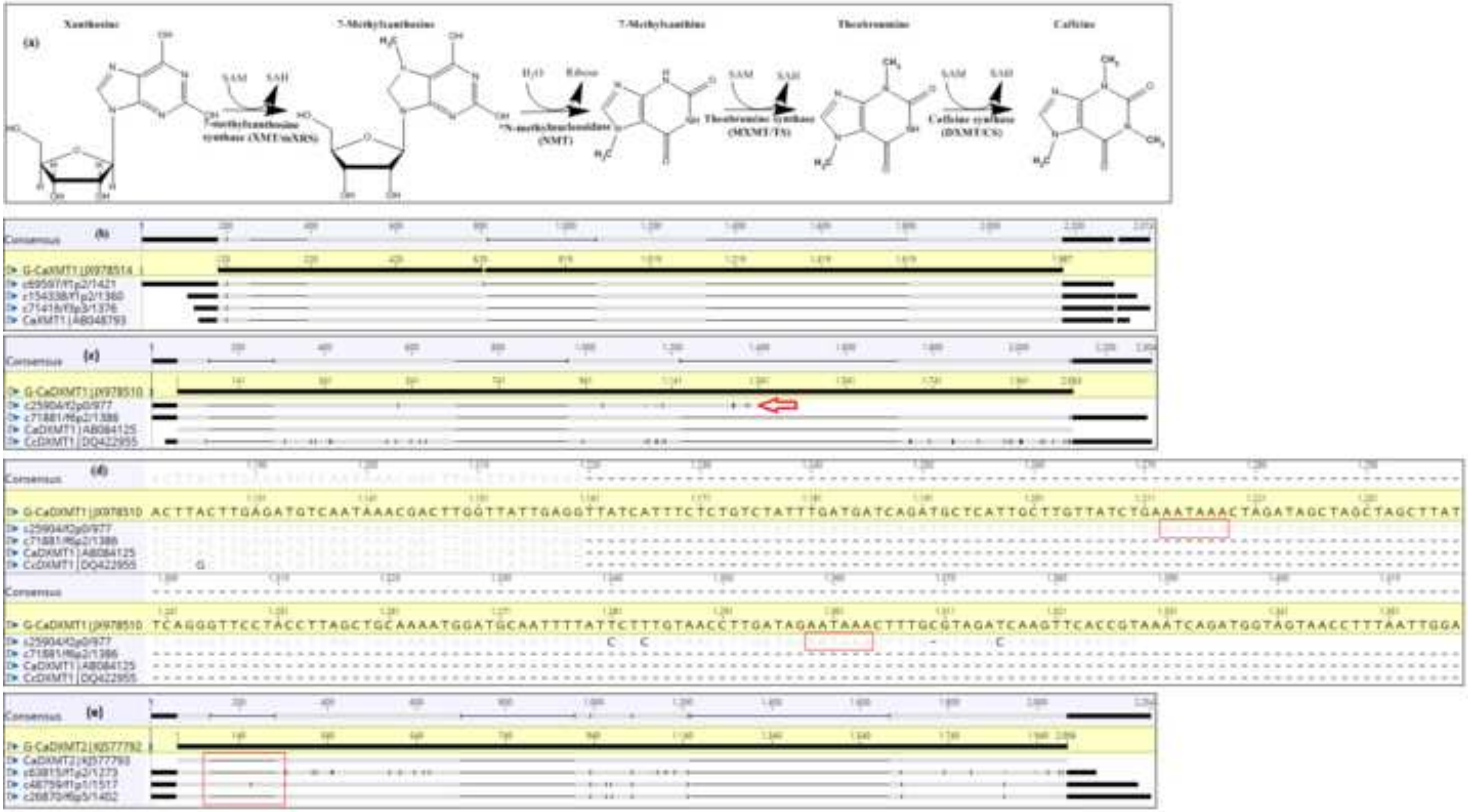

Figure 3

Click here to download Figure Fig 3.tif

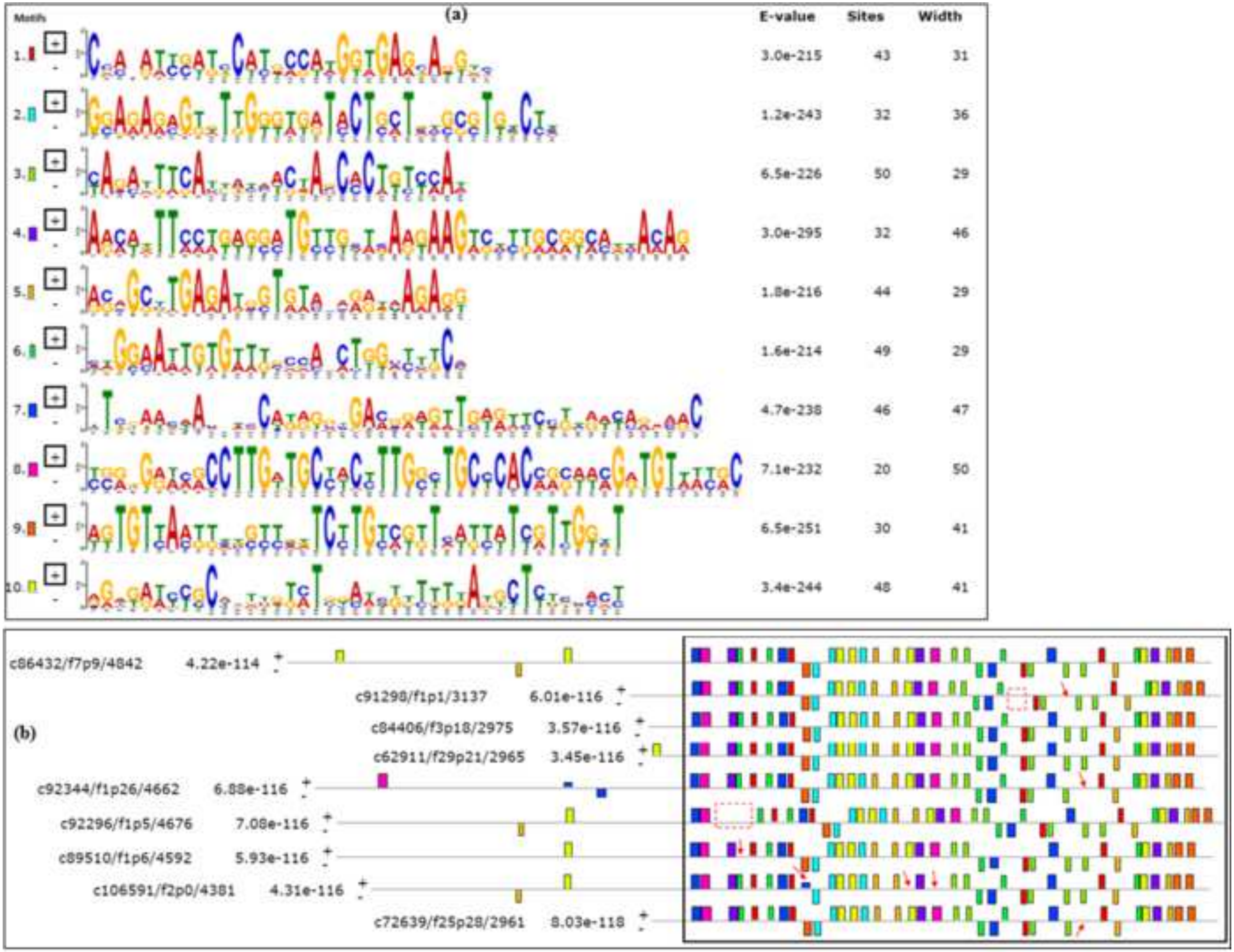

Figure 4

Click here to download Figure Fig 4.tif

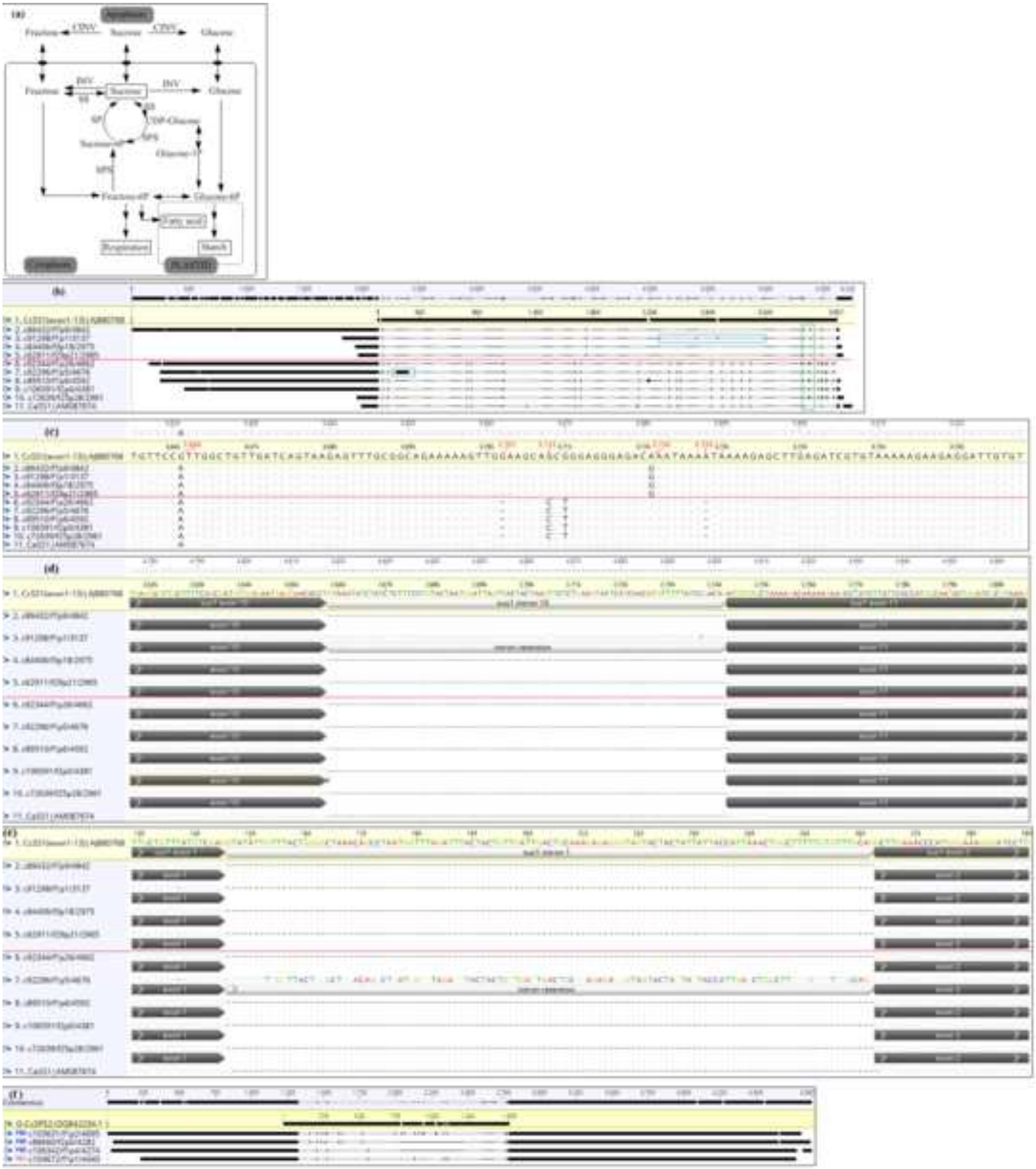

Figure 5

Click here to download Figure Fig 5.tif

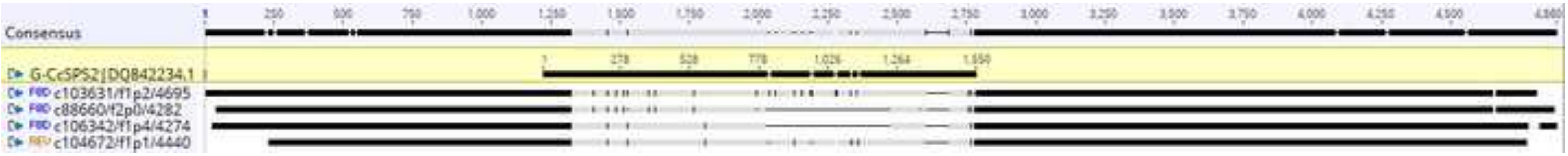

Supplement: GIGA-D-17-00024_Original-Submission.pdf [file gix086_giga-d-17-00024_original-submission.pdf]
