# Supplementary material for: Long-read sequencing of the coffee bean transcriptome reveals the diversity of full-length transcripts
Source: Gigascience. 2017 Aug 30;6(11):1–13. doi: 10.1093/gigascience/gix086 (PMC5737654; doi:10.1093/gigascience/gix086)
Supplement: GIGA-D-17-00024_Revision-1.pdf [file gix086_giga-d-17-00024_revision-1.pdf]

Manuscript Click here to download Manuscript Resubmission.docx

Long-read sequencing of the coffee bean transcriptome reveals the diversity of full-length transcripts

Bing Cheng, Agnelo Furtado, Robert J. Henry[1]

Queensland Alliance for Agriculture and Food Innovation, The University of Queensland, St Lucia, QLD 4072, Australia

Abstract

Background: Polyploidization contributes to the complexity of gene expression resulting in numerous related but different transcripts. This study explored the transcriptome diversity and complexity of tetraploid Arabica coffee (*Coffea arabica*) bean. Long-read sequencing (LRS) by Pacbio Isoform sequencing (Iso-seq) was used to obtain full-length transcripts without the difficulty and uncertainty of assembly required for reads from short read technologies. The tetraploid transcriptome was annotated and compared with data from the sub-genome progenitors. Caffeine and sucrose genes were targeted for case analysis.

Findings: An isoform-level tetraploid coffee bean reference transcriptome with 95,995 distinct transcripts (average 3,236 bp) was obtained. A total of 88,715 sequences (92.42%) were annotated with BLASTx against NCBI non-redundant plant proteins, including 34,719 high quality annotations. Further BLASTn to NCBI non-redundant nucleotide sequences, *C. canephora* coding sequences with UTR, C.arabica ESTs and Rfam resulted in 1,213 sequences without hits, were potential novel genes in coffee. Longer UTRs were captured, especially in the 5'UTRs, facilitating the identification of upstream ORFs (uORFs). The LRS also revealed more and longer transcript variants in key caffeine and sucrose metabolism genes from this polyploid genome. Long sequences (>10kb) were poorly annotated.

Conclusions: LRS technology shows the limitation of previous studies. It provides an important tool to produce a reference transcriptome including more of the diversity of full-length transcripts to help understand the biology and support the genetic improvement of polyploid species such as coffee.

Keywords: coffee, transcriptome, full-length cDNA, long sequences, isoform, polyploid, UTR

Background

Polyploidy creates a complicated transcriptome with diverse transcript isoforms. As an important evolutionary process in plants, polyploidization generates new species and increases biodiversity [1]. A balance of genetic and biochemical features is required for the polyploid to survive while carrying multiple genomes in the same nucleus [2]. Genetic changes associated with the formation of polyploids include gene function, which may remain unchanged, or diversification among the multiple homeologs, leading to neofunctionalization, subfunctionalization, or pseudogenization [3]. Alternative splicing and polyadenylation also contribute further to the diversity of transcripts [4, 5]. Additionally, different 5'UTRs account for transcript variation, however, limited information is available on this for most genes. This diversity may include different functional motifs, like upstream open reading frames, or introns harboured in this area, influencing post-transcription expression [6, 7]. All these influences contribute to the diversity and complexity of a polyploid transcriptome.

The transcriptome represents all the genes expressed in the cell or tissue. RNA sequencing (RNA-Seq) makes it possible to capture the identity of these genes. Generating a reference transcriptome is essential for studying variation in expression of genes and the influence of genotype or environment on their expression [8, 9]. Most studies generate a reference transcriptome by short-read sequencing and reconstruct the transcriptome by the assembly and/or mapping of reads to other available reference genomes [10-12]. However, this is difficult for long transcripts, repetitive sequences and transposable elements. It is particularly challenging for complex polyploid genomes [13]. The LRS technology (e.g. PacBio) has recently become available and this technology overcomes these difficulties by generating sequence information for the full length as a single sequence read, including very long transcripts (*e.g.* those exceeding 10kb) without the need for further assembly. This technique

has been applied in a few plant studies and provides further information on transcript diversity, including alternative splicing and alternative polyadenylation [4, 5].

Arabica coffee is a recent allotetraploid (2n=4x=44; ~50,000 years old) derived from *C. canephora* and *C. eugenioides*. A high-quality reference genome and annotation are not yet available for Arabica coffee. However, a draft genome is available for one of the sub-genomes, *C. canephora* [14]. Arabica coffee is highly regarded by coffee consumers, is of great economic value and accounts for almost 70% of world coffee traded [15] However, it is produced in limited high altitude tropical environments and is threatened by climate change. Understanding the genetic and environmental control of coffee quality will be facilitated by the availability of detailed knowledge of the transcriptome of the coffee bean. This study used LRS by Pacbio Iso-seq to characterise the Arabica coffee bean transcriptome including beans from immature, intermediate and mature stages in order to explore the complex polyploid system and establish a reference transcriptome for future studies of gene expression.

Data Description

RNA sample preparation

Fruits at different development stages (immature, intermediate and mature fruits) of *Coffea arabica* var. K7 (see Supporting Information 1 Fig. S1) were harvested from Green Cauldron Coffee, Federal, Australia. Ten coffee trees were selected randomly and 5 coffee fruits were collected separately for each tree and each stage of development. Samples were collected in triplicate. In total, 450 coffee fruits (900 beans) from 45 trees were collected. Once each fruit was harvested, the pericarp was removed immediately with a scalpel in 20 s or less. The coffee beans were immediately frozen in liquid nitrogen, transported on dry ice and stored at -80 ^{o}C until further use. Total RNA was extracted from coffee fruits as described by Furtado

[16]. Isolated RNA was assessed for integrity using an Agilent RNA 6000 nano kit and chips on a Bioanalyzer 2100 (Agilent Technologies, California, USA) and processed further for cDNA preparation.

cDNA preparation

The Pacbio Iso-seq protocol was used for cDNA preparation. cDNA was synthesised using a Clontech SMARTer PCR cDNA Synthesis kit (ClonTech, Takara Bio Inc., Shiga, Japan) and amplified using a KAPA HIFI PCR kit (Kapa Biosystems, Boston, USA). The double-stranded cDNA was split into two sub-samples. One was used directly for sequencing. The other set was normalised to equalise transcript abundance and obtain rare sequences.

The cDNA was purified for normalisation using a QIAquick PCR Purification Kit (Qiagen). The purified cDNA was precipitated and normalised with a Trimmer-2 cDNA normalisation kit (Evrogen, Moscow, Russia). The resulting cDNA was evaluated and quantified using an Agilent DNA 12000 Kit and Chips on a Bioanalyzer 2100 (Agilent Technologies, California, USA). The same amount of non-normalized and normalised cDNA was used as input for Pacbio Iso-seq.

Samples were subjected to a Pacbio Iso-Seq protocol through purification, size selection (Blue Pippin system), re-amplification, SMRTbell template preparation and Iso-seq on a Pacbio RS II platform. A size selection protocol was applied as smaller cDNAs are more abundant and would otherwise be preferentially sequenced. Four Bluepippin bins were selected for non-normalized cDNA sequencing, with size ranges of 0.5-2.5kb, 2-3.5kb, 3-6.5kb and 5-10kb, respectively since Pacbio sequencing preferentially sequences short DNA fragments. Two bins were selected for normalised cDNA sequencing, 2-3.5kb and 3-6.5kb, as the normalisation biases against longer sequences.

Raw read processing and error correction

Sequence data was processed through the RS IsoSeq (version 2.3) pipeline [17]. The first step was to remove adapters and artefacts to generate reads of insert (ROIs) consensus sequences. Short sequences less than 300 bp were removed as the Bluepippin cDNA size selection starts from 500 bp, where some sequences less than 500 bp have a chance to be sequenced. Non-Chimeric ROIs sequences were filtered into two groups of sequences comprised of full-length ROIs sequences and non-full length ROIs sequences. Full-length (FL) ROIs sequences were identified based on the presence of the 5'-adaptor sequence, the 3' adapter sequences (both used in the library preparation) and poly (A) tail. Further, FL ROIs sequences were passed through the isoform-level clustering (ICE). ROI sequences were used to correct errors (polish) the isoform sequences using the Quiver software module. The polishing process of Quiver generated two isoform sequence files, one with high quality (HQ) isoform sequences and the other with low quality (LQ) isoform sequences corresponding to an expected accuracy of $\geqslant$ 99% or below respectively. LQ output (or non-FL coverage sequences) is useful in some cases, as it may result from rare transcripts or lower coverage sequences. And these low coverage sequences can be further used to correct errors in HQ output. The Primer IIA sequence motifs (used in the library preparation) which escaped removal at the ROIs stage corresponded to 11 sequences were trimmed using CLC genomic workbench 9.0 (CLC, QIAGEN, CLC Bio, Denmark). After combining the HQ and LQ transcripts, further clustering was processed with CD-HIT-EST (c=0.99) [18].

In the following step, the contaminant sequences were removed by CLC stepwise as follows. 1) Chloroplast transcript sequences were identified by BLASTn to the *C.arabica* complete chloroplast genome (GenBank: EF044213.1). 2) Mitochondrial transcripts were characterised by BLASTn to *N. tabacum* and *V. vinifera* complete mitochondrial genomes (BA000042.1 and FM179380.1). 3) Ribosomal sequences were detected by BLASTn to the reported

C.arabica, *C.canephora* and *C. eugenioides* ribosomal genes (AJ224846, EU650386, DQ153609, AF416459, EU650384, EU650385, AF542981, AF542990, JX459583, JX459584, JX459585, JX459586, JX459587, DQ153593, AF542982, DQ423064, DQ153588, DQ153621, AF542986). 4) Virus, viroid and prokaryote contaminants were identified with BLASTn to their reference genomes from the NCBI database (April 4th, 2017). Prokaryotic contaminants were screened with available reference genomes from NCBI (Feb 9th, 2017). 5) Fungal sequences were investigated by BLASTn to fungal proteins (April 4th, 2017). All the above analyses were processed one after another with a maximum E-value threshold of 1e-10.

From the BLASTn results, significant matches were filtered with a bit score (A) ≥ 300 as well as identity ≥ 80%. In each step, the filtered significant sequences were processed further with cloud BLASTn to the NCBI non-redundant database (bit score: B) to further confirm the matches. This validation step was confirmed by comparison of the bit score (comparison of value A and B). If the higher bit score was associated with a contaminant sequence in the BLASTn (A>B), then the sequence was discarded. In total 526 sequences corresponding to chloroplastic (200), mitochondrial (264), ribosomal (37), viral (0), viroid (0), prokaryotic (0) and fungal (25) contaminant sequences, respectively, were removed in this process. Sequence quality was then accessed with the Fasta Statistics through Galaxy/GVL 4.0 [19]. This set of Iso-seq processed isoforms was used for further analysis and hereafter named the “Coffee long read sequencing (coffee-LRS) isoforms”. The term ‘isoforms’, or ‘isoform sequence’ or ‘transcript’ used in this study represent individual sequences from the coffee-LRS isoforms, while “transcript variants” indicate different transcript of a gene, including alternative spliced variants, homeologs, etc.

Transcriptome annotation

A number of databases were used for annotation of the coffee-LRS isoforms described as follows. 1) The plant Geninfo identifier (GI) list was downloaded from NCBI Protein Entrez (May 2nd, 2017, 8,431,379 items). The plant proteins were retrieved from the NR database using this GI list, yielding 5,099,147 sequences (NR-plant). Then, the full set of the coffee-LRS isoforms was submitted to stand-alone BLASTx against the NR database below 1e-10. 2) Sequences without hits from step 1 were submitted further to NCBI non-redundant nucleotide sequences (NT, May 5th, 2017) BLASTn at 1e-10. 3) Sequences without a hit from step 2 were processed further with BLASTn (1e-20) to *C. canephora* coding sequences (CDS) with UTR and *C. arabica* EST database [20, 21]. 4) The output of BLASTx was filtered with query coverage (qcovs), cumulative identity (ID) and sequence length into three categories, high, medium and low quality annotation. Query coverage indicates the input coffee-LRS isoforms covered by the matched sequences. Cumulative identity represents the identity length to the aligned length (AL). ID can be expressed as the ratio of the sum of identity length to the sum of the aligned length of all the Hsps (High-scoring Segment Pairs) of a subject. The four databases above, NR plant, NT, *C. canephora* CDS with UTR and *C. arabica* EST database, are named as FOUR databases in this manuscript. Finally, all the BLASTx and BLASTn results were processed by function annotation with BLAST2GO.

The Blast2GO Pro 4.0 (North America, US: USA2 Version: b2g_Sep 16) pipeline was based on default settings [22]. InterProScan (IPS) was used to search sequence protein domains from EBI databases to improve annotation (North America, US: USA2, Version: b2g_Sep 16). In the follow-up phase, Blast2GO Mapping, Annotation and Annex functions were applied to retrieve GO (gene ontology) terms, select reliable annotations and increase the number of annotated isoforms respectively. The GO-slim tool was used against the plant

database to provide plant generic GOs. Finally, GO enzyme mapping and KEGG (Kyoto encyclopaedia of genes and genomes) pathway maps were loaded.

Case studies with the caffeine and sucrose genes

Two case studies were performed with genes encoding caffeine and sucrose biosynthesis pathway (caffeine and sucrose genes) to investigate specifically the quality, advantage and additional potential of the coffee-LRS isoforms. Reported coffee caffeine and sucrose candidate genes were downloaded from the European Nucleotide Archive (EMBL-EBI) (Table 3 and Table 4).

For potential caffeine candidate genes, coffee-LRS isoforms were processed with BLASTn (1e-20) against the reported caffeine genes. Sequences with hits to the reported caffeine genes were submitted to BLASTx (1e-20) with the NR database to confirm whether they were caffeine genes (higher bit score). Confirmed transcripts (potential caffeine isoforms) and sucrose isoforms annotated by Blast2GO (potential sucrose transcripts) were further evaluated with Geneious 10.0.4 by aligning back to the reported candidate genes in allele level [24]. Motif analysis was conducted with default parameters except for "ten motifs" selected with MEME 4.11.2 [25]. UTRscan was used for UTR functional motifs annotation [26].

Comparison to other available coffee databases

To compare with available coffee sequences, the full coffee-LRS isoforms were processed with BLASTn (1e-20) to *C.canephora* CDS with UTR and *C. arabica EST database*, respectively and the other way around [20, 21]. The *C. eugenioides* transcriptome (young leaves and mature fruits) from Illumina was also used in the comparison [23].

Novel genes

Coffee-LRS isoforms without hits to the FOUR databases were submitted to the Rfam database by Blast2GO Pro package for non-coding RNA analysis [27]. Sequences without hits to Rfam were probably novel genes in coffee.

Analysis of long sequences

In order to explore the advantage of using the LRS PacBio platform to obtain long sequences, the BLASTx and BLAST2GO functional annotation result for the coffee-LRS isoforms longer than 10kb were extracted from the total dataset.

Analyses

Overview of full-Length RNA molecules from long-read sequencing

A total of 2,618,905 raw reads were generated from LRS platform, which yielded 443,877 reads of insert. After 8,842 short sequences (less than 300 bp) were removed, 233,464 full-length (FL) and 201,571 non-full-length (NFL) reads were generated. The individual isoforms were sequenced in average five times. In total, 95,995 coffee-LRS isoforms were recovered after sequences representing chloroplast, mitochondrial and ribosomal transcripts were removed (see Table 1). The length of the sequences in this dataset ranged from 301 bp to 23,335 bp, with an average length of 3,236 bp. The GC content was 41.4% and the N50 was 4,865 bp.

The BLASTx output (against NR plant) was divided into three groups, high, medium and low quality based on Qcov, ID and sequence length (see Data description and Table 2). There were 34,719 (high), 13,655 (medium) and 40,314 (low) sequences were grouped into each quality groups, respectively (see supporting information 1 Table S1). Thereafter, 7,280 sequences without hits were processed with BLASTn to NT database and resulting in 1,981 sequences with hits. A total of 5,299 sequences without a hit were further accessed with *C.*

canephora CDS and UTR and C.arabica contigs. Finally, there were 1,217 sequences with no hits to any of the above databases (FOUR databases).

Functional Annotation

Functional annotation of the coffee-LRS isoforms was investigated using different databases. The data in Table 2 shows that 88,715 sequences (92.42%) had hits to NR plant proteins. A total of 70,774 sequences (73.73%) matched to IPS protein domains with 33,605 IPS GOs (35.01%). A number of 78,571 sequences (81.85%) had identified GOs. After the GOs were merged, GOs of 58,050 sequences (60.47%) matched with GO-slim (plant).

Of all the hits to the NR plant proteins from BLASTx, the coffee-LRS isoforms (maximum 50 hits to each sequences) had the highest number of hits to the *Nicotiana tabacum* (tobacco, 174,6308 hits), followed by *C. canephora* (142,656 hits), *Vitis Vinifera* (grape, 134,025 hits) and *Theobroma cacao* (cacao, 132,336 hits) proteins (see supporting information 1 Figure S1). Most hits found in tobacco were probably because the tobacco database is more extensive and well annotated than those of other related species, like *C. canephora*. For top-hit species, there is no doubt the majority of the sequences has top-hit with the progenitor, *C.canephora* (73,587 sequences), followed by *Sesamum idicum* (1,321 sequences), *Nicotiana tabacum* (767 sequences), etc. (see supporting information Figure S2). The NR-plant database consists of few proteins sequences from Coffea arabica as reflected by just 485 protein sequence hits and is ranked seventh in the top-species hit list. This indicates the limit information on *Coffea arabica*. Of the 33,512 sequences (34.91%) with IPS GOs, cytochrome P450 (IPR001128, 353 matches) had the most sequence matches among the IPS families (see supporting information 1 Fig. S3).

Biological process (BP, 56,230 sequences) was more abundant than cellular component (CC, 44,528 sequences) and molecular function (MF, 45,604 sequences) (see supporting

information 1 Fig. S4). Within these functional groups, the highest number of sequences were annotated with the biosynthetic process (11,627 sequences, 20.68%), membrane component (21,175 sequences, 47.55%) and transferase activity (11,921 sequences, 26.14%). A total of 156 pathways with 921 enzymes were annotated by KEGG, associated with 11.97% of the whole dataset (11,489 sequences). Among these, starch and sucrose metabolism ranked as the fifth most abundant pathways, with 36 encoding enzymes and 766 isoforms annotated (see supporting information 1 Fig. S5). The average number of coffee LRS isoforms encoding the 921 enzymes was 18 while the highest number was found in phosphatase (EC: 3.6.1.15, 2,969 sequences), encoding the purine metabolism and thiamine metabolism pathway. In comparison, only 802 sequences were associated with 142 pathways and 374 enzymes in *C. eugenioides* transcriptome and starch and sucrose pathway relating to 450 contigs was the most encoded pathway [23].

The candidate genes for the major caffeine candidate genes were not identified by KEGG pathway. To evaluate the annotated isoforms and their diversity, further analysis was performed with caffeine pathway. The sucrose pathway was also analysed as a case study as sucrose candidate genes were relatively long and highly diverse. Both of these pathways are important for the understanding of coffee quality [28].

Case study I: Isoform diversity in the caffeine biosynthesis pathway

The caffeine pathway has been widely studied previously (Fig. 2a). Candidate genes and complete coding sequences of both transcripts and genomic DNA are available in public databases and can be used as well-established references for caffeine candidate gene analysis (Table 3). From the BLASTn output, 25 long-read transcripts were annotated and related to candidate caffeine genes. Further alignment suggests ten high quality isoforms were likely to be putative caffeine genes, including three transcript variants of *XMT1*, one of *MXMT1*, one of *MXMT2* together with two of *DXMT1* and three of *DXMT2*. All genes encoding caffeine

the primary pathway except the *XMT2* gene were present in this bean transcriptome (Fig. 2 and Table 3). The length distribution of these isoforms ranged between 977 and 1,517bp.

Importantly, all ten isoforms were extended at the 5' UTR region compared to the corresponding sequences reported in Arabica and Robusta coffee (see Table 3), while eight isoforms were longer at the 3' end (Fig. 2b, 2c, 2e and supporting information 2 Fig. S6). The most extended isoform (c695597/f1p2/1421) was 136 bp longer than the previously reported candidate genes (*CaXMT1*, Fig. 2b). Nine isoforms were found to be longer than the reported genomic DNA sequences. The other isoform was likely to have resulted from an alternative polyadenylation event (c25904/f2p0/977, Fig. 2c) as two potential polyadenylation signals (AAUAAA) were identified in the 3' UTR (Fig 2d). Alternative splicing was also presented in caffeine isoforms, for example, intron retention was detected in one of the putative *DXMT2* isoforms (Fig. 2e).

Coffee LRS isoforms encoding XMT1 (Fig 2b), MXMT1 (supporting information Fig. S6) and DXMT2 (Fig 2c, 2e) were better aligned to the corresponding *C.canephora* isoforms, individually (higher identity, see Fig. 2 and supporting information Fig. S6). This indicates these transcript variants were potentially *C.canephora* sub-genome copies. In contrast, isoforms encoding XMT2 (Fig 2c), MXMT2 (supporting information Fig. S6) and DXMT1 (Fig 2c, 2e) were poorly aligned with *C. canephora* isoforms (more variants) and were probably *C.eugenioides* sub-genome copies.

Case study II: Long sucrose isoforms provide insight into the complexity of the polyploid system

Sucrose genes were used to investigate the transcriptome sequence diversity of the polyploidy system. For the sucrose synthase 1 gene (*SS1)*, one of the important genes in the sucrose metabolism, nine transcript variants were identified (Fig. 3, Table 4 and Fig. 4a). Compared to c86432/f7p9/4842, the other eight transcript variants varied in motif replacement (motif 7

replaced motif 9 in c106591/f2p0/4381), deletion (for example c92344/f1p26/4662) and relocation (intron retention, c92296/f1p5/4676 and c91298/f1p1/3137), etc. (Fig. 4b). The length of these nine putative *SS1* transcript variants ranged from 2,961 to 4,842 bp.

Importantly, all the sucrose transcript variants studied in this research were extended in the 5'UTR region relative to previous reports, except for *SPS1*, c51110/f2p0/3136 (Table 4). Some transcript variants, such as the longest putative *SS1* sequence identified, c86432/f2p7/4842 (4,842 bp), extended 2,131 bp upstream of the *C.canephora* coding sequence (*G-CcSS1*) and 1,994 bp upstream of the Arabica sucrose synthase 1 mRNA coding sequence (*CaSS1*). The length of the 5'leading region of the *SS1* transcript variants ranged between 218 and 2,131 bp (Table 5). To understand the diversity in this region, the 5' leading sequences of the nine putative *SS1* transcript variants were scanned using the UTRdb online server. A maximum of 12 upstream open reading frames (uORFs) were identified and the number was positively correlated with the length of the sequences. No uORFs were identified in the two transcript variants with short 5'UTR, c62911/f29p21/2965 (218 bp leader sequence) and c72639/f25p28/2961 (232 bp leader sequence).

The nine *SS1* transcript variants revealed transcript diversity that resulted largely from different copies from the progenitors. When aligned to *G-CcSS1* (*C. canephora SS1* genomic sequence), the top four putative *SS1* transcript variants showed high identity and consistent nucleotide variants (like the guanine highlighted at 3,726 bp in the consensus sequence, Fig. 4c), suggesting that these were copies from the *C. canephora* sub-genome. For example, compared to the consensus sequence, the same indels were present in 3,707bp and 3,733bp, a cytosine at 3,713bp and guanine at 3,715bp, etc. Consistently, the sequence of intron retention in one of the top four sequences, c91298/f1/p1/3137 (Fig. 4d) shows high homology to the intron sequence of *C. canephora*. However, the bottom five transcript variants had a higher number of variations compared to *G-CcSS1* that are likely to be *C. eugenioides* sub-

genome derived copies. The lower five transcripts had lots of variations compared to *C.canephora* intron 10, further indicating this group was from a different copy, probably *C. eugenioides* (Fig. 4e). Additionally, some alleles of *G-CcSS1* were common in nine putative Arabica *SS1* transcript variants and Arabica sucrose synthase 1(*CaSS1*), such as the variant at 3,666 bp (Fig. 4e). This type of allele probably results from different genotypes. Polyploid expression patterns were also observed in *SP1* transcript variants, the top two alignments were similar to *C.canephora* and the other two were slightly different but related. All of the four transcript variants were longer in the upstream sequences while three extended further downstream than had previously been reported.

Another essential potential of LRS is to explore sequences not yet complete or published. For instance, four transcript variants were identified from this research while *SPS2* has only been identified in *C. canephora* rather than *C. arabica* (Fig. 4f).

Comparison to other available coffee databases

To understand the advantage and the diversity of this polyploid coffee transcriptome, a comparison was made with the available coffee database. More than twice the number of isoforms were identified in the tetraploid Arabica LRS transcriptome (immature, intermediate and mature fruits) compared with the *C. eugenioides* contigs (36,935 de novo assembled contigs, average length: 701 bp, from immature leaves and mature fruits), *C.canephora* CDS with UTR (25,570 sequences, from a variety of tissues, including fruits) and *C. arabica* EST database (35,153 contigs, including fruits) (see Table 2) [14, 20, 23]. The coffee-LRS isoforms show greater transcript length, diversity and a lower GC content. The N50 of the Pacbio dataset (4,865 bp) was more than three times longer and the average length was more than twice that of the other databases. The sequence distribution of *C.arabica* contigs peaks at 655 bp while *C.canephora* CDS with UTR reaches the largest number of sequences at

1,490 bp (Fig 5). Most of the sequences from the *C. canephora* CDS with UTR and the *C. arabica* EST database were less than 3,770 bp. By comparison, 39,917 coffee LRS isoforms (41.6%) were longer than 3,770 bp

Results of the BLASTn analysis indicated that of the 95,995 coffee-LRS isoforms, 9,308 (9.7%) had no matches to the *C.canephora* CDS with UTR while 3,682 (3.8%) isoforms had no hits to the *C.arabica* contigs. This indicates coffee-LRS isoforms are very diverse compared to these two databases. Conversely, 9,167 (26.1%) of *C.canephora* CDS with UTR and 4,830 (18.9%) of *C.arabica* contigs had no hits to the coffee-LRS isoforms. These two sets of sequences without hits are probably sequences from leaf or other tissues not expressed in the tissues investigated in this study.

Novel genes

The 1,217 sequences without hits to the FOUR databases (NR plant proteins, NT database, *C. canephora* CDS with UTR and *C. arabica* EST database) were submitted to the Rfam server to predict non-coding RNAs (ncRNA). The four isoforms that matched were in three biotypes, two transcripts were identified as CD-box snoRNA, one as HACA-box snoRNA and the other one as a miRNA (see supporting information Table S2). Other than these, the other 1,213 sequences had no hit to the FOUR databases and Rfam are likely to be novel genes that have not been discovered in coffee or contaminants from other organisms with no sequence information to date. Length distribution of this new dataset ranged from 325 to 19,189 bp.

Long transcripts

In order to assess the value of LRS in discovering long sequences, 577 transcripts longer than 10 kb were further analysed. Functional annotation of this extremely long dataset shows the majority of the sequences (564 sequences, 97.8%) matched to the FOUR databases. The

HSP/Hit coverage distribution was relatively evenly distributed from 0 to 100% compared to the HSP/Seq coverage. In parallel, the majority of sequences distributed less than 50% HSP/Seq coverage and peaked at 6%, representing limited information of long sequences in the NR database. IPS matches were found for 352 sequences (61.0%) while 61 of them had IPS GOs. A total of 446 sequences (77.3%) were retrieved with GO terms, while 201 isoforms (34.8%) from these were also annotated with GO-Slim.

In total, 144 sequences were classified into the biological process, with 92 sequences into cellular component and 79 into molecular function (see supporting information Table Fig. S7). Among them, biosynthetic process (31 sequences), member (43 sequences) and hydrolase activity (25 sequences) were the top groups, separately, from the three functional process. Among the annotated isoforms, 18 sequences encoding 12 enzymes from 13 pathways were annotated with a KEGG pathway. The starch and sucrose metabolism ranking the third most encoded pathway with two isoforms encoding two enzymes.

Discussion

Full-length transcripts generated by LRS in this study provided an isoform level polyploid coffee bean reference transcriptome. Compared to its sub-genome progenitors, the Arabica coffee bean transcriptome was more diverse and complicated with more isoforms, enzymes and pathways. Case studies in caffeine and sucrose identified that this diversity and complexity were a result of alternative splicing, polyadenylation, 5’UTR extension and sub-genome copies. Discovery of novel genes and long transcripts was also an advantage of using the LRS technology.

Polyploid expression

Different transcript variants may vary in function within the cell and be differentially expressed in tissues or environmental conditions. The abundance of variants in the Arabica

transcriptome and case studies of caffeine and sucrose genes compared to the sub-genome progenitors clearly shows the complexity of the polyploid expression.

Generally, polyploidy results in three main expression patterns of non-additive expression, dominant expression in which total gene expression in the hybrid is similar to one of the parents, transgressive expression compared to the progenitors or unequal homeolog expression [1]. Previously, it was proposed in coffee that the lower caffeine in Arabica coffee was due to the *C. eugenioides* sub-genome attributes. Based on phylogenetic analysis, CaXMT1, CaMXMT1 and CaDXMT2 were believed to be from the *C. canephora* sub-genome while CaXMT2, CaMXMT2 and CaDXMT1 were from the *C.eugenioides* sub-genome[29]. *C. eugenioides* has a very low caffeine biosynthesis together with a rapid catabolism [30]. The expression of sub-genome copies from *C.eugenioides* suggested lower caffeine in Arabica coffee compared to Robusta coffee. This study supports this hypothesis of transcript variants from sub-genome copies controlling the trait.

Using the LRS isoforms, further studies are now possible at the isoform level (this study was at the transcript variant level) to understand sub-genome gene expression in the polyploid *C arabica*. First, it will be possible to determine directly whether the expression of Arabica caffeine genes follows a non-additive expression pattern. Secondly, it would be interesting to determine the reason for more transcript variants identified is similar to those from the *C. canephora* sub-genome copies of the XMT and DXMT genes and their differential expression in tissues and at development stages. Thirdly, it will be possible to determine whether this expression pattern is influenced by environment, influencing coffee quality. Fourth, whether these different gene expression patterns result in different phenotypes. Similar analysis could be applied to many other genes or pathways of interest. Isoforms and transcript variants found in LRS tetraploid Arabica coffee bean transcriptome in this study were assigned to a number of functional groups, pathways and to specific enzyme functions.

Arabica is believed to be more adaptive to temperature change than its diploid parents [31]. This study may also help elucidate the genetic basis of the higher sucrose in Arabica coffee. More generally, the complete polyploid transcriptome from this study will improve our understanding of the evolutionary adaptation and plasticity of polyploid species. However, further improvement is still needed in LRS technologies to improve the sequencing depth. Candidate genes in the caffeine pathway are reported to be expressed at low levels in fruits compared to leaves, especially XMT2 (detected by quantitive RT-PCR) [29]. Transcripts were not detected in this study probably due to low expression of XMT2 and the PacBio iso-seq technology not being sensitive enough to capture these transcripts. This is likely to happen in the case of other isoforms expressed at low levels that may not be captured by the Iso-Seq technology even after application of the cDNA library normalisation step, as was applied in this study.

5'UTR extension

Full-length transcripts captured in this study show the advantage of LRS. All the caffeine and sucrose isoforms annotated in this study, except for *SPS1*, were extended in the 5'UTR compared to those available from public databases. Previously, it was difficult to sequence the 5' end as cDNA library preparation starts from the 3' end and normally fails to reach the 5'end. Further, it was not easy to assemble the non-coding parts of transcripts as limited cDNA sequence was available to guide the assembly and confirm the contigs obtained. Therefore, less information is available on the 5'UTRs, especially for plants. Generally, the length of the 5'UTR ranges from 100 up to a few thousand bp [32]. This length difference is proposed because of the complex gene regulation maintained in eukaryotes [33]. Few post-transcriptional mechanisms have been studied in 5'UTRs, including the regulation by the pre-initiation complex and uORF re-initiation.

uORFs are common in 5'UTRs that have critical regulation. They contain their own set of start and stop codons that can be scanned by ribosomes and translated. This regulation can inhibit translation of the main ORF transcript and reduce the amount of protein translated. Regulation of re-initiation of uORF translation was found to be associated with the length of sequence between the uORF and the main ORF, suggesting interactions with translation factors are required before initiation of translation [34]. This was also shown to be influenced by stress conditions [34]. However, not all uORF may have a role in translation control. In the leucine zipper transcription factor (*bZIP*) 11 gene, for example, harbouring four uORFs, only uORF2 was required for this regulation and this uORF is relatively conserved [35]. Other types of 5'UTR regulation may also be found such as that due to introns in the 5'UTRs. This happens to approximately 35% of human genes [6].

Understanding the mechanism of 5'UTR regulation will be greatly facilitated by the use of the full-length transcripts. In this study, multiple uORFs were characterised in the *SS1* 5' UTR and these may contribute to diverse functions and regulation that may be influenced by stress conditions. Climate change is a threat to Arabica coffee, which grows at high altitude. It may be possible to influence 5'UTR regulation in Arabica coffee and have the potential to influence coffee quality. To confirm this, further phenotype, proteome and metabolome studies are required.

Long transcripts

LRS also has potential in discovering long transcripts, such as the sucrose synthase genes annotated here. Even though numerous studies have defined the sucrose pathways, not all the candidate genes have been identified. Many sucrose metabolism genes are too long to be captured by short read sequencing without significant *de novo* assembly. For example, the *C. arabica SS2* coding sequence is 2,889 bp and the genomic DNA sequence (exon 1 to 15) is 5,672 bp (Table 4). Sucrose synthase genes (6-7 different isoforms) were previously

identified in cotton, rice, and Arabidopsis, However, in coffee, only two had been reported [36-38]. For genes that were only previously available for *C. canephora*, (e.g. *SP1*), this study also identified isoforms in Arabica. For genes that previously only had partial sequences available, (e.g. *SPS2*), the transcripts identified in this study will guide further studies and improve current databases. Furthermore, the low coverage annotation of long sequences (>10kb) by BLASTx and BLASTn against the FOUR databases indicated the limited information on long sequences requiring further study.

Transcriptome analysis of polyploids using long-read sequencing

LRS technologies show advantages in understanding complex transcriptomes, especially from polyploid species [4, 39, 40]. First, this eliminates transcriptome reconstruction and that reduces the computation time. This is an essential goal for bioinformatics data analysis and software development [41]. To avoid obsolescence, transcriptome analysis calls for rapid genomics and bioinformatics to reduce the time from experiment to publication. Secondly, as there is no assembly of reads with LRS, there are no erroneous results due to misassemblies caused by complex polyploid transcriptomes with a large number of repeats or homeolog genes. For example, almost 80% of the wheat genome is repetitive [40]. Last but not least, it shows the potential to capture rare or long sequences to provide an overview of the transcriptome and fully characterise RNA diversity, like 5'UTR extension in this study, alternative splicing, polyadenylation, etc. [4, 42].

However, LRS technologies have been normally biased with high error rates, for example, previously released PacBio single molecule real-time sequencing (SMRT) reads had a very high error rate, 11-14%, therefore, numerous methods have been proposed to correct the sequences [43]. One common approach was to map back to a reference genome and (or) use hybrid sequencing, for example, using short reads with high throughput to correct LRS isoform sequences [5, 44]. However, caution is necessary when using this strategy. The

reference genome is often far from 100% accurate: 1) most draft genomes have numerous fragmented contigs or scaffolds with huge imbedded gaps. Even genomes previously considered well assembled have had many gaps[45]. 2) Problems also exist in poorly assembled gene loci. Few recently released genomes have been re-visited to generate improved assemblies [13]. 3) LRS isoform sequences normally come from different sources (e.g. genotype) to the reference genomes that they can be compared with. Hybrid sequencing correction may have system bias and result in loss of isoforms/transcript variants or generate a "compromised" consensus. Previously, it has been estimated that there was no approach that has achieved more than 60 % accuracy for transcript reconstruction, even for the most studied human genome [46]. For instance, short read platforms deliver data that is less representative of rare or long isoforms and there is a high chance of losing these reads from the long-read dataset when correcting.

Improved accuracy may be generated from the platform itself, for example, Pacbio Iso-seq generates improved accuracy from CCS reads. This allows multiple passes of each transcript. Each pass can be used to correct the others with their random errors (mainly indels). The isoform clustering and polishing in this protocol is expected to deliver 99% accuracy. Prior to size selection, normalisation was further applied in parallel to the dataset in this study to decrease the frequency of abundant reads and produce a more even representation of the transcriptome and to capture rare sequences. A highly diverse transcriptome has resulted. The abundance of genes that had not been previously sequenced (1,213), transcript variants and longer isoforms indicate the limits of previous studies and potential of LRS technologies. However, the limitation shows in detecting short sequences less than 300bp (raw data cut-off). The chances of large errors due to indels from Pacbio sequencing may produce reads shorter than the actual reads. Additionally, the Blue pippin size selection system starts from

500bp in the cDNA library preparation, with few sequences from the boundary (400-500bp). Therefore, improvement is needed to capture a broader transcriptome.

In conclusion, this study will improve the understanding of the biology and genetic improvement of polyploid species such as coffee. It provides a useful technique to generate a full-length reference transcriptome and improve understanding of UTR regions.

Additional information

New sequence data used in this manuscript has been submitted to European Nucleotide Archive at EMBL database with accession number: PRJEB19262. Additionally information on specific selected sequence IDs, such as high quality annotated sequences, novel genes in coffee, etc, are shown in supporting information 2.

Completing interests

All authors have no conflicts of interest to this manuscript.

Funding

This study was funded by Australian Research Council (PROJECT ID: LP130100376) and Chinese Scholarship Council (2014-2018).

Author’s contributions

B.C., A.F. and R.H. designed the research and discussed the results. B.C performed the experiment and analysis. B.C drafted the manuscript, R.H and AG refined it.

Acknowledgements

We thank Green Cauldron Coffee (Australia) for providing coffee materials, Prathima Perumal Thirugnanasambandam for assistance in sucrose synthase analysis, Kevin Smith and Erli Wang for help in the informatics pipeline and the Research Computing Center of the

University of Queensland, Australia for access to high-performance computers. We also appreciated the help from Poss Reading, Marta Brozynska, Adam Healey, Tiparat Tikapunya, Ravi Nirmal, Nam Hoang and Hayba Badro in coffee sampling.

Reference

1. Yoo, M.-J., et al., *Nonadditive gene expression in polyploids.* Annual review of genetics, 2014. **48**: p. 485-517.
2. Adams, K.L., et al., *Genes duplicated by polyploidy show unequal contributions to the transcriptome and organ-specific reciprocal silencing.* Proceedings of the National Academy of sciences, 2003. **100**(8): p. 4649-4654.
3. Levasseur, A. and P. Pontarotti, *The role of duplications in the evolution of genomes highlights the need for evolutionary-based approaches in comparative genomics.* Biology direct, 2011. **6**(1): p. 11.
4. Wang, B., et al., *Unveiling the complexity of the maize transcriptome by single-molecule long-read sequencing.* Nature Communications, 2016. **7**.
5. Abdel-Ghany, S.E., et al., *A survey of the sorghum transcriptome using single-molecule long reads.* Nature Communications, 2016. **7**.
6. Bicknell, A.A., et al., *Introns in UTRs: why we should stop ignoring them.* Bioessays, 2012. **34**(12): p. 1025-1034.
7. Mignone, F., et al., *Untranslated regions of mRNAs.* Genome biology, 2002. **3**(3): p. reviews0004. 1.
8. Van Veen, H., et al., *Transcriptomes of eight Arabidopsis thaliana accessions reveal core conserved, genotype-and organ-specific responses to flooding stress.* Plant physiology, 2016: p. pp. 00472.2016.
9. Garg, R., et al., *Transcriptome analyses reveal genotype-and developmental stage-specific molecular responses to drought and salinity stresses in chickpea.* Scientific reports, 2016. **6**.
10. Grabherr, M.G., et al., *Full-length transcriptome assembly from RNA-Seq data without a reference genome.* Nature biotechnology, 2011. **29**(7): p. 644-652.
11. Wang, X.-W., et al., *De novo characterization of a whitefly transcriptome and analysis of its gene expression during development.* BMC genomics, 2010. **11**(1): p. 400.
12. Li, P., et al., *The developmental dynamics of the maize leaf transcriptome.* Nature genetics, 2010. **42**(12): p. 1060-1067.
13. Michael, T.P. and R. VanBuren, *Progress, challenges and the future of crop genomes.* Current opinion in plant biology, 2015. **24**: p. 71-81.
14. Denoeud, F., et al., *The coffee genome provides insight into the convergent evolution of caffeine biosynthesis.* science, 2014. **345**(6201): p. 1181-1184.
15. Fridell, G., *Coffee*. 2014: John Wiley & Sons.
16. Furtado, A., *RNA Extraction from Developing or Mature Wheat Seeds.* Cereal Genomics: Methods and Protocols, 2014: p. 23-28.
17. PacificBiosciences. *RS_IsoSeq (v2.3) Tutorial 2*. 2. Isoform level clustering (ICE and Quiver) 2015; Available from: https://github.com/PacificBiosciences/cDNA_primer/wiki/RS_IsoSeq-%28v2.3%29-Tutorial-%232.-Isoform-level-clustering-%28ICE-and-Quiver%29.
18. Fu, L., et al., *CD-HIT: accelerated for clustering the next-generation sequencing data.* Bioinformatics, 2012. **28**(23): p. 3150-3152.
19. Afgan, E., et al., *Genomics Virtual Laboratory: a practical bioinformatics workbench for the cloud.* PloS one, 2015. **10**(10): p. e0140829.

20. Mondego, J.M., et al., *An EST-based analysis identifies new genes and reveals distinctive gene expression features of Coffea arabica and Coffea canephora.* BMC plant biology, 2011. **11**(1): p. 1.
21. Dereeper, A., et al., *The coffee genome hub: a resource for coffee genomes.* Nucleic acids research, 2015. **43**(D1): p. D1028-D1035.
22. Götz, S., et al., *High-throughput functional annotation and data mining with the Blast2GO suite.* Nucleic acids research, 2008. **36**(10): p. 3420-3435.
23. Yuyama, P.M., et al., *Transcriptome analysis in Coffea eugenioides, an Arabica coffee ancestor, reveals differentially expressed genes in leaves and fruits.* Molecular Genetics and Genomics, 2016. **291**(1): p. 323-336.
24. Kearse, M., et al., *Geneious Basic: an integrated and extendable desktop software platform for the organization and analysis of sequence data.* Bioinformatics, 2012. **28**(12): p. 1647-1649.
25. Bailey, T.L., et al., *MEME SUITE: tools for motif discovery and searching.* Nucleic acids research, 2009: p. gkp335.
26. Grillo, G., et al., *UTRdb and UTRsite (RELEASE 2010): a collection of sequences and regulatory motifs of the untranslated regions of eukaryotic mRNAs.* Nucleic acids research, 2010. **38**(suppl 1): p. D75-D80.
27. Nawrocki, E.P., et al., *Rfam 12.0: updates to the RNA families database.* Nucleic acids research, 2014: p. gku1063.
28. Cheng, B., et al., *Influence of genotype and environment on coffee quality.* Trends in Food Science & Technology, 2016.
29. Perrois, C., et al., *Differential regulation of caffeine metabolism in Coffea arabica (Arabica) and Coffea canephora (Robusta).* Planta, 2015. **241**(1): p. 179-191.
30. Ashihara, H. and A. Crozier, *Biosynthesis and catabolism of caffeine in low-caffeine-containing species of Coffea.* Journal of agricultural and food chemistry, 1999. **47**(8): p. 3425-3431.
31. Combes, M.C., et al., *Contribution of subgenomes to the transcriptome and their intertwined regulation in the allopolyploid Coffea arabica grown at contrasted temperatures.* New phytologist, 2013. **200**(1): p. 251-260.
32. Lodish, H., *Molecular cell biology*. 2008: Macmillan.
33. Rhind, N., et al., *Comparative functional genomics of the fission yeasts.* Science, 2011. **332**(6032): p. 930-936.
34. Somers, J., T. Pöyry, and A.E. Willis, *A perspective on mammalian upstream open reading frame function.* The international journal of biochemistry & cell biology, 2013. **45**(8): p. 1690-1700.
35. Hummel, M., et al., *Sucrose-mediated translational control.* Annals of botany, 2009: p. mcp086.
36. Chen, A., et al., *Analyses of the sucrose synthase gene family in cotton: structure, phylogeny and expression patterns.* BMC plant biology, 2012. **12**(1): p. 85.
37. Hirose, T., G.N. Scofield, and T. Terao, *An expression analysis profile for the entire sucrose synthase gene family in rice.* Plant Science, 2008. **174**(5): p. 534-543.
38. Bieniawska, Z., et al., *Analysis of the sucrose synthase gene family in Arabidopsis.* The Plant Journal, 2007. **49**(5): p. 810-828.
39. Minoche, A.E., et al., *Exploiting single-molecule transcript sequencing for eukaryotic gene prediction.* Genome biology, 2015. **16**(1): p. 1.
40. Dong, L., et al., *Single-molecule real-time transcript sequencing facilitates common wheat genome annotation and grain transcriptome research.* BMC genomics, 2015. **16**(1): p. 1039.
41. Haas, B.J., et al., *De novo transcript sequence reconstruction from RNA-seq using the Trinity platform for reference generation and analysis.* Nature protocols, 2013. **8**(8): p. 1494-1512.

42. Gonzalez-Garay, M.L., *Introduction to isoform sequencing using pacific biosciences technology (Iso-Seq)*, in *Transcriptomics and Gene Regulation*. 2016, Springer. p. 141-160.
43. Roberts, R.J., M.O. Carneiro, and M.C. Schatz, *The advantages of SMRT sequencing.* Genome biology, 2013. **14**(7): p. 1.
44. Xu, Z., et al., *Full‐length transcriptome sequences and splice variants obtained by a combination of sequencing platforms applied to different root tissues of Salvia miltiorrhiza and tanshinone biosynthesis.* The Plant Journal, 2015. **82**(6): p. 951-961.
45. Lamesch, P., et al., *The Arabidopsis Information Resource (TAIR): improved gene annotation and new tools.* Nucleic acids research, 2012. **40**(D1): p. D1202-D1210.
46. Korf, I., *Genomics: the state of the art in RNA-seq analysis.* Nature methods, 2013. **10**(12): p. 1165-1166.

Tables and figure legends

Table 1 Arabica long-read sequencing transcriptome annotation with different databases

Databases	Number of sequences annotated	% of sequences annotated
Long-read sequencing transcriptome	95,995	-
BLAST	94,709	98.66
Mapped	78,571	81.85
InterProScan	70,774	73.73
InterProScan GOs	33,605	35.01
GO slim	58,050	60.47
KEGG	11,489	11.97

Table 2 Arabica long-read sequencing isoforms compared to *Coffea canephora* coding sequences and *Coffea arabica* EST sequences

Different datasets	GC content %	N50 (bp)	average length (bp)	min length (bp)	max_length (bp)	Number of sequences
Coffea arabica EST database [1] [20]	44.7	734	662	32	3,584	35,153
Coffea canphora coding sequences with UTR [2]	42.6	2,046	1,616	45	17,206	25,570
***Coffea arabica* long-read sequencing isoforms**	**41.4**	**4,865**	**3,236**	**301**	**23,335**	**95,995**

Note: [1] http://bioinfo03.ibi.unicamp.br/coffea/data/CA.fasta; [2] http://coffee-genome.org/sites/coffee-genome.org/files/download/coffea_cds.fna.gz.

Table 3 Details of caffeine candidate genes, putative transcript variants annotated and 5'UTR extension information

Candidate genes	Accession number	Species	Source	Abbreviation	length (bp)	completeness	Putative transcript variants from LRS isoform sequences	5'UTR extension
xanthosine methyltransferase 1	AB048793	*C. arabica*	mRNA	CaXMT1	1,316	YES	c69597/f1p2/1421<br>c154338/f1p2/1360<br>c71416/f3p3/1376	YES
	JX978514	*C. arabica*	Genomic DNA	G-CaXMT1	1,987	YES		
	DQ422954	*C. canephora*	mRNA	CcXMT1	1,316	YES		
	JX978509	*C. canephora*	Genomic DNA	G-CcXMT1	1,994	YES		
xanthosine methyltransferase2	JX978515	*C. arabica*	Genomic DNA	G-CaXMT2	2,038	YES	Not identified	-
7-methylxanthine N-methyltransferase 1	AB048794	*C. arabica*	mRNA	CaMXMT1	1,298	YES	c20397/f5p1/1361	YES
	JX978511	*C. arabica*	Genomic DNA	G-CaMXMT1	1,838	YES		
	HQ616707	*C. canephora*	mRNA	CcMXMT1	1,222	YES		
	JX978507	*C. canephora*	Genomic DNA	G-CcMXMT1	1,829	YES		
7-methylxanthine N-methyltransferase 2	AB084126	*C. arabica*	mRNA	CaMXMT2	1,155	YES	c10402/f2p3/1277	YES
	JX978512	*C. arabica*	Genomic DNA	G-CaMXMT2	2,010	YES		
3,7-dimethylxanthine N-methyltransferase 1	AB084125	*C. arabica*	mRNA	CaDXMT1	1,155	YES	c25904/f2p0/977<br>c71881/f6p2/1386	YES
	JX978510	*C. arabica*	Genomic DNA	G-CaDXMT1	2,063	YES		
3,7-dimethylxanthine N-methyltransferase 2	KJ577793	*C. arabica*	mRNA	CaDXMT2	1,155	YES	c63815/f1p2/1273<br>c48759/f1p1/1517<br>c26870/f6p5/1402	YES
	KJ577792	*C. arabica*	Genomic DNA	G-CaDXMT2	2,006	YES		
	DQ422955	*C. canephora*	mRNA	CcDXMT1	1,364	YES		

Table 4 Details of sucrose candidate genes, putative transcript variants annotated and 5‘UTR extension information

Candidate genes	Accession number	Species	Source	Abbreviation	length (bp)	completeness	Putative transcript variants from LRS isoform sequences	5'UTR extension
Sucrose synthase 1	AM087674.1	*C. arabica*	mRNA	CaSS1	2,979	YES	c86432/f7p9/4842<br>c91298/f1p1/3137<br>c84406/f3p18/2975<br>c62911/f29p21/2965<br>c92344/f1p26/4662<br>c92296/f1p5/4676<br>c89510/f1p6/4592<br>c106591/f2p0/4381<br>c72639/f25p28/2961	YES
	DQ834312.1	*C. canephora*	mRNA	CcSS2	2,989	YES		
	AJ880768.2	*C. canephora*	Genomic DNA	G-CcSS1	3,957	exon 1-13		
Sucrose synthase 2	AM087675.1	*C. arabica*	mRNA	CaSS2	2,889	YES	c73322/f3p2/3080<br>c75363/f3p2/2906	YES
	AM087676.1	*C. canephora*	Genomic DNA	G-CcSS2	5,672	exon 1-15		
Sucrose phosphate synthase 1	DQ834321.1	*C. canephora*	mRNA	CcSPS1	3,150	YES	c51110/f2p0/3136	YES
	DQ842233.1	*C. canephora*	Genomic DNA	G-CcSPS1	8,215	YES		
Sucrose phosphate synthase 2	DQ842234.1	*C. canephora*	Genomic DNA	G-CcSPS2	1,550	NO	c103631/f1p2/4695<br>c88660/f2p0/4282<br>c106342/f1p4/4274<br>c104672/f1p1/4440<br>(reverse)	YES

Table 5 Results of 5’ UTRs from long-read sequencing scanned with UTRdb. uORF, Upstream Open Reading Frame.

No.	Sequence name	5' UTR length (bp)	uORF
1	c86432/f2p7/4842	2,131	12
2	c91298/f1p1/3137	347	2
3	c84406/f3p18/2975	242	2
4	c62911/f29p21/2965	218	0
5	c92344/f1p26/4662	1,981	10
6	c92296/f1p5/4676	1,884	12
7	c89510/f1p6/4592	1,871	11
8	c106591/f2p0/4381	1,683	11
9	c72639/f25p28/2961	224	0

Figure 1 Coffee fruits of immature, intermediate and mature stages

Figure 2 Sequence distribution, comparison of the number of sequences and their length. Coffee long-read sequencing isoforms, *C.canephora* coding sequences with UTR and *C. arabica EST database* were included.

Figure 3 Putative transcript variants from long-read sequencing aligned to reference caffeine genes. a. Main caffeine biosynthesis pathway in coffee, adaptive from Cheng, Furtado [28]. b. Alignment of three Arabica putative XMT1 variants from long-read sequencing (c69597/f1p2/1412, c154338/f1p2/1360 and c71416/f3p3/1376), *Coffea arabica* and *Coffea canephora* XMT1 (CaXMT1 and CcXMT1) to Arabica XMT1 genomic DNA sequence (G-CaXMT1). c. Possible alternative polyadenylation of putative XMT1 Iso-seq variant (c25904/f2p0977) from long-read sequencing; G-CaDXMT1, Arabica DXMT1 genomic DNA sequence; CaDXMT1, DXMT1 coding sequence; d. Two polyadenylation signals were identified in 3’ends of c25904/f2p0/977; e. Possible alternative splicing (intron retention) in one of the putative DXMT2 variants (c48759/f1p1/1517) from long-read sequencing transcripts; G-CaDXMT2, Arabica DXMT2 genomic DNA sequence; CaDXMT2, Arabica DXMT2 coding sequence. (Note: black colour in the alignment means different nucleotides to reference sequence, Arabica genomic XMT1, while grey colour means the same nucleotides as the reference.).

Figure 4 Motif search results of putative sucrose synthase gene 1 from long read sequencing. a. Ten motifs were annotated in 9 putative sucrose synthase 1 variants from long-read sequencing, analysed by MEME 4.11.2. b. Motif location of 9 putative sucrose synthase 1 variants. Different motifs were highlighted with red arrows and intron retention was shown with dashed boxes.

Figure 5 Putative variants from long-read sequencing aligned to the reference sucrose genes. a. Possible sucrose metabolism in coffee; SS, sucrose synthase; SPS, sucrose phosphate synthase; SP, sucrose phosphatase; INV, invertase; CINV, cell wall invertase (modified from Cheng B. et al. (2016)); b. Alignment of 9 Putative Sucrose synthase variants from long-read sequencing and *C.arabica* sucrose synthase gene 1 (CaSS1) to *Coffea canephora* genomic sucrose synthase 1 (exons 1-13) (G-CcSS1 (1-13)); Green box highlights variants result from different sub-genome copies, while intron retention events were marked with the blue box highlight; c. polyploid expression when zooming green area in 100%; d. possible alternative splicing (intron retention) from a *C.canephora* sub-genome copy when zooming blue box in 100%; e. possible intron retention from a *C.eugenioides* sub-genome copy when zooming blue area in 100%.red line classifies two groups of variants as different sub-genome copies. Different nucleotides compared to the consensus were highlighted in black in the alignment; f. Putative variants from long read sequencing aligned with *C.canephora* genomic sucrose phosphate synthase 2 sequence (G-CcSPS2); FWD, forward sequence; REV, reverse sequence. Different nucleotides compared to the consensus were highlighted in black in the alignment.

Figure 1

Click here to download Figure Fig 1.tif

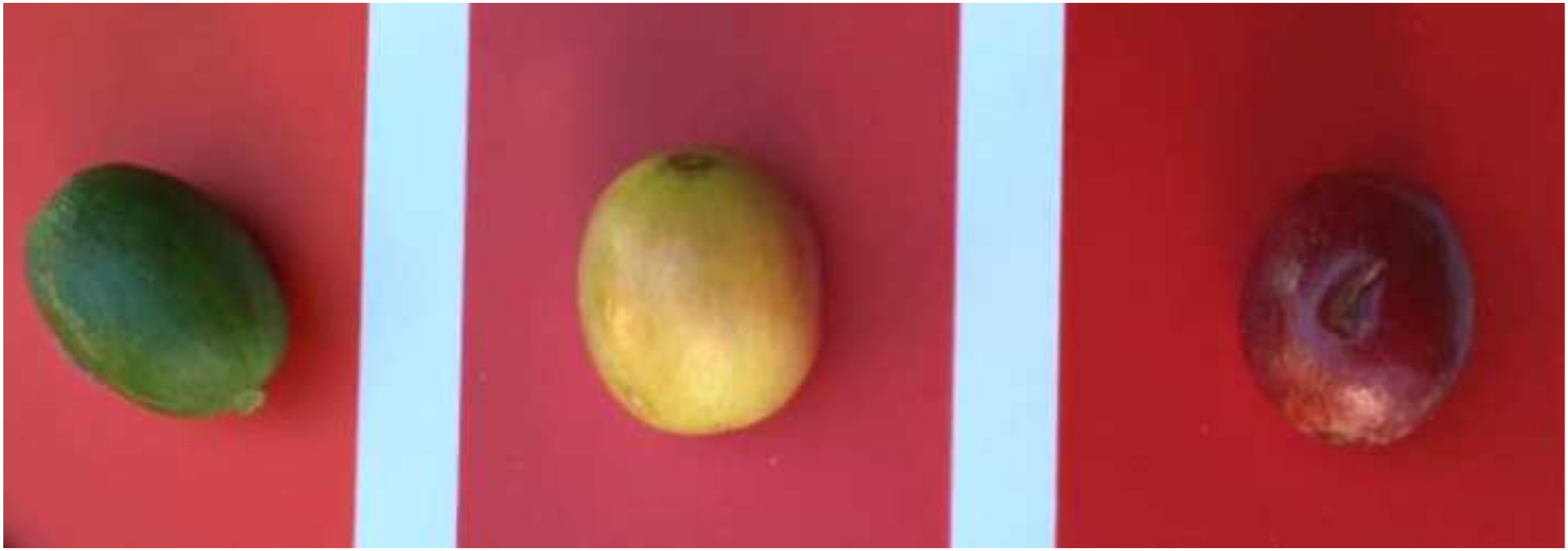

Figure 2a Click here to download Figure Fig 2a.tif

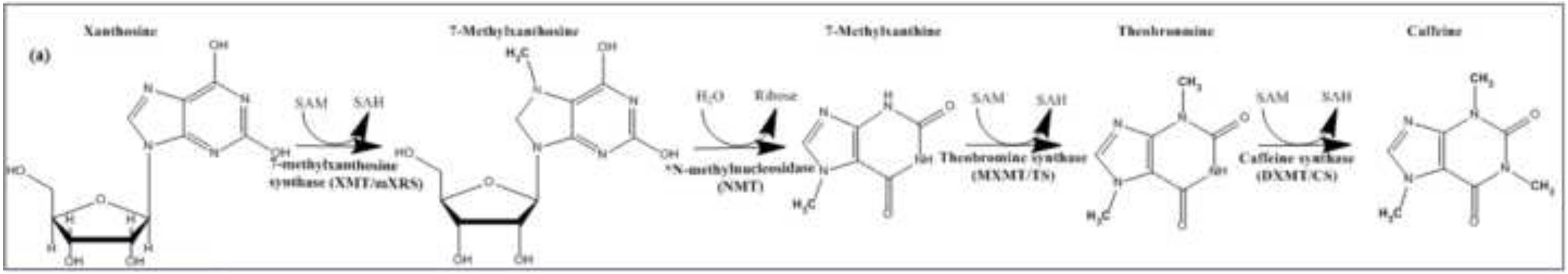

Figure 2b

Click here to download Figure Fig 2b.tif

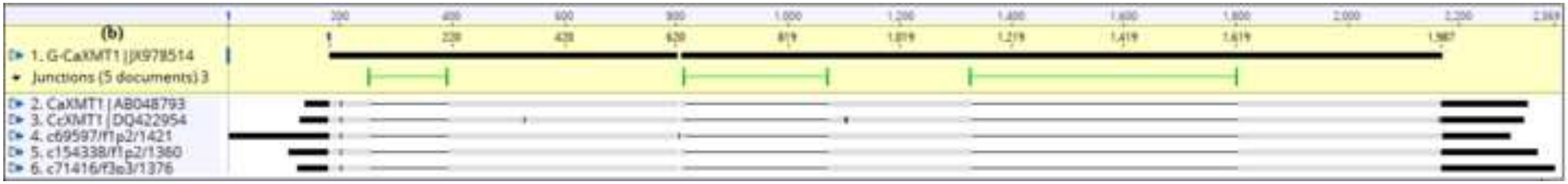

Figure 2c

Click here to download Figure Fig 2c.tif

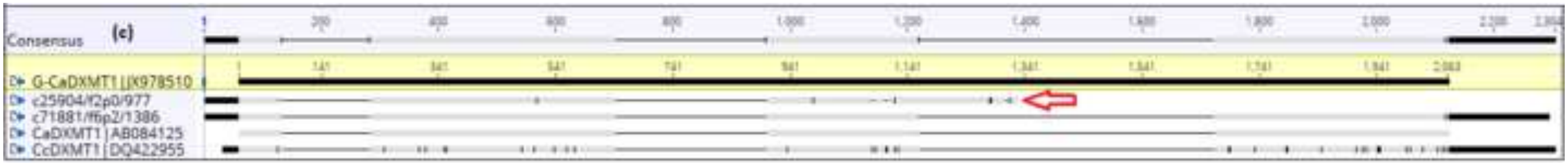

Figure 2d

Click here to download Figure Fig 2d.tif

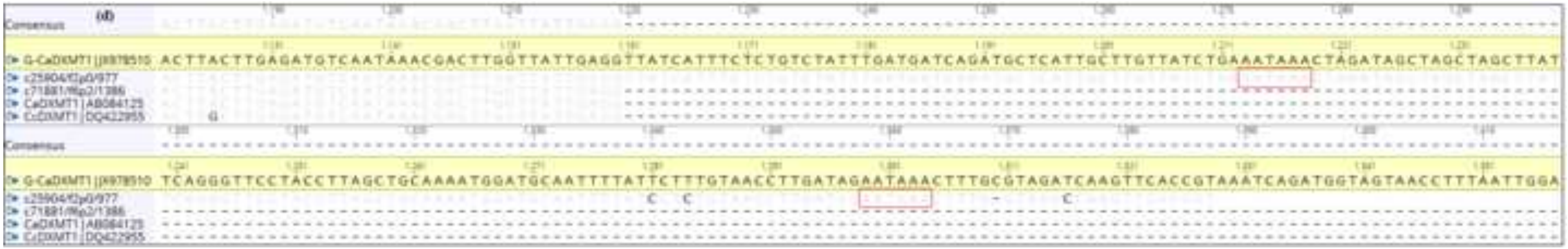

Figure 2e

Click here to download Figure Fig 2e.tif

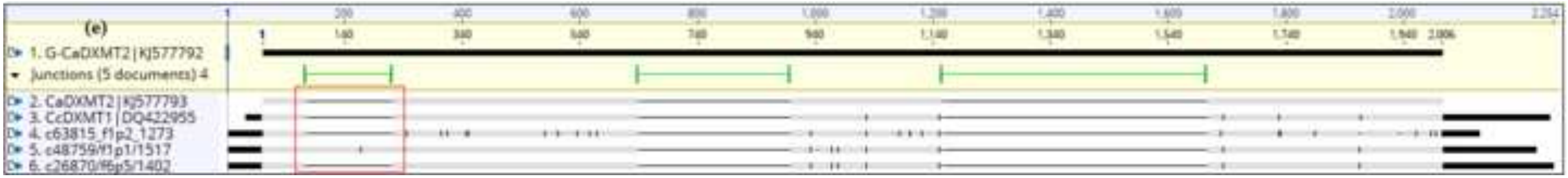

Figure 3a

Click here to download Figure Fig 3a.tif

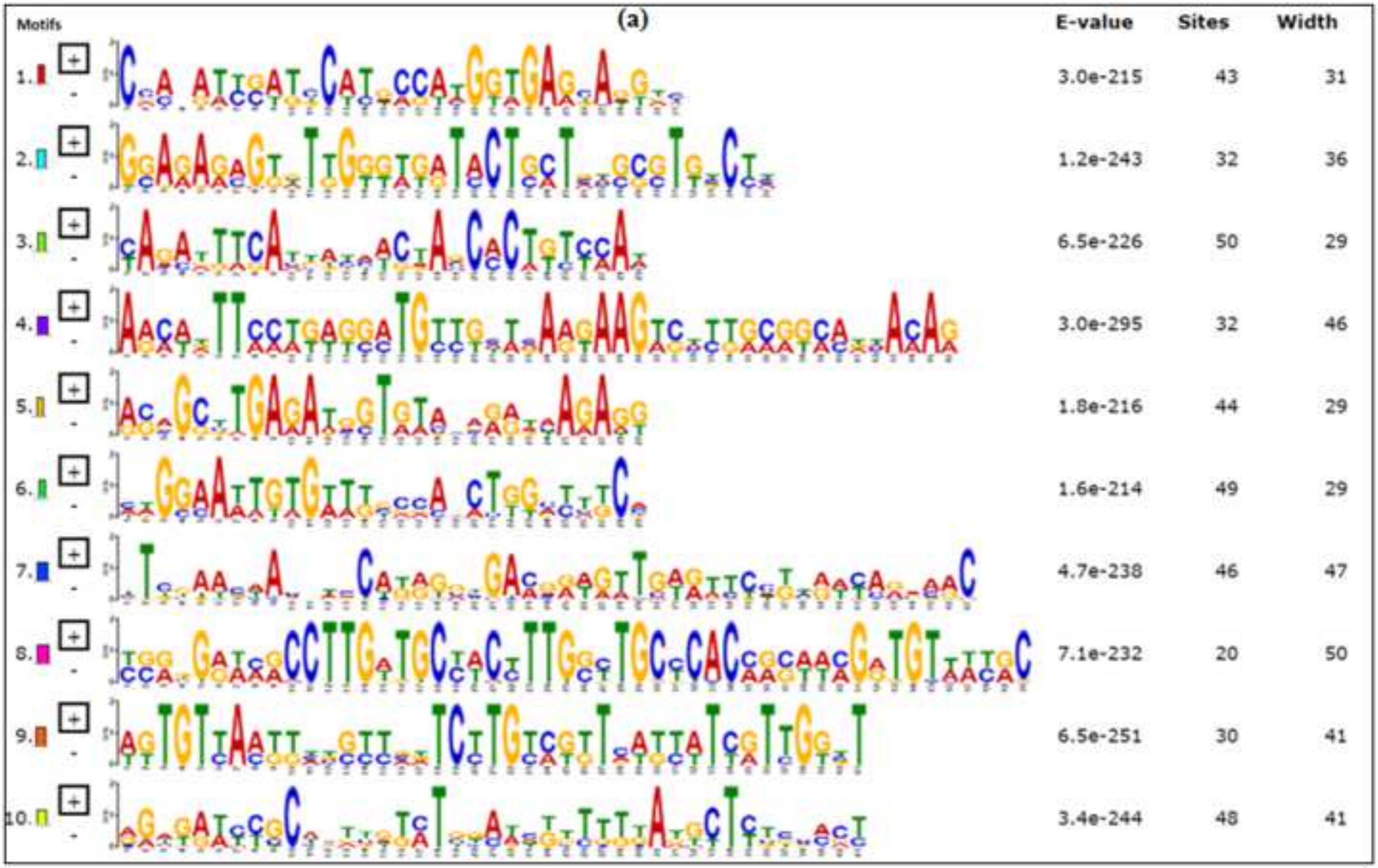

Figure 3b

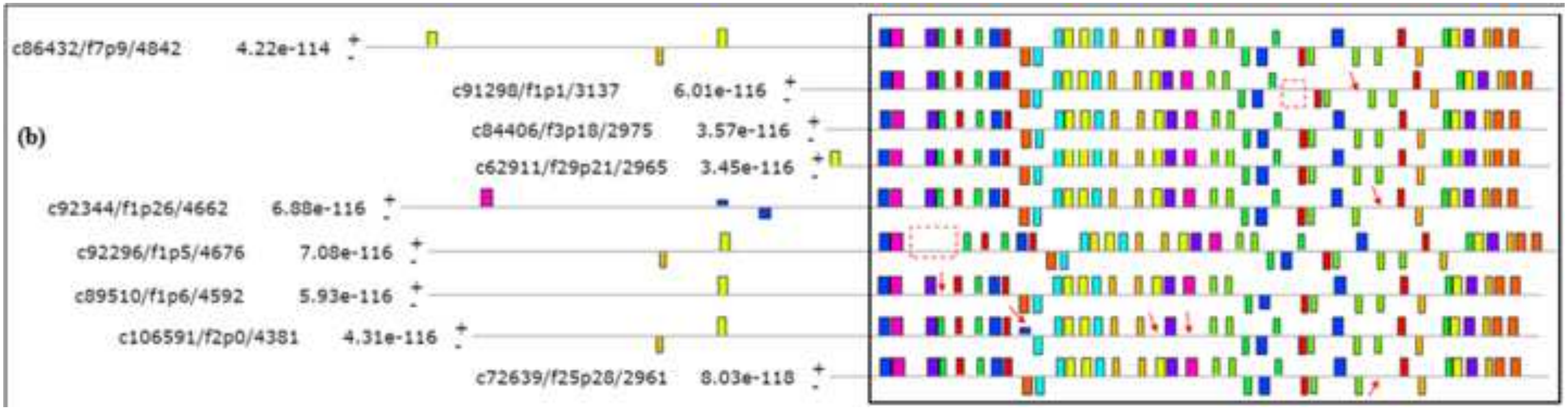
(b)
c86432/f7p9/4842 4.22e-114 + -
c91298/f1p1/3137 6.01e-116 + -
c84406/f3p18/2975 3.57e-116 + -
c62911/f29p21/2965 3.45e-116 + -
c92344/f1p26/4662 6.88e-116 + -
c92296/f1p5/4676 7.08e-116 + -
c89510/f1p6/4592 5.93e-116 + -
c106591/f2p0/4381 4.31e-116 + -
c72639/f25p28/2961 8.03e-118 + -

Click here to download Figure Fig 4a.tif

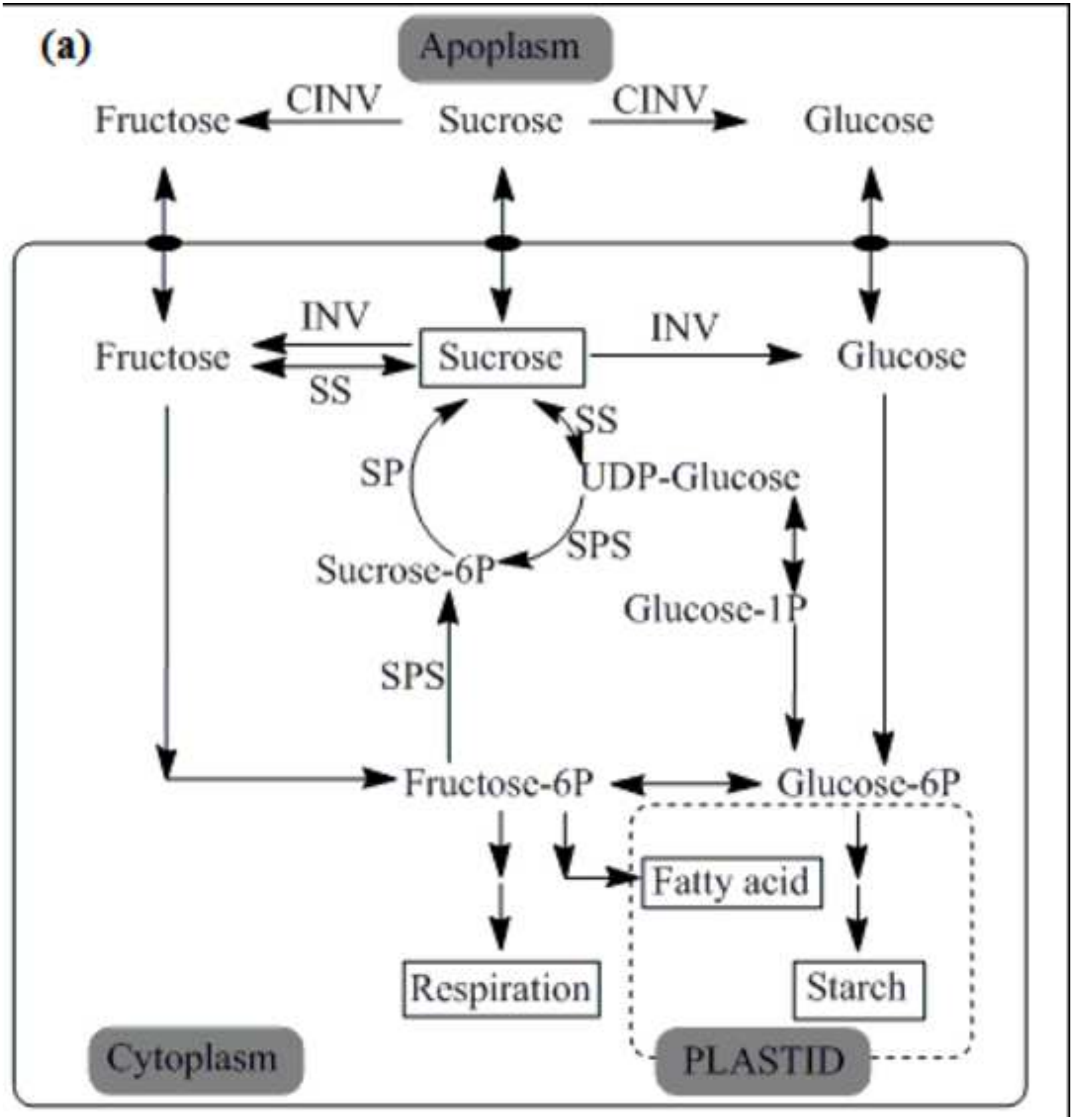

Figure 4b

Click here to download Figure Fig 4b.tif

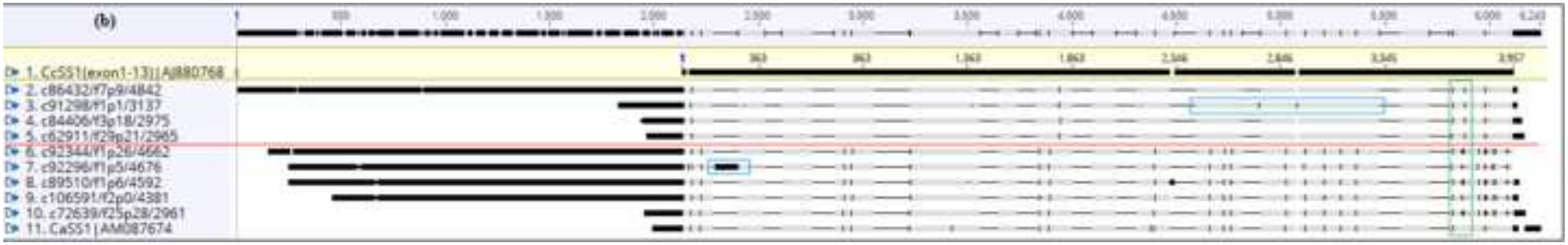

Figure 4c

Click here to download Figure Fig 4c.tif

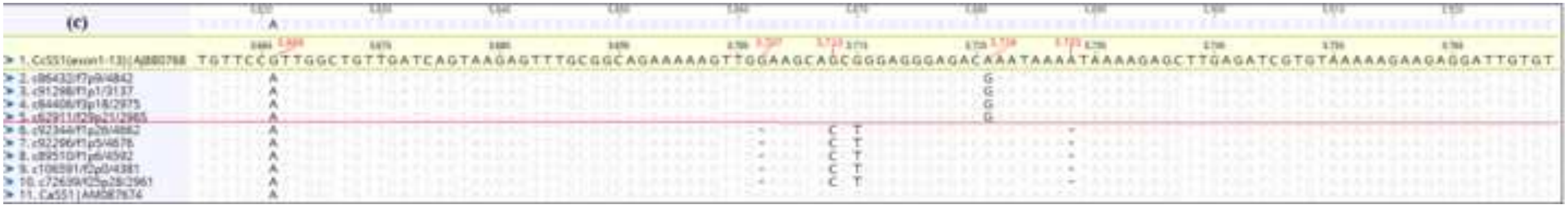

Figure 4e

Click here to download Figure Fig 4e.tif

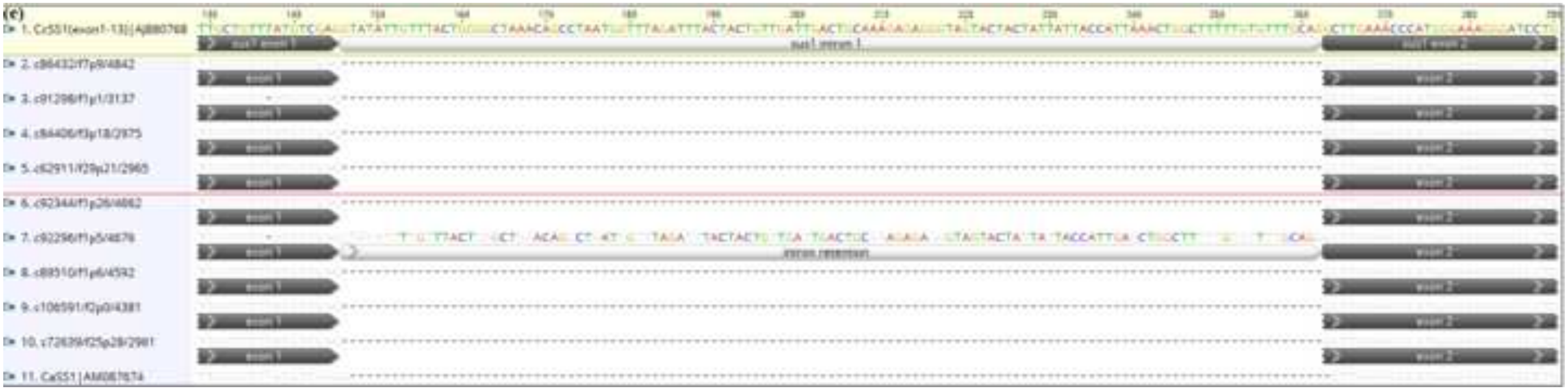

Figure 4f

Click here to download Figure Fig 4f.tif

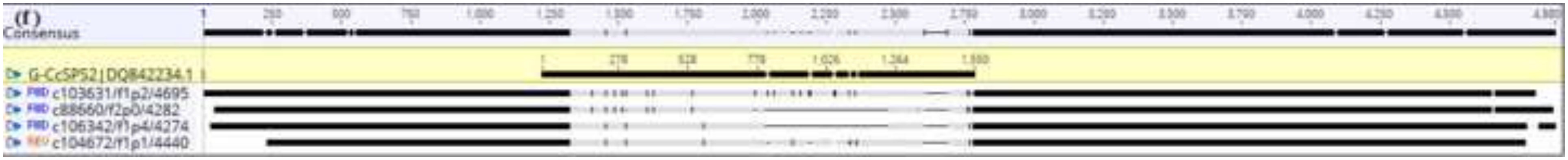

Figure 5

Click here to download Figure Fig 5.tif

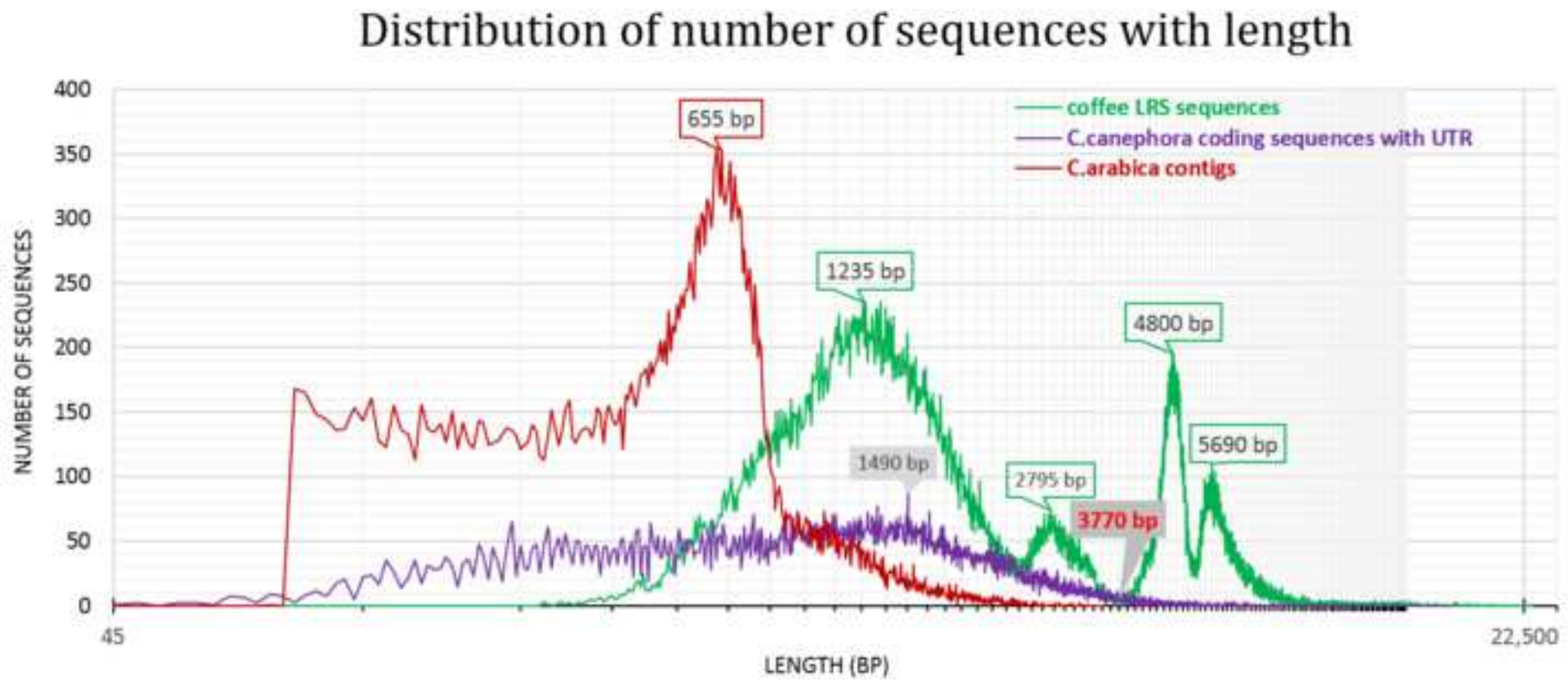

Click here to access/download
Supplementary Material
3. supporting information 1-for resubmission.pdf

Supplementary Material 2

Click here to access/download
Supplementary Material
4. supporting information 2-.xlsx
