# Supplementary material for: Long-read sequencing of the coffee bean transcriptome reveals the diversity of full-length transcripts
Source: Gigascience. 2017 Aug 30;6(11):1–13. doi: 10.1093/gigascience/gix086 (PMC5737654; doi:10.1093/gigascience/gix086)
Supplement: supporting information 1-for 3rd submission.pdf [file gix086_supporting_information_1-for_3rd_submission.pdf]

Supporting information 1

Table S1 BLAST output filtering with query coverage and cumulative identity. Qcovs (query coverage); ID (cumulative identity); NR plant, NCBI non-redundant plant protein database; NT, NCBI non-redundant nucleotide database; #sequences, number of sequences.

Table S2 Coffee long read sequencing isoforms annotated by Rfam (rfam.xfam.org).

Figure S1 Species distribution of coffee long read sequencing isoforms according to the result of BLAST against NCBI non-redundant database.

Figure S2 InterProScan families distribution of coffee long read sequencing isoforms.

Figure S3 Distribution of InterProScan families from coffee long read sequencing dataset.

Figure S4 Pie chart and word cloud of coffee long read sequencing isoforms distribution to biological process, cellular component and molecular function

Figure S5 The KEGG pathway distribution of coffee long read sequencing isoforms.

Figure S6 Putative transcript variants from long-read sequencing aligned to reported genes encoding 7-methylxanthine N-methyltransferase. G-CaMXMT1, Arabica MXMT1 genomic DNA sequence; G-CaMXMT2, Arabica MXMT2 genomic DNA sequence; MXMT1, MXMT2, MXMT1, MXMT2 coding sequence; Ca, Arabica coffee; Cc, Robusta coffee; c20397/f5p1/1361, c10402/f2p3/1277, coffee long read sequencing isoforms.

Figure S7 Pie chart and word cloud of long sequences (coffee long read sequencing isoforms >10kb) distribution to biological process, cellular component and molecular function.

Table S1 BLAST output filtering with query coverage and cumulative identity. Qcovs (query coverage); ID (cumulative identity); NR plant, NCBI non-redundant plant protein database; NT, NCBI non-redundant nucleotide database; #sequences, number of sequences.

BLASTx output (NR plant)				
**High**	300-1,000 bp	1,000-3,000 bp	3,000-5,000 bp	>5,000 bp
Qcovs (%, ≥)	60	50	40	30
ID (%, ≥)	70	70	60	60
#sequences				**34,719**
**Medium**	300-1,000 bp	1,000-3,000 bp	3,000-5,000 bp	>5,000 bp
Qcovs (%, ≥)	50	40	20	10
ID (%, ≥)	60	60	50	50
#sequences				**13,655**
**Low**	300-1,000 bp	1,000-3,000 bp	3,000-5,000 bp	>5,000 bp
Qcovs (%, ≥)	9	4	2	1
ID (%, ≥)	22.69	22.71	25.50	24.01
HSP length range (bp)	88-961	92-2,844	88-4,938	97-14,424
#sequences with hit				**40,341**
#sequences without hit to NR plant				**7,280**
BLASTn output (NT) with 7,280 sequences				
	300-1,000 bp	1,000-3,000 bp	3,000-5,000 bp	>5,000 bp
Qcovs (%, ≥)	5	2	1	1
ID (%, ≥)	73.32	71.83	75.51	76.09
HSP length range (bp)	41-962	37-1559	32-4219	50-5578
#sequences with hit				**1,981**
#sequences without hit to NR plant and NT				**5,299**
BLASTn output (C.canephora CDS with UTR and C.arabica contigs) with 5,299 sequences				
#sequences without hit				**1,217**
Putative novel genes in coffee (after filter with Rfam)				
#sequences without hit				**1,213**

Table S2 Coffee long read sequencing isoforms annotated by Rfam (rfam.xfam.org).

Sequence Name	Family Acc	Family ID	Score	E-Value	GC%	Start	End	Strand	Biotype	GOs
c63645/f1p0/1090	RF00075	mir-166	92.7	8.50E-24	0.42	298	452	+	Gene; miRNA	GO:0035068 GO:0035195
c36778/f1p0/867	RF00267	snoR64	69.7	1.50E-14	0.36	445	539	+	Gene; snRNA; snoRNA; CD-box	GO:0005730 GO:0006396
c42517/f1p0/1215	RF00360	snoZ107_R87	87.9	9.50E-20	0.49	276	384	+	Gene; snRNA; snoRNA; CD-box	GO:0005730 GO:0006396
c25730/f1p0/842	RF01227	snoR83	91.6	1.90E-18	0.45	183	321	+	Gene; snRNA; snoRNA; HACA-box	GO:0005730 GO:0006396

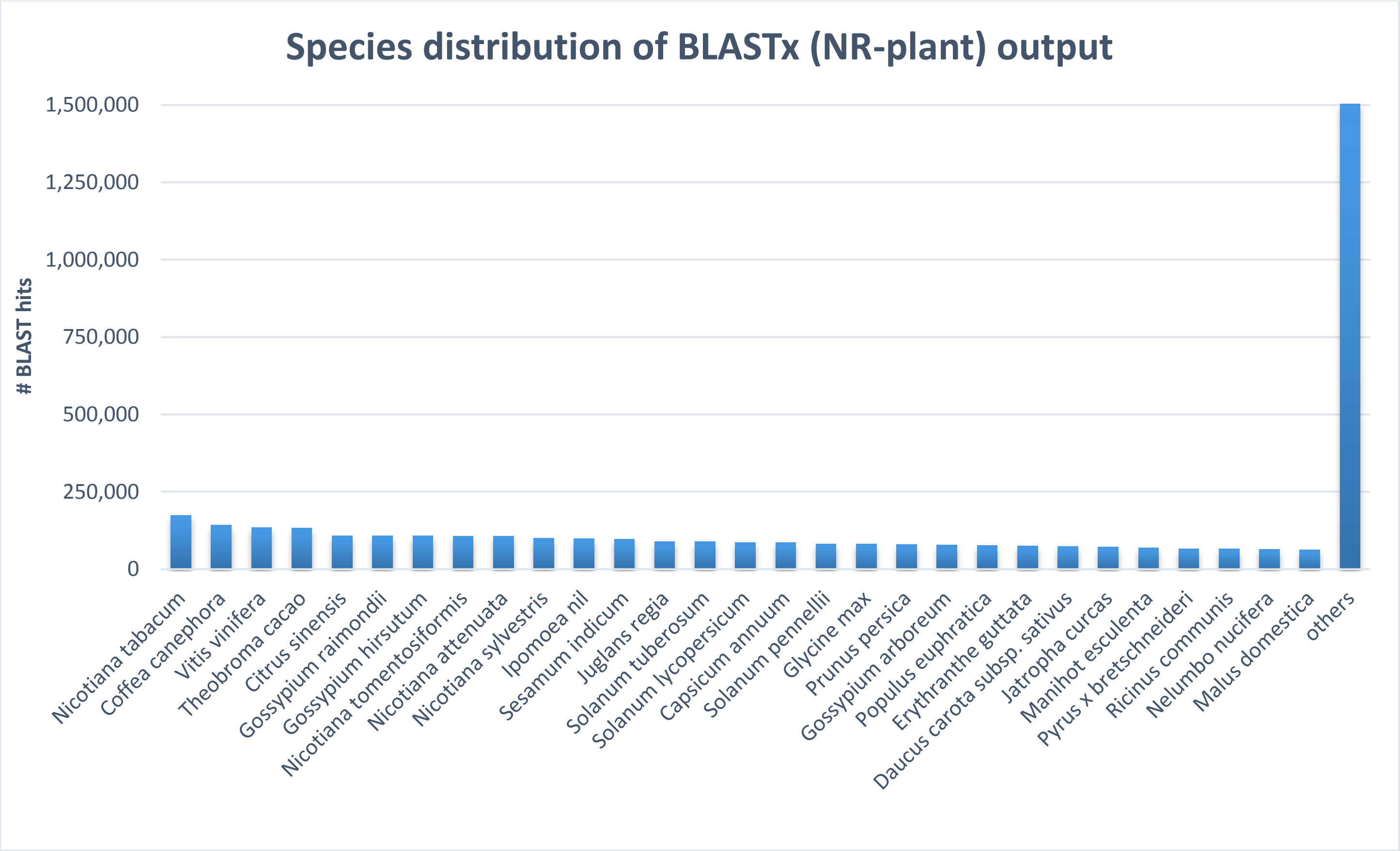

Figure S1 Species distribution of coffee long read sequencing isoforms according to the result of BLASTx against NCBI non-redundant plant proteins (NR-plant). #, number.

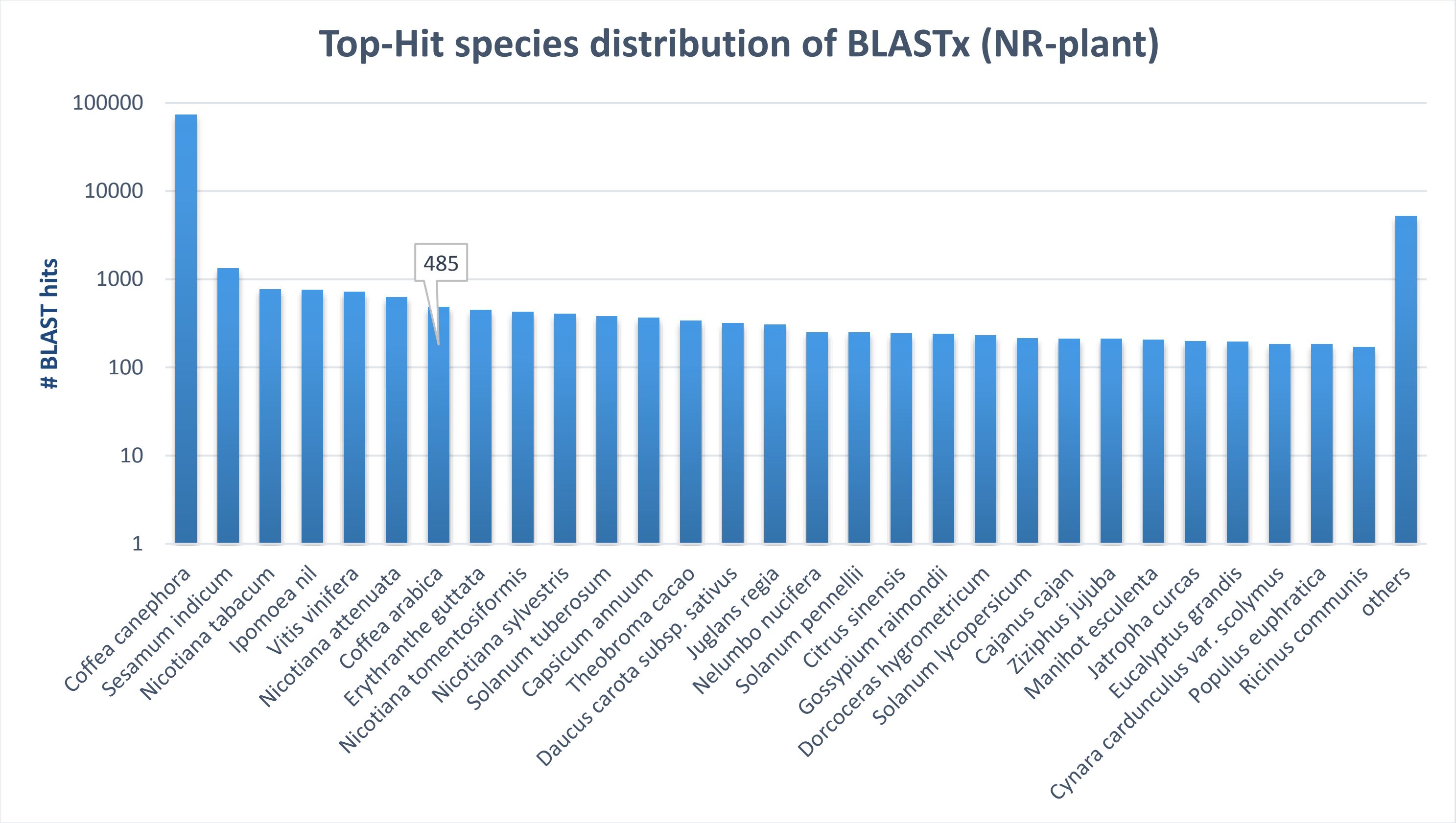

Figure S2 Top-Hit Species distribution of coffee long read sequencing isoforms according to the result of BLASTx against NCBI non-redundant plant proteins (NR-plant). #, number.

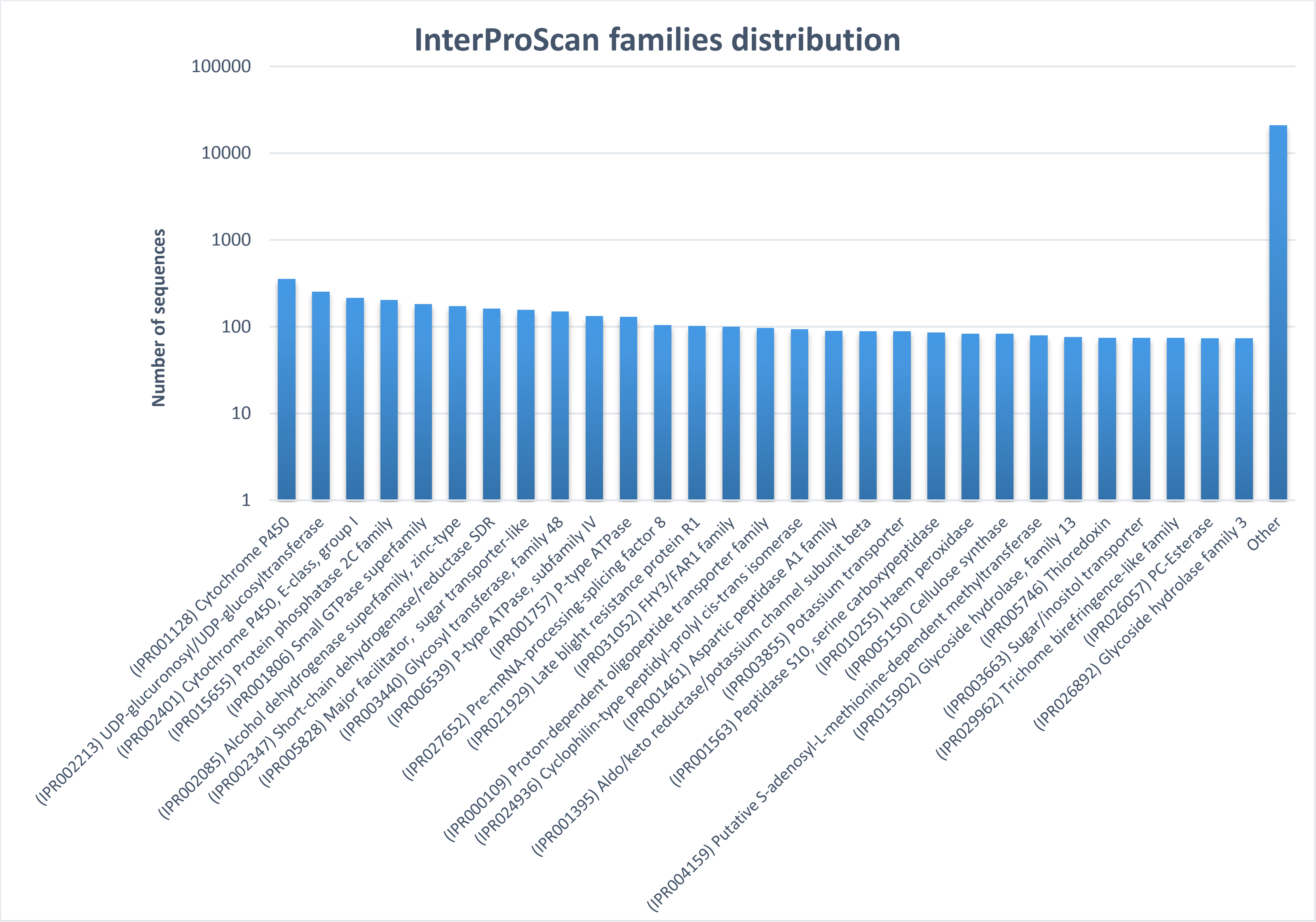

Figure S3 Distribution of InterProScan families from coffee long read sequencing dataset

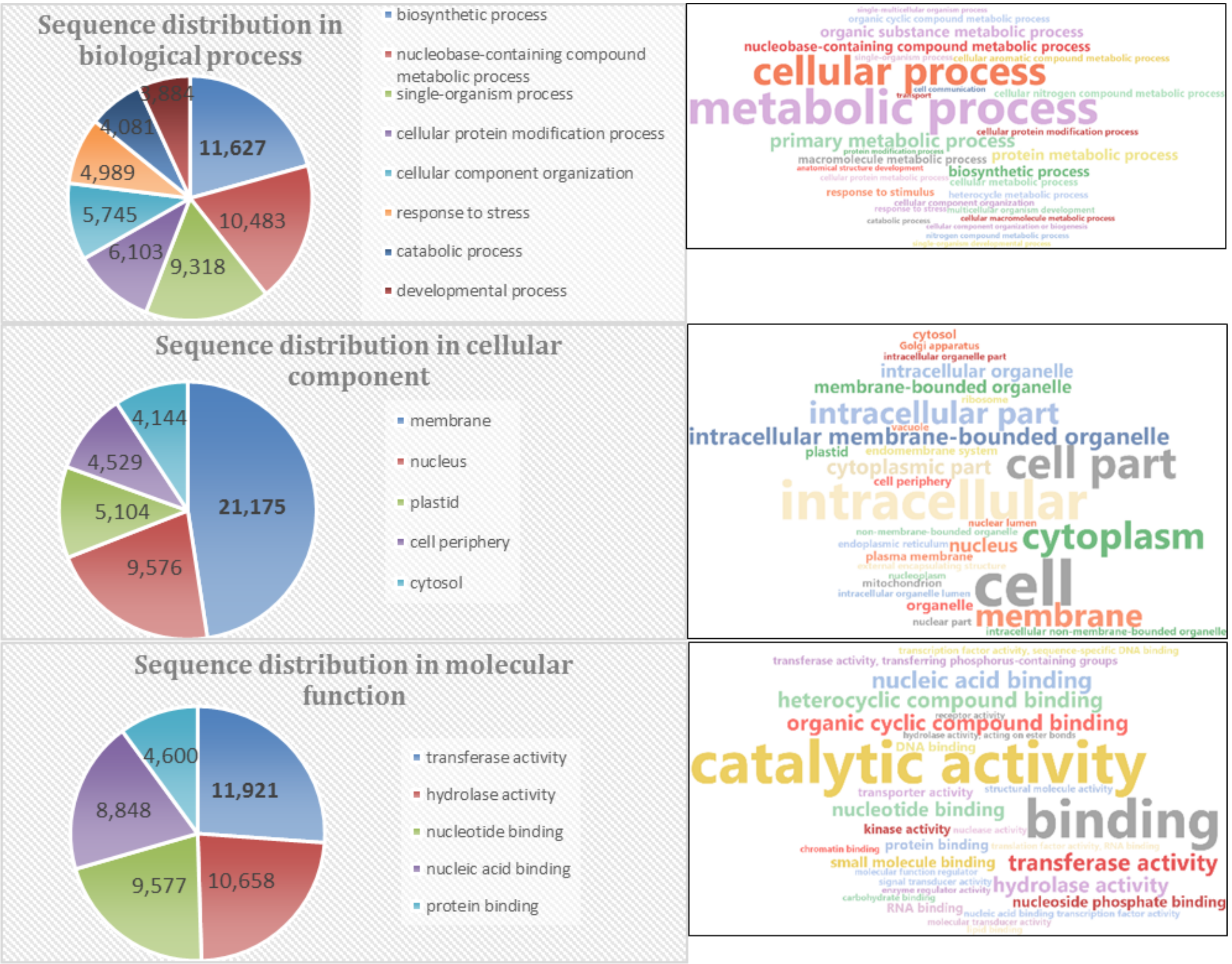

Figure S4 Pie chart and word cloud of coffee long read sequencing isoforms distribution to biological process, cellular component and molecular function

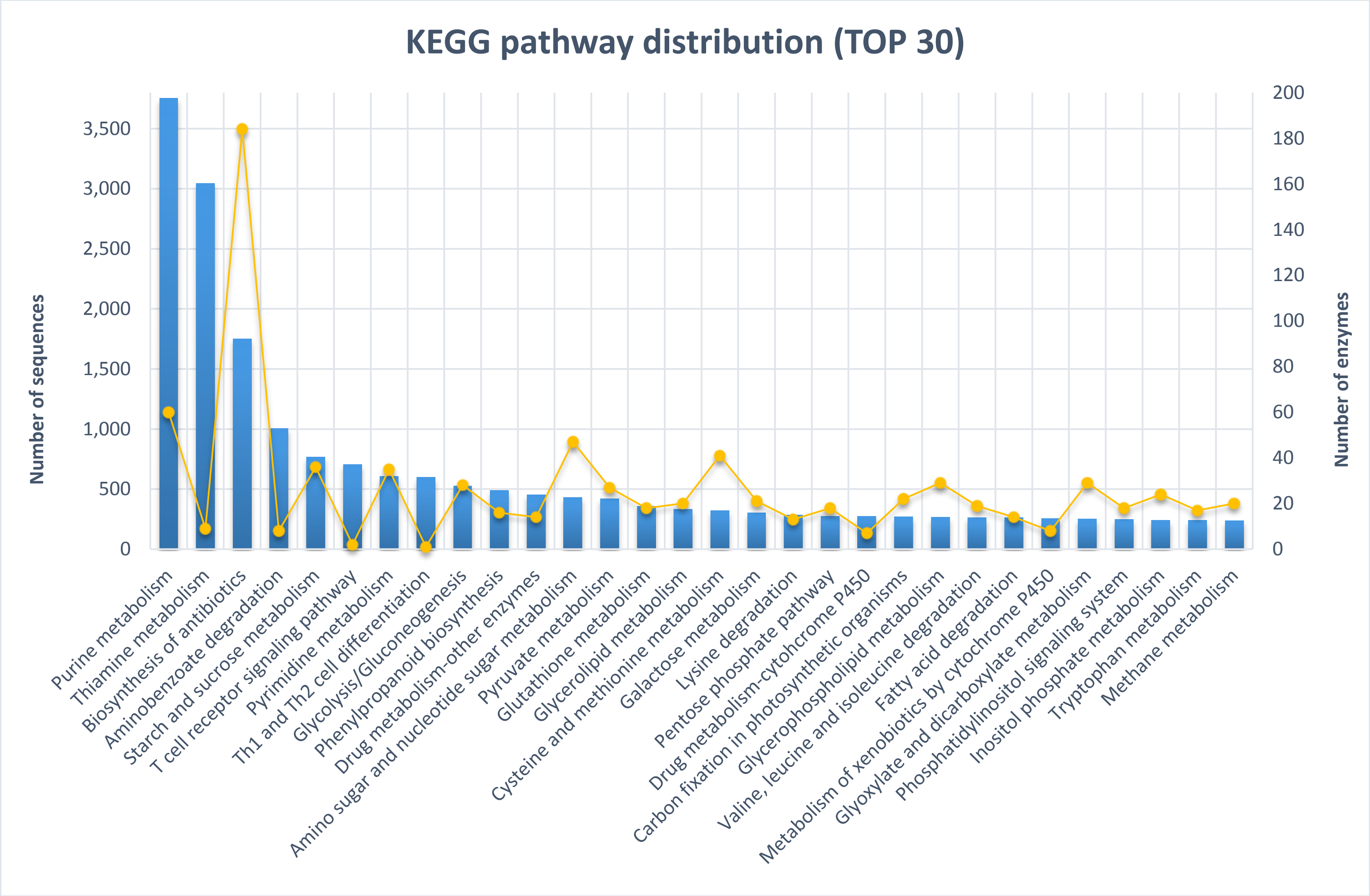

Figure S5 The KEGG pathway distribution of coffee long read sequencing isoforms.

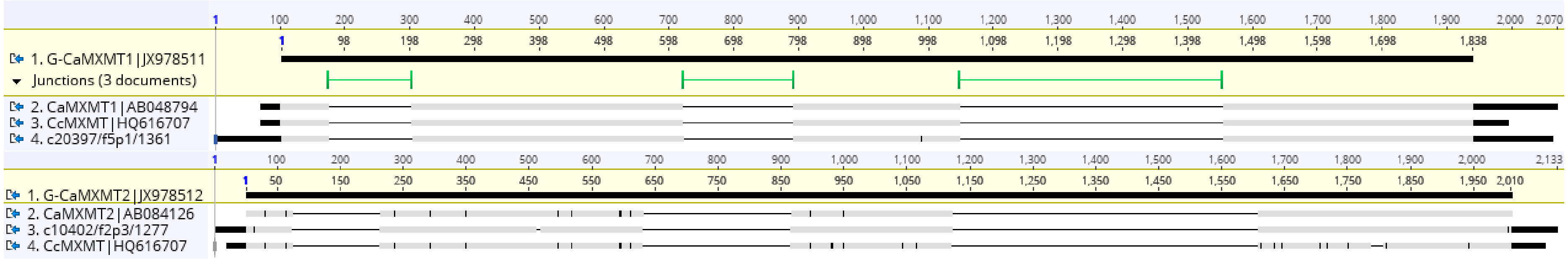

Figure S6 Putative transcript variants from long-read sequencing aligned to reported genes encoding 7-methylxanthine N-methyltransferase. G-CaMXMT1, Arabica MXMT1 genomic DNA sequence; G-CaMXMT2, Arabica MXMT2 genomic DNA sequence; MXMT1, MXMT2, MXMT1, MXMT2 coding sequence; Ca, Arabica coffee; Cc, Robusta coffee; c20397/f5p1/1361, c10402/f2p3/1277, coffee long read sequencing isoforms.

Note: Black colour in the aligned area (correspond to G-CaMXMT reference genes) indicates sequences different to the reference.

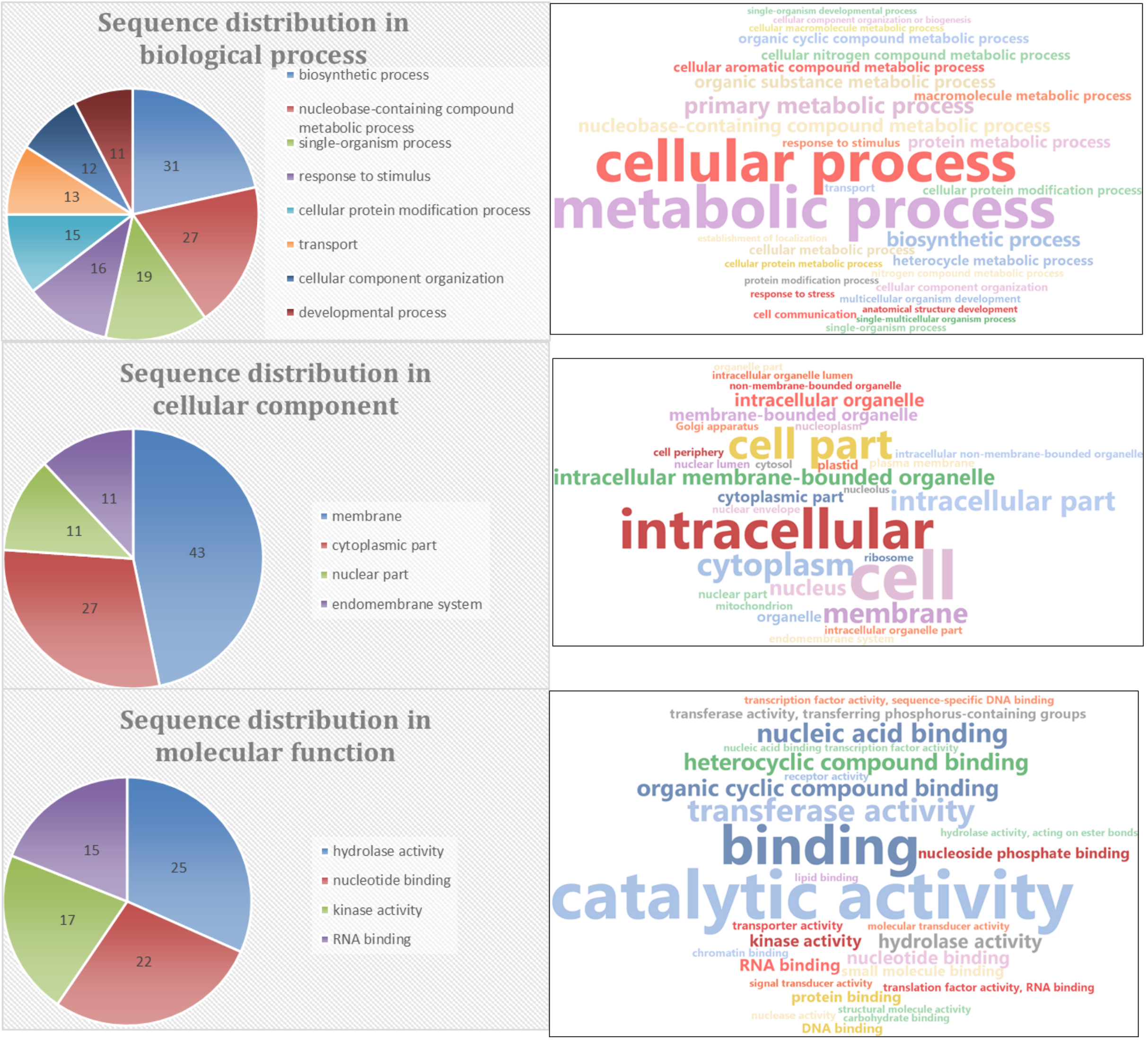

Figure S7 Pie chart and word cloud of long sequences (coffee long read sequencing isoforms >10kb) distribution to biological process, cellular component and molecular function.
