# Supplementary material for: Long-read sequencing of the coffee bean transcriptome reveals the diversity of full-length transcripts
Source: Gigascience. 2017 Aug 30;6(11):1–13. doi: 10.1093/gigascience/gix086 (PMC5737654; doi:10.1093/gigascience/gix086)
Supplement: Response-to-Reviewer-Comments_Original-Submission.pdf [file gix086_response-to-reviewer-comments_original-submission.pdf]

Author's Response To Reviewer Comments

Note:
Text following dots or dashes are responses

Reviewer 1, Sandeep Chakraborty, has validated your data and identified a couple of issues that we feel should be addressed (such as potential contaminant transcripts in the data). The reviewer also brings up a different approach to match the transcripts, which in the reviewer's opinion is more efficient.

• We re-processed the PacBio transcript sequences and those transcripts annotated to be of fungal and chloroplastic origin were removed. The number of these potential contaminants are few and insignificant in number compared to the total PacBio isoform. Those transcripts annotated to be chloroplastic may actually be nuclear insertion of chloroplast which is is widely reported. Details of the procedure has been added to "Data description" (See Page 6-7, Line 144-166).
• The database was modified as the plant protein sequences extracted from NCBI non redundant protein database (NR) and NCBI non-redundant nucleotide database (NT). Details was explicated in "Data description"(See Page 7-8, Line 173-189).
Reviewer 2, Stephanie Bocs, also makes some suggestions to improve your analyses that I hope you will consider. In particular, both reviewers mention the difficulties of using a pre-set e-value for identifying BLASTN hits.
• We reprocessed the BLAST analysis and reduced the BLASTx and BLASTn e-value to 1e-10 and 1e-20 (see Page 8, Line 177, 179, 180 of "Data description")
• We have reprocessed the data with additional parameters such as "query coverage", "cumulative identity" and ""alignment length" to filter the BLAST output based on the length of the sequences. Details of this analysis is analysis is added (Page 8, Line 181-187) to "Data description" and supporting information Table S1.
Please also note the attached .doc file, which includes an annotated version of your manuscript with further comments by reviewer 2 (you may need to turn "view comments" on in MS Word to see the commentaries).

I feel that the two reports include many constructive comments and I hope the reports will help you to further improve your manuscript.

If you are able to address these points, we encourage you to submit a revised manuscript to GigaScience. Once you have made the necessary corrections, please submit online at:

http://giga.edmgr.com/

If you have forgotten your username or password please use the "Send Login Details" link to get your login information. For security reasons, your password will be reset.

Please include a point-by-point within the 'Response to Reviewers' box in the submission system. Please ensure you describe additional experiments that were carried out and include a detailed rebuttal of any criticisms or requested revisions that you disagreed with. Please also ensure that

your revised manuscript conforms to the journal style, which can be found in the Instructions for Authors on the journal homepage.

The manuscript format is now modified to conform to the journal style and outlined in the
The due date for submitting the revised version of your article is 02 Jul 2017.

I look forward to receiving your revised manuscript soon.

Best wishes,

Hans Zauner
GigaScience
www.gigasciencejournal.com

Reviewer reports:
Reviewer #1: Cheng et al. have presented a manuscript on a very relevant topic - third generation sequencing on an economically important crop. While I wholeheartedly agree that long read sequencing will address several assembly, and downstream, problems - resulting in a better understanding of several genetic aspects of any organism, there are several inaccuracies in the current manuscript that need to be addressed before
publication.
1. There are several transcripts (about 40) from the pathogenic fungus genus Fusarium - C117579.F1P0.6198 is one such example. Methods for quickly detecting metagenomic transcripts have been elucidated in http://biorxiv.org/content/early/2016/10/04/079186. https://www.ncbi.nlm.nih.gov/pmc/articles/PMC1317314/ is just one of previous work on this pathogens effect on the coffee yield.

- We re-processed the PacBio transcript sequences and those transcripts annotated to be of fungal origin were removed. The number of these potential contaminants are few and insignificant in number compared to the total PacBio isoform. Those transcripts annotated to be chloroplastic may actually be nuclear insertion of chloroplast which is widely reported. Details of the procedure has been added to “Data description” (See Page 6-7, Line 144-166).
2. "In total, 96,415 coffee-LRS isoform sequences were recovered after sequences representing chloroplast, mitochondrial and ribosomal transcripts" - chloroplastic sequences have not been removed completely. C118772.F1P0.4602 is one example. There are about 20 such transcripts.

- We have re-processed our data to remove contaminating sequences as described on Page 6-7, Line 144-166. In brief, contaminants from chloroplast, mitochondrial, ribosome, fungi, bacterial, virus and viroids were removed. A BLASTn (1e-10) was performed against the Arabica chloroplast genome, tobacco and grape mitochondrial genome, C.arabica and its progenitor ribosomal genes reported, fungi proteins (BLASTx), bacterial, viral and viroids reference genomes downloaded from NCBI. The BLASTn/BLASTn output was filtered with bit score (A, ≥300 bits) and great identity (≥80%). Filtered sequences were further BLASTn (1E-10) to NT database (bit score: B) to confirm based on higher bit score whether these sequences are contaminants (A>B) or chloroplast insertions/conserved sequences to nucleus (A
3. "After filtering LRS isoform sequences with NCBI-nr, 5,667 sequences without a hit were" -

in the absence of a reference genome, absence in the NCBI-nr database cannot be inferred as novel genes. It might be contamination from an hitherto un-sequenced organism. If novel does not imply novel in coffee, this needs to be clearly stated.

- We have now defined the meaning of the words “Novel genes” assigned to the novel genes in coffee or possible contaminating sequences from other organisms. Please see Page 16, Line 381-382.

Matching the 96k transcripts to ncbi-nr is grossly inefficient (and inaccurate, as observed with the matches to the fungus). I have validated the provided data, with emphasis on both accuracy and computational times. Most of the analysis done here is on a small workstation (8GB ram) within a day. The transcript names have been changed to replace "/" with a "." to allow for Unix style file names. In the search for novel genes and annotation started with the cds from related genomes (coffee-genome.org/), and followed by transcripts from related plant genomes (about 8 - vitis, malus, sesame etc). This quickly identified homologous genes - thus reducing computational times significantly as compared to matching to ncbi-nr. The unmatched genes was reduced to about 1000 from 96k in a few hours.

• We agree that BLASTx to the whole NR database is not sufficient. Therefore, the plant nucleotide sequences (5,099,147 sequences from) were extracted from the NR database (May 2nd, 2017, 121,684,710 sequences). This reduces the running time remarkably and avoids matching to non-plant organisms. Please see Page 8, Line 173-189.

• Eight related genomes (mainly diploid) cannot represent all the Arabica sequences (complex tetraploid) For novel/un-sequenced gene detection, further BLASTn to a broader NT database was conducted with the sequences that had no hit to NR plant proteins. There are 42,576,813 sequences in this dataset, including genomic sequences, non-coding sequences, etc. As the number of sequences without hit was small (7,208), BLASTn to this database was more comprehensive and will be efficient. In addition, C.canephora coding sequences and UTR as well as C.arabica EST database were used for BLASTn for further remove sequences with hits to current databases. (see Page 8, Line 178-181 and Page 10, Line 218-221).

In my opinion, 1E-05 is too high a value for significance for nucleotide matching. The exact threshold is can be debated till the end of the world. One example is C107709.F1P0.5231 (Length=5231) matching to XM 010247806.2 (Length=8430) from lotus with Evalue=4e-10. The alignment seems too small to be considered significant.
AAACCGTTTTCATCACTTCAAAGATGGATTTTGTTCTTGTGGAGATTAT-
GGTAAATCGATTTCATCACTTCAAAGATGGATTTTGTTCTTGTGGAGATTATTGGT
One possible way to address this might be to split matches into low, medium and high significance.

• The E-value has been reduced to 1e-10. The output from BLASTx/BLASTn was classified into three catogories, high, medium and low as suggested (see supporting information Table S1).

• Additional parameters such as query coverage (Qcovs) and cumulative identity (ID, calculated based on alignment length (AL) and sequence length, were selected to filter the BLASTx output. Query coverage indicates the input coffee-LRS sequences covered by the matched sequences (reported by BLASTx output format type 6). Cumulative identity (ID) represents the identity length the aligned length (AL). ID can be expressed as the ratio of the sum of identity length to the sum of aligned length of all the Hsps (High-scoring Segment Pairs) of a subject. This has

been described in “Data description” (Page 8, Line 181-187).
The protein section for xmt genes (Table 1) is accurate. However, the absence of an xmt2 gene needs to be discussed since there is a given gene for coffea arabica, with which there is a lower (but still significant) homology in C30813.F1P0.1617. The reason for this needs to be discussed.
• Previously caffeine candidate genes were filtered with BLASTn (bit score: C) of the coffee LRS isoforms to reported caffeine genes in coffee. We have re-processed our data and added additional test. A further BLASTn (bit score: D) of the sequences (with hits to reported caffeine genes) to NT database was applied to check whether they are true caffeine sequences (C>D) or homeologs to other relate sequences (C
• We do not agree with gene C30813.F1P0.1617 encoding XMT2, as higher bit score was showed to the gene (MLT) encoding mythyltransferase-like protein and this transcript is not aligned properly (numerous variants) with genomic XMT2 (the only coffee XMT2 gene reported) from Arabica coffee by Geneious software. The XMT2 is not identified in this dataset and this has been described in the manuscript (Page 12-13, Line 290-292 and Table 3). This is probably because XMT2 was expressed at a very low level in coffee bean, even though normalization had been done in cDNA library preparation to capture the rare genes. This is consistent with previous study that XMT2 was expressed at relatively low levels in coffee bean (detected by quantitive RT-PCR) Charlene Perrois (2015).
Minor comments. There are several grammatical and typographical errors. I am mentioning the first three.
1. Abstract: uncertain should be uncertainty.
- Corrected in Page 2, Line 34, Page 3
2. Abstract: frams - frames.
- This word has been deleted as the abstract has been revised according to the updated results.
3. Abstract: toolto - tool to
- Corrected in Page 2, Line 47.
Reviewer #2: This manuscript reports a long-read sequencing of coffee bean transcriptomes.

This resource provides a reference transcriptome for the community working on the genetic improvement of the coffee tree. The study is interesting because it covers multiple aspects: caffeine and sucrose biosynthesis pathways, long and rare transcripts, novel genes, ORFs in 5'UTR. I encourage the authors to go a step further in their analyses to better promote their work.
*Major comments
1) In the background part or in the result part, I recommend defining terms important for this study.
a) For instance, regarding the term of 'transcript isoform', if I understood well the point of view of the authors in the context of polyploid species, transcript isoforms of a gene should represent splice variants (alternative transcripts), alleles, homoeologs but not close paralogs (Childs et al. 2014). They can also have a look at this publication (Gutierrez-Gonzalez et al. 2013) where Gutierrez et al. defined the terms of 'transcript isoform' or 'transcript', 'locus' or 'loci', 'splice variants', 'homoalleles'
- Usage of the transcriptome is very diverse and specific terms were defined as follows, see Page 7, Line 168-171.
- The term ‘isoforms’, or ‘isoform sequence’ or ‘transcript’ used in this study represents (generic) individual sequences from the coffee-LRS dataset. It is commonly used in Pacbio Isoform sequencing, as full length sequences were captured but need further information to

confirm whether an individual sequence is a spliced variant to some other sequences (from the same dataset) or it is the only one sequence without spliced variants. In addition, transcript variants was introduced in this manuscript, indicating different variants of a gene, including alternative spliced variants, homeologs, etc.

b) Also, I found that some important terms for the evolutionary process of plant polyploidization are missing. For instance, I would better understand the sentences "Diversification or specialisation may alter the nature of the gene product (e.g. encoded protein sequence) or the pattern of expression (e.g. tissue specificity of expression) of genes from each subgenome. Moreover, the copy number of genes in each sub-genome may be altered or the gene may even be deleted completely from some sub-genomes" if the authors add the terms of subfunctionalization, neofunctionalization and pseudogeneization (Levasseur and Pontarotti 2011).

- This was modified in the manuscript to "Genetic changes associated with the formation of polyploids include gene function, which may remain unchanged, or diversify among the multiple homeologs, leading to neofunctionalization, subfunctionalization, or pseudogenization." in Page 3, Line 59.

2) Most of the methods are appropriate to the aims of the study but three points should be improved.

a) To identify the query sequences having a BLASTN hit, the e-value is not satisfying parameter because it depends on the size of the databank and thus is not comparable between several BLAST analyses. Instead, other parameters could be used such as AL, CIP and CALP (Salse et al. 2008) or the identity percentage, qcov and scov (Mbeguie et al. 2009). They will allow filtering results more precisely and independently of the databank.

- As suggested, we have reprocessed the data and applied additional parameters such as AL, Qcovs and ID, as outlined above. The amended BLAST analysis steps are indicated in the "Data Description" section on Page 8, Line 181-187.

b) A GMAP (Wu and Watanabe 2005) analysis of the coffee LRS transcriptome against the C. canephora genome could help to (i) separate homeologs from paralogs for each locus, (ii) detect missing genes in C. canephora annotation (see minor suggestion 4) and (iii) define clusters that could then be refined to separate the homeologs according to their origin (see major comment 3). The authors can have a look for instance on Polycat (Page et al. 2013) and Homeosplitter (Ranwez et al. 2013) methodologies developed for allotetraploid crops.

- The paternal genome of Arabica coffee, C. canephora genome is only a draft genome yet to be completed while the maternal genome, C. eugenioides, is not available to date. Therefore, homeolog prediction undertaken using the incomplete C. canephora genome can lead to inaccuracies in assigning homeologs especially in the absence of the paternal genome sequence data. Hence, the coffee-LRS reference in its current form can be used by researchers for a range of analysis. The analysis suggested can be undertaken in future when these two progenitor genome are complete.

c) For coding sequence analysis, Framedp (Gouzy et al. 2009) or prot4EST (Wasmuth and Blaxter 2004) would be more adapted than the ORF prediction.

- The ORF prediction was removed. This is a transcriptome dataset so each sequence is supposed to be a full length sequence, with or without coding potential, unless they are fusion genes. ORF is normally predicted in genomic data rather than transcriptome. Moreover, the ORF predicted are not show high confidence as the software used predict every possible ORFs with a start codon ATG, etc. This means even uORFs have the potential to be predicted as ORFs. The

software introduced, such as Framedp, generate ORFs based on aligning the sequences to homeolog sequences public databases (similar to BLAST). However, in this dataset, ORFs were predicted for novel sequences in coffee (no hit to NR, NT and coffee databases) and long sequences (>10kb). There is limited information from the public databases (see “Analysis”), therefore, it is not worth report unreliable results from this.

3) The data produced should be sufficient to support the discussion. The authors claim that "This study clearly shows the expression of sub-genome copies accounting for much of the polyploid diversity" but the analysis is not thorough enough. The reader would expect to have access to at least a set of sub-genome specific isoforms (i.e. couples of transcript isoforms for each locus tagged as derived from C. canephora or from C. eugenioides). Indeed, it could be too difficult to phase the four haplotypes of this allotetraploid LRS transcriptome if the two haplotypes of each sub-genome are very close. I do not know if PacBio Iso-Seq™ community could help the authors for this analysis but maybe the HapIso methodology (Mangul et al. 2016) could be used making the assumption that the study of an allotetraploid can be reduced to the study of a diploid. So a 2-means clustering should be sufficient even if a 4-means could also be tested.

• The sentence in question has now been amended to “Case studies of sucrose genes clearly shows the expression of sub-genome copies accounting for much of the polyploid diversity.” (Page 18, Line 413-414)

• This comment “expect to have access to at least a set of sub-genome specific isoforms” is similar to the above 2b. The result can only be more accurate with complete genome.

Instead, caffeine and sucrose genes were performed as case studies to understand specifically the quality, diversity and the potential of this coffee-LRS dataset, which gave clues to the UTR analysis. The caffeine biosynthesis pathway used in this study as it is one of the most important pathway in coffee. The sucrose biosynthesis pathway was used because its genes are very long (e.g. SS1 is 2,979 kb) and the current short-sequencing cannot capture their full length. Analysis of sucrose genes will improve understanding of the potential of long sequencing technologies, for example, discovery of the full-length genes, more transcript variants, longer 5’UTRs, etc.
****

*Minor suggestions

1) Replace 'cds' with 'CDS'

- Agreed and changed in Page 8 Line 181, and also applied in the whole manuscript.

2) Replace 'eg' with 'e.g.'

- Agreed and changed in Page 3, Line 73, and also applied in the whole manuscript.

3) Under ten (1, 2, 3, 4, 5, 6, 7, 8, 9 and 10), write number in letters (one, two, three, four, five, six, seven, height, nine, ten).

- Agreed and changed in Page 12 Line 289-302, and also applied in the whole manuscript.

4) The sentence "Most sequences matched tobacco probably because the tobacco database is more extensive and well annotated than those of other related species, like C. canephora." is not at the most suitable location in the text because it talks about tobacco in paragraph about Coffee database.

- Agreed and moved in Page 11 Line 254-255.

Also, the speculation about the annotation quality of C. canephora is not well supported. The GMAP analysis, suggested in the major point 2c, could also allow estimating the number of missing genes in the C. canephora annotation.

- Not agree as discussed above in Point 2b.

5) Each time, precise the kind of BLAST analysis, e.g. BLASTN.
- Agreed and modified in Page 8 Line 177, and also applied in the whole manuscript.
6) The figure 4f should be removed as it the same as figure 5.
- We agree and have removed figure 5.
7) See other minor suggestions directly in the text of the attached file.

Responses to reviewer's comments from the manuscript:
The reviewer's comments are in BLACK colour highlighted with "". Reply to the reviewer's comments are in BLUE colour.

- "A, e.g. doi:10.1038/nature13291". >>>Agree and Reference ([1]) has been added in Page 3 Line 55.
- Agree and corrected homologues to homeologs in Page 2 Line 58, and also applied in the whole manuscript.
- "Please see major comment 1b." >>> Please see rely to major comments 1b
- "I think there is a problem with this reference." >>> Agree and modified the reference [15] in Page 4 Line 83.
- "A reference would be useful, e.g. https://github.com/PacificBiosciences/IsoSeq_SA3nUP/wiki">>> Agree and a reference [17] was added for RS IsoSeq (version 2.3) pipeline in Page Page 6 Line 125.
- "May be better placed in the references?" Agree and modified website link as reference ([17]) in Page 6 Line 125.
- Agree "annotation augmentation" is confused and modified as "increase the number of annotated isoforms" in Page 8 Line 194-195.
- "A reference is missing." >>>Agree and reference [24] has added in Page 9 Line 210.
- "The coffee genome hub reference could be added PMID:25392413." >>> The coffee genome hub reference was added (reference [21]) in Page 8 Line 180-181.
- "I do not understand how the lncRNA were predicted. Could the authors precise it?" >>> Please see reply to review's comment 2c.
- "Why this threshold? Why not 150 bp for instance?" >>> Short sequences less than 300 bp were removed as the Bluepippin cDNA size selection starts from 500 bp, where some sequences less than 500 bp have chances to be sequenced. See Page 6 Line 127-128.
- "Is it a sequencing depth of 5X? How was it compute?" >>> Average passes of individual sequences were five. See Page 10 Line 230-231.
- "Please see Minor suggestion 4." >>> Please see reply to minor suggestion 4.
- "If you used instead of the E-value threshold, parameters suggested in the major comment 2a, these results should be updated." >>> Please see reply to Major suggestion 2a. The results have been updated in "Analysis (Page 10-12, Line 227-277, supporting information 1 Table S2, Figure S1-S5, Figure S7 and supporting information 2) "
- "At the moment I find that this study suggests (see major comment 3)". Please see reply to reviewer's comments 3.
- "In this study, the authors did not predict the homeologous copies for the XMT and DXMT genes, why?" >>> This has been amend in "Analysis" (Page 13, 303-309).
- "I do not find that 89.3% of BLASTX hit is a low level of annotation. About which step of annotation the authors are they talking about?" >>> This text means a low coverage of the BLASTx matched sequences to long sequences (10kb) rather than the percentage of sequences found hits from BLASTx. This has been amended in the manuscript according to the updated

results (Page 16-17, Line 388-394 and Page 21, Line 491).
• "Same as above: About which step of annotation the authors are they talking about?" >>> Please see the above comments and this has been amended in the manuscript according to the updated results (Page 22, Line 530-532).

Reference

Childs KL, Nandety A, Hirsch CN, Góngora-Castillo E, Schmutz J, Kaeppler SM, Casler MD, Buell CR. 2014. Generation of Transcript Assemblies and Identification of Single Nucleotide Polymorphisms from Seven Lowland and Upland Cultivars of Switchgrass. The Plant Genome 7:2.

Gouzy J, Carrere S, Schiex T. 2009. FrameDP: sensitive peptide detection on noisy matured sequences. Bioinformatics 25: 670-671.

Gutierrez-Gonzalez JJ, Tu ZJ, Garvin DF. 2013. Analysis and annotation of the hexaploid oat seed transcriptome. BMC Genomics 14: 471.

Levasseur A, Pontarotti P. 2011. The role of duplications in the evolution of genomes highlights the need for evolutionary-based approaches in comparative genomics. Biol Direct 6: 11.

Mangul S, Yang HT, Hormozdiari F, Tseng E, Zelikovsky A, Eskin A. 2016. HapIso : An Accurate Method for the Haplotype-Specific Isoforms Reconstruction from Long Single-Molecule Reads. CSH BioRXiv https://doi.org/10.1101/050906.

Mbeguie AMD, Hubert O, Baurens FC, Matsumoto T, Chillet M, Fils-Lycaon B, Bocs S. 2009. Expression patterns of cell wall-modifying genes from banana during fruit ripening and in relationship with finger drop. J Exp Bot 60: 2021-2034.

Page JT, Gingle AR, Udall JA. 2013. PolyCat: a resource for genome categorization of sequencing reads from allopolyploid organisms. G3 3: 517-525.

Ranwez V, Holtz Y, Sarah G, Ardisson M, Santoni S, Glemin S, Tavaud-Pirra M, David J. 2013. Disentangling homeologous contigs in allo-tetraploid assembly: application to durum wheat. BMC Bioinformatics 14 Suppl 15: S15.

Salse J, Bolot S, Throude M, Jouffe V, Piegu B, Quraishi UM, Calcagno T, Cooke R, Delseny M, Feuillet C. 2008. Identification and Characterization of Shared Duplications between Rice and Wheat Provide New Insight into Grass Genome Evolution. Plant Cell 20: 11-24.

Wasmuth JD, Blaxter ML. 2004. prot4EST: translating expressed sequence tags from neglected genomes. BMC Bioinformatics 5: 187.

Wu TD, Watanabe CK. 2005. GMAP: a genomic mapping and alignment program for mRNA and EST sequences. Bioinformatics 21: 1859-1875.
