# Supplementary material for: Long-read sequencing of the coffee bean transcriptome reveals the diversity of full-length transcripts
Source: Gigascience. 2017 Aug 30;6(11):1–13. doi: 10.1093/gigascience/gix086 (PMC5737654; doi:10.1093/gigascience/gix086)
Supplement: Response-to-Reviewer-Comments_Revision-1.pdf [file gix086_response-to-reviewer-comments_revision-1.pdf]

Author's Response To Reviewer Comments

Please note: The point by point response to reviewers is as follows.
Reviewer reports:
Reviewer #1: Cheng, et al. have satisfactorily responded to my comments with respect to contamination and xmt2 gene (my mistake).

However, significant discrepancies in their data still exist - and need to be addressed.

1) The number 1217 of "novel genes" in the paper, and those provided in 7_1271_novel_genes.fa (n=1271) dont match.
Maybe a typo (71 becomes 17?).
The revised manuscript doesn’t report the 1,271 novel sequences as this number has changed due to a small number of contaminant sequences being removed.

2) There are transcripts with really small ORFS - these cannot be taken as genes without proof
C35828.F1P0.435
MKLGFLGKGFGLKTEDERQKMKKQRRGC
C14734.F1P0.431
MMTKSPLHLYVARFYTNYSTLETSTPS
C23681.F3P0.964
MGMNMIYMDFVEGKGFLVEAKWTAFSSPE

There should be a reasonable cutoff - maybe 60.

The ORF prediction was removed in the current manuscript in the first major revision.

3) Finally, and most importantly, they are not novel : C57465.F1P0.755 ORF 2 matches to:
https://www.ncbi.nlm.nih.gov/protein/661881444?report=genbank&log$=prottop&blast_rank=1&RID=MFJCCGRX016
with a 100% match.
"The 1,217 sequences without hits to the FOUR databases (NR plant proteins" is therefore incorrect.

Please see the above comment.

4) Also, the 145 sequences in the "6_Long_non-coding_RNAs.fasta" all have long ORF's (>100).
c14217_f2p0_996_1 also an ORF with significant match (1E-25) to XP_019192529.1 (PREDICTED: uncharacterized protein C6G9.01c-like [Ipomoea nil])

Most of the "novel genes" seem to be long non-coding RNA.

Please see the above comment.

Reviewer #2: I greatly thank the authors to have significantly improved their manuscript about "Long-read sequencing of the coffee bean transcriptome that reveals the diversity of full length transcripts". I have only minor modifications to suggest.

1) A General suggestion is to prefer "(Table 1)" instead of "(see Table 1)", for instance:
Line 233 were removed (see Table 1).
Line 237 quality based on Qcov, ID and sequence length (see Data description and Table 2)
Line 239 quality groups, respectively (see supporting information 1 Table S1)
Line 242 1,217 sequences with no hits to the four databases whereas in Table S1 it is written 1,213

Please note a further search with Rfam for non-coding sequences was processed after BLAST to the FOUR databases (1,217 sequences without hit). Four more sequences found hits, which results in 1,213 sequences instead of 1,217 (Table S1).

Line 294 (see Table 3)

All the "see" were removed as suggested in Line 94. 235, 239, 241, 258, 263, 267, 270, 280, 304, 315, 372, 393, 409.

2) A second general suggestion is to replace qcovs or qscov with qcov, for instance:
Line 182 filtered with query coverage (qcovs)
Supporting information 1
Table S1 BLAST output filtering with query coverage and cumulative identity. Qcovs
Qscov (%, ≥)

This has been changed to Qcovs in Line 183, 239 and Table S1.

3) Could you cite any reference about the "Cumulative identity that represents the identity length to the aligned length (AL)"?
There is no citation to cumulative identity and alignment length calculation as we have developed the python script. The alignment length is extracted and from NCBI BLAST result and incrementally calculated with the script.
The script has been submitted to Github as private file currently and will be available for public once this manuscript has published (Additional information). The script is also attached below for your information.

```
import xml.etree.ElementTree as ET
import pdb
import string
import argparse
import os
import time

#pdb.set_trace()

# Parse customized input parameters
```

```
parser = argparse.ArgumentParser(description='Input Identity(%) by your own.')
parser.add_argument('--Identity', type=float, default=0.0, help='Identity percent')
args = parser.parse_args()

Identity_condition = args.Identity


# import '.xml' to python
res = ET.parse('blast_outfmt5.xml').getroot()


# get all your sequence data into 'lst_Iteration'
lst_Iteration = res.findall('BlastOutput_iterations/Iteration')
print '# The number of "Iteration" (#yourRNA): ', len(lst_Iteration)


for iteration in lst_Iteration:
# get info about your sequences
iteration_iterNum = iteration.find('Iteration_iter-num').text
iteration_queryID = iteration.find('Iteration_query-def').text
Iteration_queryLen = iteration.find('Iteration_query-len').text

# get info about all 'Hit' (the index of subject)
lst_Hits = iteration.findall('Iteration_hits/Hit')

# for each matched subject, get info about alignments
flg_printQueryID = False
for hits in lst_Hits:
flg = True
Hit_len = hits.find('Hit_len').text
lst_hsp = hits.findall('Hit_hsps/Hsp')
name = hits.find('Hit_def').text
AL = 0
identity = 0
for hsp in lst_hsp:
align_len = hsp.find('Hsp_align-len').text
AL = AL + int(align_len)
identity = identity + int(hsp.find('Hsp_identity').text)
Iden = 1.0*100*identity/AL


# check for several conditions
if Iden < Identity_condition:
flg = False

# if the 'iteration' satisfies all these conditions, then output the name of 'iteration'
```

```
if flg == True:
flg_printQueryID = True

words = iteration_queryID.split(' ',1)
queryID = words[0]
print '[FOUND] The desired iteration name:',queryID
print '|- Name of Hit:', name, ' | len(Hit):',Hit_len,' | ',len(lst_hsp),'alignments (hsp) matched subject.'
print ' |-- Alignment length (AL, bp):',AL
print ' |-- Identity (% of match):',Iden
print '\n'

with open('result.txt','a') as result_file:
result_file.write('[FOUND] The desired iteration name: '+str(queryID) + '\n')
result_file.write('|- Name of Hit: ' + str(name) + ' | len(Hit):' + str(Hit_len) + ' | ' + str(len(lst_hsp)) + 'alignments (hsp) matched subject.' + '\n')
result_file.write(' |-- Alignment length (AL):' + str(AL) + '\n')
result_file.write(' |-- Identity (% of match):' + str(Iden) + '\n')
result_file.write('\n')
result_file.close()

break

if flg_printQueryID == True:
with open('SeqID.txt','a') as seqID_file:
seqID_file.write(str(queryID) + '\t' + str(Iteration_queryLen) + '\t' + str(Iden) + '\n')
seqID_file.close()
```

4) Could you precise in Table S1 that some numbers correspond to threshold values for instance replace "Qscov (%, ≥)" with "Qcov threshold (%, ≥)" ?

This has been modified.

5) Check and correct the number of "Putative novel genes"
Actually 1,217 should be replace by 1,213.
Line 376 The 1,217 sequences without hits to the FOUR databases
Also if it can help you I run a BLASTX againt Uniprot I found hits for 6 of your isoforms. I let you check which are significant according to your filtering criteria and update the datasets if necessary

```
nohup blast_cluster.pl --input 7_1271_novel_genes.fa --directory /homedir/sidibebocs/work/Blast/test2 --program blastx --evalue 1e-10 --output 7_1271_novel_gene_uniprot.tsv -q normal --num_seq_by_batch 15 --max_thread 96 --max_target_seq 1 --format 7 --database /work/BANK/uniprot/uniprot_taxonomy33090_20170301.faa > nohupCOFCA.out &
```

$ grep -v '^#' 7_1271_novel_gene_uniprot.tsv

Fields: query id, subject id, % identity, alignment length, mismatches, gap opens, q. start, q. end, s. start, s. end, evalue, bit score
c31471/f1p0/981 tr|A0A068UIH9|A0A068UIH9_COFCA 100.00 31 0 0 3 95 243 273 4e-11 68.6
c20564/f1p0/341 tr|A0A068U9S5|A0A068U9S5_COFCA 100.00 33 0 0 36 134 1 33 3e-14 68.9
c32926/f1p0/548 tr|A0A068TZN7|A0A068TZN7_COFCA 93.94 33 2 0 285 383 13 45 2e-13 68.9
c47713/f1p0/858 tr|K4C554|K4C554_SOLLC 83.33 36 6 0 787 680 4 39 5e-11 65.5
c77311/f1p0/2536 tr|A0A1J7GS48|A0A1J7GS48_LUPAN 71.79 39 11 0 88 204 328 366 1e-11 52.8
c77311/f1p0/2536 tr|A0A1J7GS48|A0A1J7GS48_LUPAN 93.33 15 1 0 44 88 313 327 1e-11 31.2
c77311/f1p0/2536 tr|A0A1J7GS48|A0A1J7GS48_LUPAN 84.62 13 2 0 12 50 302 314 1e-11 26.6
c77311/f1p0/2536 tr|A0A1J7GS48|A0A1J7GS48_LUPAN 84.62 13 2 0 195 233 363 375 1e-11 23.9
c150210/f1p0/5964 tr|A0A068UGC2|A0A068UGC2_COFCA 96.67 30 0 1 3460 3549 79 107 6e-15 55.1
c150210/f1p0/5964 tr|A0A068UGC2|A0A068UGC2_COFCA 95.83 24 1 0 3552 3623 109 132 6e-15 49.3
c150210/f1p0/5964 tr|A0A068UGC2|A0A068UGC2_COFCA 81.82 11 2 0 3428 3460 68 78 6e-15 23.9

These six genes are not in the list of the 1,213 novel genes. Please note the original list has been modified as sequence IDs in supporting information 2 (explained in Additional information).

6) Could you rephrase the sentence?
Line 429 Secondly, it would be interesting to
Line 430 determine the reason for more transcript variants identified is similar to those from the C.
Line 431 canephora sub-genome copies of the XMT and DXMT genes and their differential
Line 432 expression in tissues and at development stages.
This has been revised (Page Line 429-431).
