# Supplementary material for: Long-read sequencing of the coffee bean transcriptome reveals the diversity of full-length transcripts
Source: Gigascience. 2017 Aug 30;6(11):1–13. doi: 10.1093/gigascience/gix086 (PMC5737654; doi:10.1093/gigascience/gix086)
Supplement: Response-to-Reviewer-Comments_Revision-2.pdf [file gix086_response-to-reviewer-comments_revision-2.pdf]

Author's Response To Reviewer Comments

Details of sample preparation have been provided in expanded methods.
Supporting information has been updated.
