# Supplementary material for: Long-read sequencing of the coffee bean transcriptome reveals the diversity of full-length transcripts
Source: Gigascience. 2017 Aug 30;6(11):1–13. doi: 10.1093/gigascience/gix086 (PMC5737654; doi:10.1093/gigascience/gix086)
Supplement: Response-to-Reviewer-Comments_Revision-3.pdf [file gix086_response-to-reviewer-comments_revision-3.pdf]

Author's Response To Reviewer Comments

Citations to protocols now added
