# Supplementary material for: Long-read sequencing of the coffee bean transcriptome reveals the diversity of full-length transcripts
Source: Gigascience. 2017 Aug 30;6(11):1–13. doi: 10.1093/gigascience/gix086 (PMC5737654; doi:10.1093/gigascience/gix086)
Supplement: Reviewer-1-Report-(Original-Submission).pdf [file gix086_reviewer-1-report-(original-submission).pdf]

Reviewer Report

Title: Long-read sequencing of the coffee bean transcriptome reveals the diversity of full length transcripts

Version: Original Submission **Date:** 3/12/2017

Reviewer name: Sandeep Chakraborty

Reviewer Comments to Author:

Cheng et al. have presented a manuscript on a very relevant topic - third generation sequencing on an economically important crop. While I wholeheartedly agree that long read sequencing will address several
assembly, and downstream, problems - resulting in a better understanding of several genetic aspects of any organism, there are several inaccuracies in the current manuscript that need to be addressed before publication.

1. There are several transcripts (about 40) from the pathogenic fungus genus Fusarium - C117579.F1P0.6198
is one such example. Methods for quickly detecting metagenomic transcripts have been elucidated in http://biorxiv.org/content/early/2016/10/04/079186.
https://www.ncbi.nlm.nih.gov/pmc/articles/PMC1317314/ is just one of previous work on this pathogens
effect on the coffee yield.

2. "In total, 96,415 coffee-LRS isoform sequences were recovered after sequences representing chloroplast,
mitochondrial and ribosomal transcripts" - chloroplastic sequences have not been removed completely. C118772.F1P0.4602 is one example. There are about 20 such transcripts.

3. "After filtering LRS isoform sequences with NCBI-nr, 5,667 sequences without a hit were" - in the absence of a reference genome, absence in the NCBI-nr database can not be inferred as novel genes. It might be contamination from an hitherto un-sequenced organism. If novel does not imply novel in coffee, this needs to be clearly stated.

Matching the 96k transcripts to ncbi-nr is grossly inefficient (and inaccurate, as observed with the matches
to the fungus). I have validated the provided data, with emphasis on both accuracy and computational times.
Most of the analysis done here is on a small workstation (8GB ram) within a day. The transcript names

have been changed to replace "/" with a "." to allow for Unix style file names. In the search for novel genes
and annotation started with the cds from related genomes (coffee-genome.org/), and followed by transcripts
from related plant genomes (about 8 - vitis, malus, sesame etc). This quickly identified homologous genes -
thus reducing computational times significantly as compared to matching to ncbi-nr. The unmatched genes
was reduced to about 1000 from 96k in a few hours.

In my opinion, 1E-05 is too high a value for significance for nucleotide matching. The exact threshold is can be debated till the end of the world. One example is C107709.F1P0.5231 (Length=5231) matching to XM 010247806.2 (Length=8430) from lotus with Evalue=4e-10. The alignment seems too small to be considered significant.
AAACCGTTTTCATCACTTCAAAGATGGATTTTGTTCTTGTGGAGATTAT-GGT
AAATCGATTTCATCACTTCAAAGATGGATTTTGTTCTTGTGGAGATTATTGGT
One possible way to address this might be to split matches into low, medium and high significance.
The protein section for xmt genes (Table 1) is accurate. However, the absence of an xmt2 gene needs to be discussed since there is a given gene for coffea arabica, with which there is a lower (but still significant)
homology in C30813.F1P0.1617. The reason for this needs to be discussed.

Minor comments. There are several grammatical and typographical errors. I am mentioning the first three.
1. Abstract: uncertain should be uncertainty.
2. Abstract: frams - frames.
3. Abstract: toolto - tool to

Methods

Are the methods appropriate to the aims of the study, are they well described, and are necessary controls included? Yes

Conclusions

Are the conclusions adequately supported by the data shown? No

Reporting Standards

Does the manuscript adhere to the journal's guidelines on [minimum standards of reporting?]() YesChoose an item.

Statistics

Are you able to assess all statistics in the manuscript, including the appropriateness of statistical tests used? No, and I do not feel adequately qualified to assess the statistics.

Quality of Written English

Please indicate the quality of language in the manuscript: Not suitable for publication unless extensively edited

Declaration of Competing Interests

Please complete a declaration of competing interests, considering the following questions:

- Have you in the past five years received reimbursements, fees, funding, or salary from an organisation that may in any way gain or lose financially from the publication of this manuscript, either now or in the future?
- Do you hold any stocks or shares in an organisation that may in any way gain or lose financially from the publication of this manuscript, either now or in the future?
- Do you hold or are you currently applying for any patents relating to the content of the manuscript?
- Have you received reimbursements, fees, funding, or salary from an organization that holds or has applied for patents relating to the content of the manuscript?
- Do you have any other financial competing interests?
- Do you have any non-financial competing interests in relation to this paper?

If you can answer no to all of the above, write 'I declare that I have no competing interests' below. If your reply is yes to any, please give details below.

I declare that I have no competing interests

I agree to the open peer review policy of the journal. I understand that my name will be included on my report to the authors and, if the manuscript is accepted for publication, my named report including any attachments I upload will be posted on the website along with the authors' responses. I agree for my report to be made available under an Open Access Creative Commons CC-BY license (http://creativecommons.org/licenses/by/4.0/). I understand that any comments which I do not wish to be included in my named report can be included as confidential comments to the editors, which will not be published.

I agree to the open peer review policy of the journal

To further support our reviewers, we have joined with Publons, where you can gain additional credit to further highlight your hard work (see: https://publons.com/journal/530/gigascience). On publication of

this paper, your review will be automatically added to Publons, you can then choose whether or not to claim your Publons credit. I understand this statement.

Yes
