# Supplementary material for: Long-read sequencing of the coffee bean transcriptome reveals the diversity of full-length transcripts
Source: Gigascience. 2017 Aug 30;6(11):1–13. doi: 10.1093/gigascience/gix086 (PMC5737654; doi:10.1093/gigascience/gix086)
Supplement: Reviewer-1-Report-(Revision-1).pdf [file gix086_reviewer-1-report-(revision-1).pdf]

Reviewer Report

Title: Long-read sequencing of the coffee bean transcriptome reveals the diversity of full length transcripts

Version: Revision 1 **Date:** 7/1/2017

Reviewer name: Sandeep Chakraborty

Reviewer Comments to Author:

Cheng, et al. have satisfactorily responded to my comments with respect to contamination and xmt2 gene (my mistake).

However, significant discrepancies in their data still exist - and need to be addressed.

1) The number 1217 of "novel genes" in the paper, and those provided in 7_1271_novel_genes.fa (n=1271) dont match.
Maybe a typo (71 becomes 17?).

2) There are transcripts with really small ORFS - these cannot be taken as genes without proof

>C35828.F1P0.435
MKLGFLGKGFGLKTEDERQKMKKQRRGC
>C14734.F1P0.431
MMTKSPLHLYVARFYTNYSTLETSTPS
>C23681.F3P0.964
MGMNMIYMDFVEGKGFLVEAKWTAFSSPE

There should be a reasonable cutoff - maybe 60.

3) Finally, and most importantly, they are not novel : C57465.F1P0.755 ORF 2 matches to:
https://www.ncbi.nlm.nih.gov/protein/661881444?report=genbank&log$=prottop&blast_rank=1&RID=MFJCCGRX016
with a 100% match.
"The 1,217 sequences without hits to the FOUR databases (NR plant proteins" is therefore incorrect.

4) Also, the 145 sequences in the "6_Long_non-coding_RNAs.fasta" all have long ORF's (>100).
c14217_f2p0_996_1 also an ORF with significant match (1E-25) to XP_019192529.1 (
PREDICTED: uncharacterized protein C6G9.01c-like [Ipomoea nil])

Most of the "novel genes" seem to be long non-coding RNA.

Methods

Are the methods appropriate to the aims of the study, are they well described, and are necessary controls included? Yes

Conclusions

Are the conclusions adequately supported by the data shown? No

Reporting Standards

Does the manuscript adhere to the journal's guidelines on minimum standards of reporting? YesChoose an item.

Statistics

Are you able to assess all statistics in the manuscript, including the appropriateness of statistical tests used? Yes, and I have assessed the statistics in my report.

Quality of Written English

Please indicate the quality of language in the manuscript: Acceptable

Declaration of Competing Interests

Please complete a declaration of competing interests, considering the following questions:

- Have you in the past five years received reimbursements, fees, funding, or salary from an organisation that may in any way gain or lose financially from the publication of this manuscript, either now or in the future?
- Do you hold any stocks or shares in an organisation that may in any way gain or lose financially from the publication of this manuscript, either now or in the future?
- Do you hold or are you currently applying for any patents relating to the content of the manuscript?
- Have you received reimbursements, fees, funding, or salary from an organization that holds or has applied for patents relating to the content of the manuscript?
- Do you have any other financial competing interests?
- Do you have any non-financial competing interests in relation to this paper?

If you can answer no to all of the above, write 'I declare that I have no competing interests' below. If your reply is yes to any, please give details below.

I declare that I have no competing interests

I agree to the open peer review policy of the journal. I understand that my name will be included on my report to the authors and, if the manuscript is accepted for publication, my named report including any attachments I upload will be posted on the website along with the authors' responses. I agree for my report to be made available under an Open Access Creative Commons CC-BY license (http://creativecommons.org/licenses/by/4.0/). I understand that any comments which I do not wish to be included in my named report can be included as confidential comments to the editors, which will not be published.

I agree to the open peer review policy of the journal

To further support our reviewers, we have joined with Publons, where you can gain additional credit to further highlight your hard work (see: https://publons.com/journal/530/gigascience). On publication of this paper, your review will be automatically added to Publons, you can then choose whether or not to claim your Publons credit. I understand this statement.

Yes
