# Supplementary material for: Long-read sequencing of the coffee bean transcriptome reveals the diversity of full-length transcripts
Source: Gigascience. 2017 Aug 30;6(11):1–13. doi: 10.1093/gigascience/gix086 (PMC5737654; doi:10.1093/gigascience/gix086)
Supplement: Reviewer-2-Report-(Original-Submission).pdf [file gix086_reviewer-2-report-(original-submission).pdf]

Reviewer Report

Title: Long-read sequencing of the coffee bean transcriptome reveals the diversity of full length transcripts

Version: Original Submission **Date:** 3/18/2017

Reviewer name: Stephanie Bocs

Reviewer Comments to Author:

This manuscript reports a long-read sequencing of coffee bean transcriptomes.

This resource provides a reference transcriptome for the community working on the genetic improvement of the coffee tree. The study is interesting because it covers multiple aspects: caffeine and sucrose biosynthesis pathways, long and rare transcripts, novel genes, ORFs in 5'UTR. I encourage the authors to go a step further in their analyses to better promote their work.

*Major comments

1) In the background part or in the result part, I recommend defining terms important for this study.
a) For instance, regarding the term of 'transcript isoform', if I understood well the point of view of the authors in the context of polyploid species, transcript isoforms of a gene should represent splice variants (alternative transcripts), alleles, homoeologs but not close paralogs (Childs et al. 2014). They can also have a look at this publication (Gutierrez-Gonzalez et al. 2013) where Gutierrez et al. defined the terms of 'transcript isoform' or 'transcript', 'locus' or 'loci', 'splice variants', 'homoalleles'.
b) Also, I found that some important terms for the evolutionary process of plant polyploidization are missing. For instance, I would better understand the sentences "Diversification or specialisation may alter the nature of the gene product (e.g. encoded protein sequence) or the pattern of expression (e.g. tissue specificity of expression) of genes from each subgenome. Moreover, the copy number of genes in each sub-genome may be altered or the gene may even be deleted completely from some sub-genomes" if the authors add the terms of subfunctionalization, neofunctionalization and pseudogeneization (Levasseur and Pontarotti 2011).

2) Most of the methods are appropriate to the aims of the study but three points should be improved.
a) To identify the query sequences having a BLASTN hit, the e-value is not satisfying parameter because it depends on the size of the databank and thus is not comparable between several BLAST analyses. Instead, other parameters could be used such as AL, CIP and CALP (Salse et al. 2008) or the identity percentage, qcov and scov (Mbeguie et al. 2009). They will allow filtering results more precisely and independently of the databank.
b) A GMAP (Wu and Watanabe 2005) analysis of the coffee LRS transcriptome against the C. canephora genome could help to (i) separate homeologs from paralogs for each locus, (ii) detect missing genes in C. canephora annotation (see minor suggestion 4) and (iii) define clusters that could then be refined to

separate the homeologs according to their origin (see major comment 3). The authors can have a look for instance on Polycat (Page et al. 2013) and Homeosplitter (Ranwez et al. 2013) methodologies developed for allotetraploid crops.
c) For coding sequence analysis, Framedp (Gouzy et al. 2009) or prot4EST (Wasmuth and Blaxter 2004) would be more adapted than the ORF prediction.

3) The data produced should be sufficient to support the discussion. The authors claim that "This study clearly shows the expression of sub-genome copies accounting for much of the polyploid diversity" but the analysis is not thorough enough. The reader would expect to have access to at least a set of sub-genome specific isoforms (i.e. couples of transcript isoforms for each locus tagged as derived from C. canephora or from C. eugenioides). Indeed, it could be too difficult to phase the four haplotypes of this allotetraploid LRS transcriptome if the two haplotypes of each sub-genome are very close. I do not know if PacBio Iso-Seq™ community could help the authors for this analysis but maybe the HapIso methodology (Mangul et al. 2016) could be used making the assumption that the study of an allotetraploid can be reduced to the study of a diploid. So a 2-means clustering should be sufficient even if a 4-means could also be tested.

*Minor suggestions

1) Replace 'cds' with 'CDS'
2) Replace 'eg' with 'e.g.'
3) Under ten (1, 2, 3, 4, 5, 6, 7, 8, 9 and 10), write number in letters (one, two, three, four, five, six, seven, height, nine, ten).
4) The sentence "Most sequences matched tobacco probably because the tobacco database is more extensive and well annotated than those of other related species, like C. canephora." is not at the most suitable location in the text because it talks about tobacco in paragraph about Coffee database. Also, the speculation about the annotation quality of C. canephora is not well supported. The GMAP analysis, suggested in the major point 2c, could also allow estimating the number of missing genes in the C. canephora annotation.
5) Each time, precise the kind of BLAST analysis, e.g. BLASTN.
6) The figure 4f should be removed as it the same as figure 5.
7) See other minor suggestions directly in the text of the attached file.

Childs KL, Nandety A, Hirsch CN, Góngora-Castillo E, Schmutz J, Kaeppler SM, Casler MD, Buell CR. 2014. Generation of Transcript Assemblies and Identification of Single Nucleotide Polymorphisms from Seven Lowland and Upland Cultivars of Switchgrass. The Plant Genome 7:2.

Gouzy J, Carrere S, Schiex T. 2009. FrameDP: sensitive peptide detection on noisy matured sequences. Bioinformatics 25: 670-671.

Gutierrez-Gonzalez JJ, Tu ZJ, Garvin DF. 2013. Analysis and annotation of the hexaploid oat seed transcriptome. BMC Genomics 14: 471.

Levasseur A, Pontarotti P. 2011. The role of duplications in the evolution of genomes highlights the need for evolutionary-based approaches in comparative genomics. Biol Direct 6: 11.

Mangul S, Yang HT, Hormozdiari F, Tseng E, Zelikovsky A, Eskin A. 2016. HapIso : An Accurate Method for the Haplotype-Specific Isoforms Reconstruction from Long Single-Molecule Reads. CSH BioRXiv https://doi.org/10.1101/050906.

Mbeguie AMD, Hubert O, Baurens FC, Matsumoto T, Chillet M, Fils-Lycaon B, Bocs S. 2009. Expression patterns of cell wall-modifying genes from banana during fruit ripening and in relationship with finger drop. J Exp Bot 60: 2021-2034.

Page JT, Gingle AR, Udall JA. 2013. PolyCat: a resource for genome categorization of sequencing reads from allopolyploid organisms. G3 3: 517-525.

Ranwez V, Holtz Y, Sarah G, Ardisson M, Santoni S, Glemin S, Tavaud-Pirra M, David J. 2013. Disentangling homeologous contigs in allo-tetraploid assembly: application to durum wheat. BMC Bioinformatics 14 Suppl 15: S15.

Salse J, Bolot S, Throude M, Jouffe V, Piegu B, Quraishi UM, Calcagno T, Cooke R, Delseny M, Feuillet C. 2008. Identification and Characterization of Shared Duplications between Rice and Wheat Provide New Insight into Grass Genome Evolution. Plant Cell 20: 11-24.

Wasmuth JD, Blaxter ML. 2004. prot4EST: translating expressed sequence tags from neglected genomes. BMC Bioinformatics 5: 187.

Wu TD, Watanabe CK. 2005. GMAP: a genomic mapping and alignment program for mRNA and EST sequences. Bioinformatics 21: 1859-1875.

Methods

Are the methods appropriate to the aims of the study, are they well described, and are necessary controls included? No

Conclusions

Are the conclusions adequately supported by the data shown? No

Reporting Standards

Does the manuscript adhere to the journal's guidelines on minimum standards of reporting? YesChoose an item.

Statistics

Are you able to assess all statistics in the manuscript, including the appropriateness of statistical tests used? There are no statistics in the manuscript.

Quality of Written English

Please indicate the quality of language in the manuscript: Needs some language corrections before being published

Declaration of Competing Interests

Please complete a declaration of competing interests, considering the following questions:

- Have you in the past five years received reimbursements, fees, funding, or salary from an organisation that may in any way gain or lose financially from the publication of this manuscript, either now or in the future?
- Do you hold any stocks or shares in an organisation that may in any way gain or lose financially from the publication of this manuscript, either now or in the future?
- Do you hold or are you currently applying for any patents relating to the content of the manuscript?
- Have you received reimbursements, fees, funding, or salary from an organization that holds or has applied for patents relating to the content of the manuscript?
- Do you have any other financial competing interests?
- Do you have any non-financial competing interests in relation to this paper?

If you can answer no to all of the above, write 'I declare that I have no competing interests' below. If your reply is yes to any, please give details below.

I declare that I have no competing interests

I agree to the open peer review policy of the journal. I understand that my name will be included on my report to the authors and, if the manuscript is accepted for publication, my named report including any attachments I upload will be posted on the website along with the authors' responses. I agree for my report to be made available under an Open Access Creative Commons CC-BY license (http://creativecommons.org/licenses/by/4.0/). I understand that any comments which I do not wish to be included in my named report can be included as confidential comments to the editors, which will not be published.

I agree to the open peer review policy of the journal

To further support our reviewers, we have joined with Publons, where you can gain additional credit to further highlight your hard work (see: https://publons.com/journal/530/gigascience). On publication of this paper, your review will be automatically added to Publons, you can then choose whether or not to claim your Publons credit. I understand this statement.

Yes
