# Supplementary material for: Long-read sequencing of the coffee bean transcriptome reveals the diversity of full-length transcripts
Source: Gigascience. 2017 Aug 30;6(11):1–13. doi: 10.1093/gigascience/gix086 (PMC5737654; doi:10.1093/gigascience/gix086)
Supplement: Reviewer-2-Report-(Revision-1).pdf [file gix086_reviewer-2-report-(revision-1).pdf]

Reviewer Report

Title: Long-read sequencing of the coffee bean transcriptome reveals the diversity of full length transcripts

Version: Revision 1 **Date:** 7/5/2017

Reviewer name: Stephanie Bocs

Reviewer Comments to Author:

I greatly thank the authors to have significantly improved their manuscript about "Long-read sequencing of the coffee bean transcriptome that reveals the diversity of full length transcripts". I have only minor modifications to suggest.

1) A General suggestion is to prefer "(Table 1)" instead of "(see Table 1)", for instance:
Line 233 were removed (see Table 1).
Line 237 quality based on Qcov, ID and sequence length (see Data description and Table 2)
Line 239 quality groups, respectively (see supporting information 1 Table S1)
Line 242 1,217 sequences with no hits to the four databases whereas in Table S1 it is written 1,213
Line 294 (see Table 3)

2) A second general suggestion is to replace qcovs or qscov with qcov, for instance:
Line 182 filtered with query coverage (qcovs)
Supporting information 1
Table S1 BLAST output filtering with query coverage and cumulative identity. Qcovs
Qscov (%, ≥)

3) Could you cite any reference about the "Cumulative identity that represents the identity length to the aligned length (AL)"?

4) Could you precise in Table S1 that some numbers correspond to threshold values for instance replace "Qscov (%, ≥)" with "Qcov threshold (%, ≥)" ?

5) Check and correct the number of "Putative novel genes"
Actually 1,217 should be replace by 1,213.
Line 376 The 1,217 sequences without hits to the FOUR databases
Also if it can help you I run a BLASTX againt Uniprot I found hits for 6 of your isoforms. I let you check which are significant according to your filtering criteria and update the datasets if necessary
nohup blast_cluster.pl --input 7_1271_novel_genes.fa --directory /homedir/sidibebocs/work/Blast/test2 --program blastx --evalue 1e-10 --output 7_1271_novel_gene_uniprot.tsv -q normal --num_seq_by_batch 15 --max_thread 96 --max_target_seq 1 --format 7 --database /work/BANK/uniprot/uniprot_taxonomy33090_20170301.faa > nohupCOFCA.out &

```
$ grep -v '^#' 7_1271_novel_gene_uniprot.tsv
# Fields: query id, subject id, % identity, alignment length, mismatches, gap opens, q. start, q. end, s. start, s. end, evalue, bit score
c31471/f1p0/981 tr|A0A068UIH9|A0A068UIH9_COFCA 100.00 31 0 0 3 95 243 273 4e-11 68.6
c20564/f1p0/341 tr|A0A068U9S5|A0A068U9S5_COFCA 100.00 33 0 0 36 134 1 33 3e-14 68.9
c32926/f1p0/548 tr|A0A068TZN7|A0A068TZN7_COFCA 93.94 33 2 0 285 383 13 45 2e-13 68.9
c47713/f1p0/858 tr|K4C554|K4C554_SOLLC 83.33 36 6 0 787 680 4 39 5e-11 65.5
c77311/f1p0/2536 tr|A0A1J7GS48|A0A1J7GS48_LUPAN 71.79 39 11 0 88 204 328 366 1e-11 52.8
c77311/f1p0/2536 tr|A0A1J7GS48|A0A1J7GS48_LUPAN 93.33 15 1 0 44 88 313 327 1e-11 31.2
c77311/f1p0/2536 tr|A0A1J7GS48|A0A1J7GS48_LUPAN 84.62 13 2 0 12 50 302 314 1e-11 26.6
c77311/f1p0/2536 tr|A0A1J7GS48|A0A1J7GS48_LUPAN 84.62 13 2 0 195 233 363 375 1e-11 23.9
c150210/f1p0/5964 tr|A0A068UGC2|A0A068UGC2_COFCA 96.67 30 0 1 3460 3549 79 107 6e-15 55.1
c150210/f1p0/5964 tr|A0A068UGC2|A0A068UGC2_COFCA 95.83 24 1 0 3552 3623 109 132 6e-15 49.3
c150210/f1p0/5964 tr|A0A068UGC2|A0A068UGC2_COFCA 81.82 11 2 0 3428 3460 68 78 6e-15 23.9
```

6) Could you rephrase the sentence?
Line 429 Secondly, it would be interesting to
Line 430 determine the reason for more transcript variants identified is similar to those from the C.
Line 431 canephora sub-genome copies of the XMT and DXMT genes and their differential
Line 432 expression in tissues and at development stages.

Methods

Are the methods appropriate to the aims of the study, are they well described, and are necessary controls included? Yes

Conclusions

Are the conclusions adequately supported by the data shown? Yes

Reporting Standards

Does the manuscript adhere to the journal's guidelines on minimum standards of reporting? YesChoose an item.

Statistics

Are you able to assess all statistics in the manuscript, including the appropriateness of statistical tests used? There are no statistics in the manuscript.

Quality of Written English

Please indicate the quality of language in the manuscript: Needs some language corrections before being published

Declaration of Competing Interests

Please complete a declaration of competing interests, considering the following questions:

- Have you in the past five years received reimbursements, fees, funding, or salary from an organisation that may in any way gain or lose financially from the publication of this manuscript, either now or in the future?
- Do you hold any stocks or shares in an organisation that may in any way gain or lose financially from the publication of this manuscript, either now or in the future?
- Do you hold or are you currently applying for any patents relating to the content of the manuscript?
- Have you received reimbursements, fees, funding, or salary from an organization that holds or has applied for patents relating to the content of the manuscript?
- Do you have any other financial competing interests?
- Do you have any non-financial competing interests in relation to this paper?

If you can answer no to all of the above, write 'I declare that I have no competing interests' below. If your reply is yes to any, please give details below.

I declare that I have no competing interests

I agree to the open peer review policy of the journal. I understand that my name will be included on my report to the authors and, if the manuscript is accepted for publication, my named report including any attachments I upload will be posted on the website along with the authors' responses. I agree for my report to be made available under an Open Access Creative Commons CC-BY license (http://creativecommons.org/licenses/by/4.0/). I understand that any comments which I do not wish to be included in my named report can be included as confidential comments to the editors, which will not be published.

I agree to the open peer review policy of the journal

To further support our reviewers, we have joined with Publons, where you can gain additional credit to further highlight your hard work (see: https://publons.com/journal/530/gigascience). On publication of this paper, your review will be automatically added to Publons, you can then choose whether or not to claim your Publons credit. I understand this statement.

Yes
